# Asymmetric addition of α-branched cyclic ketones to allenamides catalyzed by a chiral phosphoric acid†
†Electronic supplementary information (ESI) available. See DOI: 10.1039/c5sc04202j


**DOI:** 10.1039/c5sc04202j

**Published:** 2016-01-19

**Authors:** Xiaoyu Yang, F. Dean Toste

**Affiliations:** a Department of Chemistry , University of California , Berkeley , California 94720 , USA . Email: fdtoste@Berkeley.edu

## Abstract

We describe the asymmetric addition of unactivated α-branched cyclic ketones to allenamides catalyzed by a chiral phosphoric acid, generating an all-carbon quaternary stereocenter with broad substrate scope and high enantioselectivity. The products are easily transformed into their corresponding 1,5- and 1,4-ketoaldehyde derivatives, which are both important building blocks in organic synthesis.

## Abstract 2

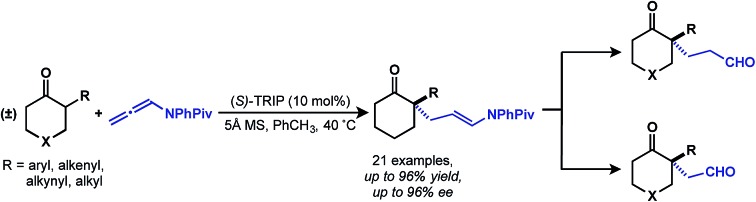
Asymmetric addition of unactivated α-branched cyclic ketones to allenamides catalyzed by a chiral phosphoric acid catalyst, generates an all-carbon quaternary stereocenter with broad substrate scope and high enantioselectivity.

Cyclic ketones bearing an all-carbon quaternary stereocenter at the α-position are versatile building blocks in organic synthesis and exist in a variety of natural products.^1^ While allylic alkylation and direct alkylation have been applied to the enantioselective synthesis these compounds,^2^–^4^ Michael addition to conjugated olefins represents one of the most attractive methods for the formation of C–C bonds adjacent to ketones.^5^ Among the several types of Michael acceptors, α,β-unsaturated carbonyl compounds have attracted significant research interest because of their wide availability in organic synthesis. Therefore, many asymmetric catalytic systems using ketones as nucleophiles have been developed, including Lewis acid,^6^ Brønsted base,^7^ phase-transfer,^8^ phosphine^9^ and phosphoric acid or phosphate catalysis.^10^ Despite these advances, the majority of ketone substrates have been limited to activated functionality, such as β-keto esters, 2-oxindoles and others. For unactivated α-branched cyclic ketones, use of a preformed imine as a Michael donor is a reliable solution;^11^ however, the requirement for stoichiometric amounts of chiral amine and the additional steps of imine preparation and hydrolysis limit the utility of this protocol. A catalytic version of this reaction was recently achieved by Carter and co-workers, who reported the asymmetric addition of 2-alkyl cyclic ketones to alkyl acrylates catalyzed by a bifunctional catalyst system.^12^ However, the cyclic ketones were limited to 2-methyl and 2-ethyl substitution, which may be attributed to the steric difficulties for formation of the enamine intermediate.

Very recently, we reported on the direct asymmetric amination of α-branched cyclic ketones catalyzed by a chiral phosphoric acid to generate N-containing quaternary stereocenters from unactivated cyclic ketones.^13^ With our continuing interest in asymmetric functionalization of α-branched cyclic ketones,^13^,^14^ we turned our attention to potential Michael addition of these substrates to conjugated olefins. Unfortunately, addition of 2-phenylcyclohexanone to acrolein or methyl acrylate using (*S*)-TRIP as catalyst did not afford the desired addition product (Fig. 1a). Changing the acceptor to methyl vinyl ketone provided the Michael addition product in 44% yield with 19% ee. During the course of this work, List and co-workers reported on the asymmetric addition of α-branched cyclic ketones to alkyl vinyl ketones using (*S*)-TRIP as catalyst (Fig. 1b).^15^ However, bulky *tert*-butyl or isopropyl-substituted enones were required in order to achieve high enantioselectivities.

**Fig. 1:**
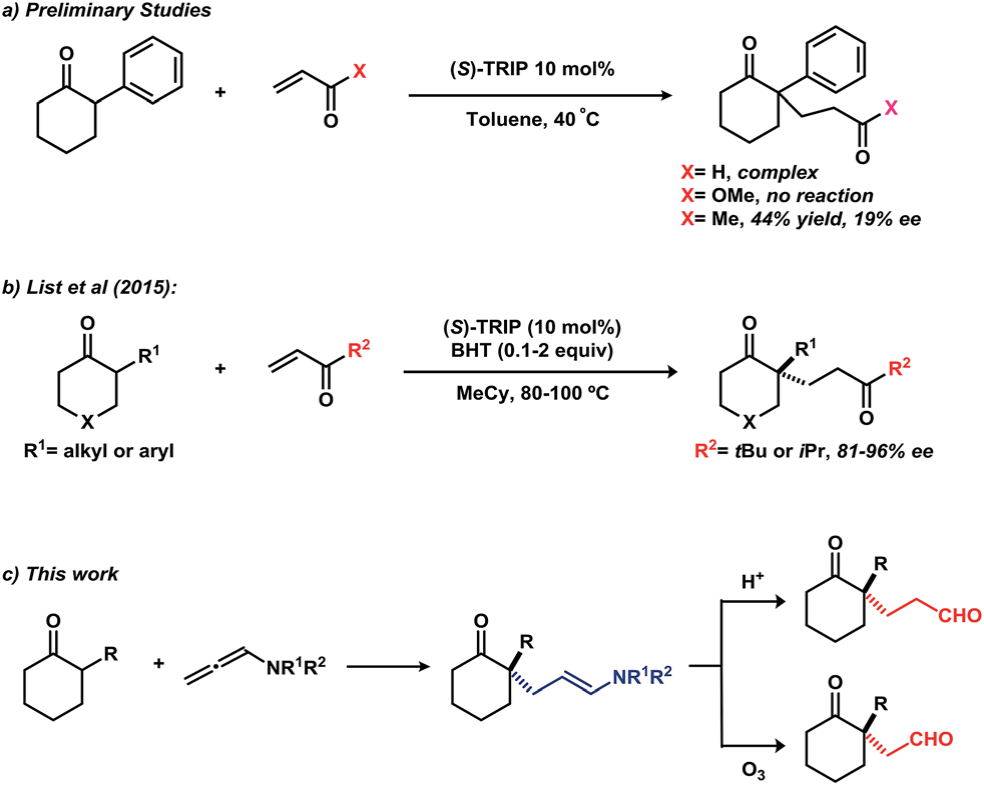
(a) Initial results on the addition of branched cyclic ketone to acrolein, methyl acrylate, and methyl vinyl ketone. (b) List's reports of asymmetric addition of branched cyclic ketones to *tert*-butyl vinyl ketone and isopropyl vinyl ketone. (c) Our proposal for the asymmetric addition of α-branched cyclic ketones to an allenamide and further transformation of the products.

We hypothesized that using a Michael acceptor surrogate might overcome these limitations. Inspired by the work of Bandini,^16^ we envisioned an allenamide might serve this role in a chiral phosphoric acid catalysed addition of α-branched cyclic ketones (Fig. 1c). Hydrolysis of the enamide moiety in the product would give the 1,5-keto aldehyde; the product of a formal Michael addition to an acrolein acceptor (*vide supra*). Furthermore, 1,4-keto aldehydes could be obtained by the oxidative cleavage of the enamide moiety. Together, these products represent important building blocks in synthesis of complex molecular structures.

We selected 2-phenylcyclohexanone (**1a**) as a model substrate. Various *N*-phenyl-allenamides (**2**, 2.0 equiv.) were first screened using (*S*)-TRIP (10 mol%) as a catalyst in toluene (1.0 M) at 40 °C, in the presence of 5 Å molecular sieves to inhibit the hydrolysis of the allenamides under these conditions (Table 1). The *N*-Ts- or *N*-Boc-*N*-phenyl-allenamides gave the products in very low yields under these conditions (entries 1 and 2). The *N*-Bz-*N*-phenyl-substituted allenamide provided the product in 52% yield with 70% ee (entry 3). Replacement of *N*-phenyl by *N*-benzyl-allenamide failed to afford desired product (entry 4). In contrast, the desired adduct was isolated in 56% yield with 82% ee when the amide group was changed from a benzamide to an acetamide (entry 5). The enantioselectivity was further improved by increasing the steric bulk on the amide moiety. When *N*-pivaloyl-*N*-phenyl-allenamide was used, the product was obtained in 68% yield with 94% ee (entry 7). An examination of various solvents revealed that nonpolar solvents, such as benzene and xylenes, furnished the product with similar levels of selectivity to toluene, albeit in a slightly diminished yields (entries 8 and 9). Using dichloromethane as solvent or conducting the reaction neat, led to much lower yields of the products (entries 10 and 11). Chiral phosphoric acid catalysts were also evaluated; H_8_-TRIP or C_8_-TRIP gave the product with the identical enantioselectivity to TRIP, albeit in lower yield (entries 12 and 13). Changing the 3,3′-substitutions on phosphoric acids (CPA1 or TCYP) decreased the yield of the product (entries 14 and 15). Finally, in order to avoid diminished yields due to slow hydrolysis of the allenamide, we found adding the allenamide in two portions improved the yield to 72% (entry 16).

**Table 1:** Optimization of the reaction conditions^*a*^

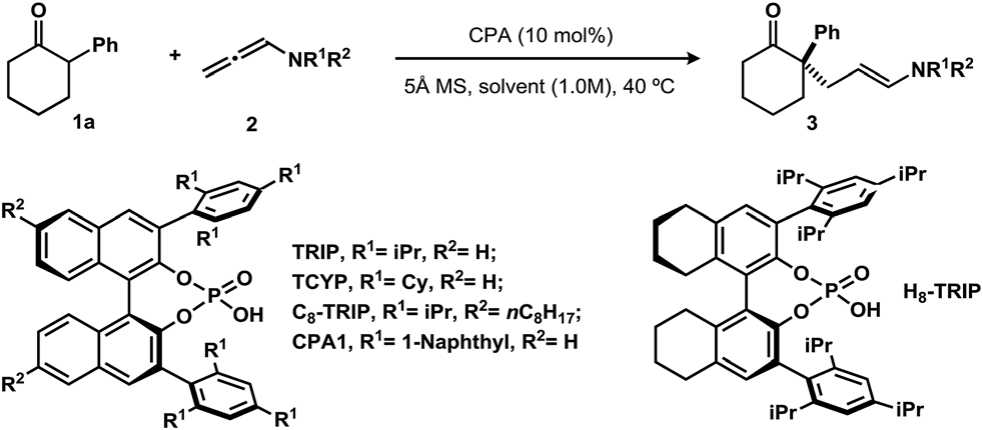						
Entry	R^1^	R^2^	Chiral phosphoric acids	Solvents	Yield^*b*^ [%]	ee^*c*^ [%]
1	Ph	Ts	(*S*)-TRIP	Toluene	7%	ND
2	Ph	Boc	(*S*)-TRIP	Toluene	17%	ND
3	Ph	Bz	(*S*)-TRIP	Toluene	52%	70%
4	Bn	Bz	(*S*)-TRIP	Toluene	NR	ND
5	Ph	Ac	(*S*)-TRIP	Toluene	56%	82%
6	Ph	iPrCO-	(*S*)-TRIP	Toluene	68%	90%
7	Ph	Piv	(*S*)-TRIP	Toluene	68%	94%
8	Ph	Piv	(*S*)-TRIP	Benzene	57%	94%
9	Ph	Piv	(*S*)-TRIP	Xylenes	62%	94%
10	Ph	Piv	(*S*)-TRIP	DCM	15%	86%
11	Ph	Piv	(*S*)-TRIP	-none-	15%	86%
12	Ph	Piv	(*R*)-H_8_-TRIP	Toluene	62%	–94%
13	Ph	Piv	(*R*)-C_8_-TRIP	Toluene	43%	–94%
14	Ph	Piv	(S)-CPA1	Toluene	19%	73%
15	Ph	Piv	(*R*)-TCYP	Toluene	<10%	ND
***16*** ^*d*^	***Ph***	***Piv***	***(S)-TRIP***	***Toluene***	***72%***	***94%***

^*a*^Reaction conditions, unless otherwise specified: **1a** (0.1 mmol). **2** (0.2 mmol), catalyst (10 mol%), 30 mg 5 Å molecular sieves, solvent (0.1 mL), 40 °C, 24–48 h.

^*b*^Isolated yield.

^*c*^The ee value was determined by HPLC analysis on a chiral stationary phase.

^*d*^The allenamide was added in two portions (1.0 equiv. at the beginning of the reaction, another 1.0 equiv. after 8 h).

With the optimized conditions in hand, the substrate scope was explored (Scheme 1). Various substituted arenes were well tolerated at the 2-position of cyclohexanone (including electron–neutral, electron-donating and electron-withdrawing arenes, **3a–3h**). The absolute configuration of the products was assigned as *S* by analogy to **3g**, whose configuration was established by comparison of the optical rotation of the aldehyde resulting from oxidative cleavage to that previously reported.^17^ The more challenging *o*-tolyl substituted cyclohexanone also gave the desired product (**3i**) with 87% ee, although 20 mol% of (*S*)-TRIP was needed in order to obtain high conversion. A heteroaryl-substituted analogue worked well, yielding the product (**3j**) in excellent yield and enantioselectivity. In contrast to our previous work in the amination of the thiophenylketone, no kinetic resolution of the starting ketone was observed under these conditions.^13^ The cyclopentanone (**3k**) and 4-pyranone (**3l**) derived substrates were also well tolerated in this transformation.

**Scheme 1:**
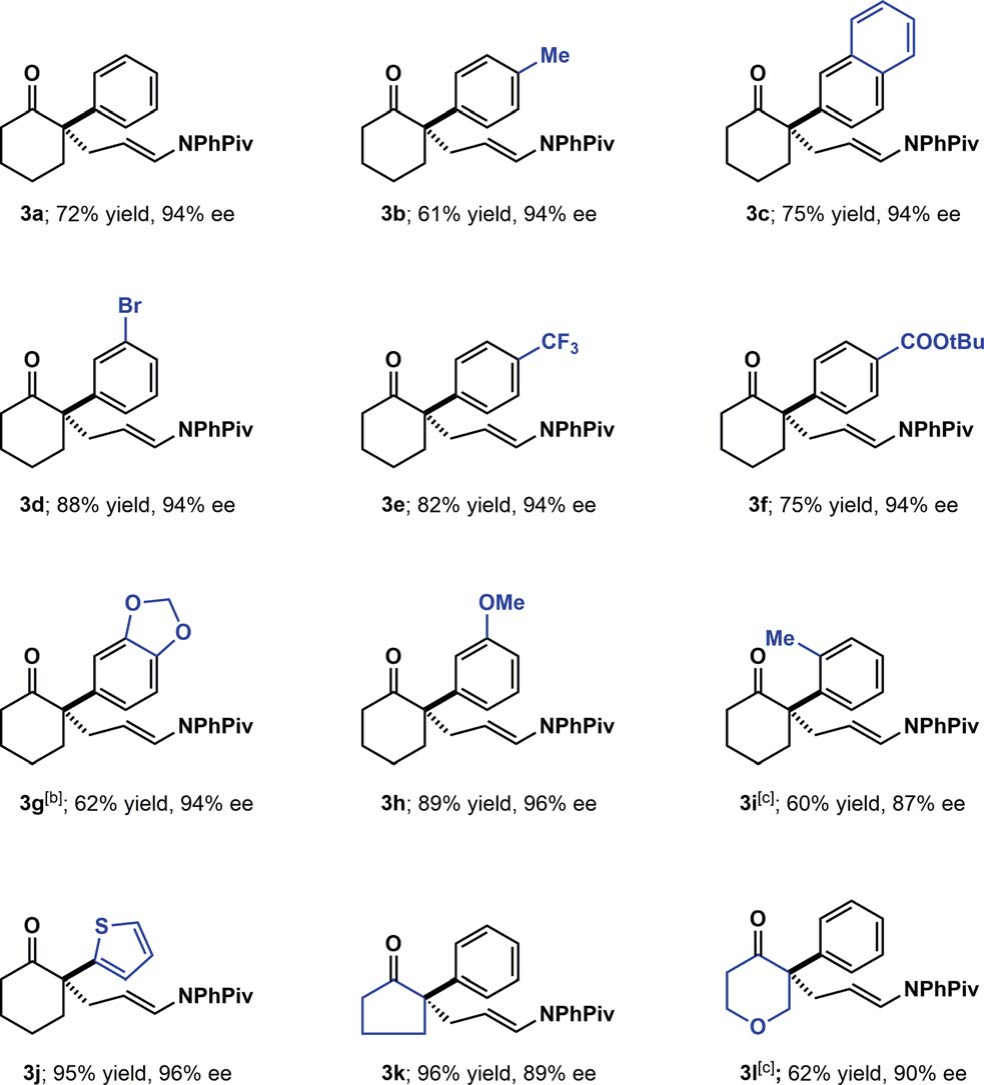
Substrate scope with α-aryl substitution. Reaction conditions, unless otherwise specified: **1** (0.3 mmol). **2** (0.6 mmol, added in two portions, 1.0 equiv. at the beginning of the reaction, another 1.0 equiv. after 8 h), (*S*)-TRIP (10 mol%), 90 mg 5 Å molecular sieves, toluene (0.3 mL), 40 °C, 24–48 h. Yields are isolated yields after chromatography. [b] The absolute configuration was determined by analogy to the derivative of **3g**, see SI.† [c] 20 mol% (*S*)-TRIP was used.

Having explored the compatibility of aryl substitution and modification of the cyclic ketone ring, we turned our attention to other substitution at the 2-position of cyclohexanone (Scheme 2). Various alkenyl substitutions were first investigated. The *trans*-styrenyl substituted ketone worked well, giving the desired product (**3m**) in 91% yield with 94% ee. The simple vinyl and 2-methyl propenyl-substituted substrates were compatible with these conditions (**3o** and **3p**). Products (**3n** and **3q**) derived from the sterically hindered *cis*-styrenyl and cyclohexenyl substituted ketones were also formed with excellent enantioselectivities, albeit in decreased yields. The 2-alkynyl substituted cyclohexanones were also evaluated under the standard conditions and found to provide the desired adducts (**3r** and **3s**) in good yields with high enantioselectivities. Lastly, we examined 2-alkyl cyclohexanones, which present challenges due to issues with regioselectivity and reactivity. Under the standard conditions, 2-methyl- and 2-butylcyclohexanone gave the desired products (**3t** and **3u**) in 70% and 78% ee, respectively; notably, we could not detect any of the undesired 6-substituted regioisomer.

**Scheme 2:**
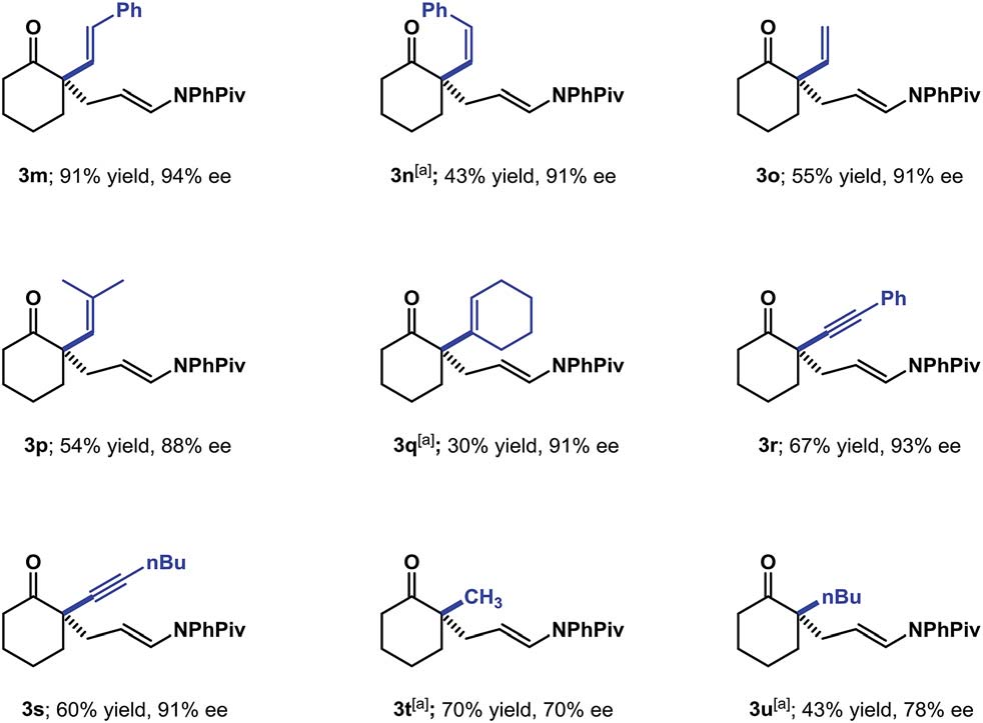
Substrate scope with α-alkenyl, alkynyl and alkyl substitution. Reaction conditions as indicated in Scheme 1. [a] 20 mol% (*S*)-TRIP was used.

To explore viability of this approach as an alternative to the direct enantioselective Michael reaction, the application of the adducts to the asymmetric synthesis of structurally diverse compounds was examined (Scheme 3). As expected, treatment of **3a** with 1 N HCl in Et_2_O for 3 min. afforded 1,5-keto aldehyde **4a** in 92% yield without any loss of enantioselectivity. Further intramolecular aldol reaction of **4a** under acid catalysis, followed by Dess–Martin periodinane-mediated oxidation furnished bicyclo[3.3.1]nonane^18^**5a** in 73% yield. Compound **4a** was transformed into bicyclic δ-lactone **6a** in 84% yield with 6 : 1 diastereoselectivity using catalytic amount of SmI_2_ and iPrSH.^19^ The relative configuration of major diastereoisomer was assigned as *cis* by NOESY analysis. Oxidative cleavage of the enamide C=C bond with O_3_/Me_2_S provided 1,4-keto aldehyde **7a** in 82% yield. Under reductive amination conditions, **7a** was smoothly transformed into the *cis*-aryl hydroindole (**8a**) core structure of a variety of *Amaryllidaceae*-type alkaloids.^4^,^20^

**Scheme 3:**
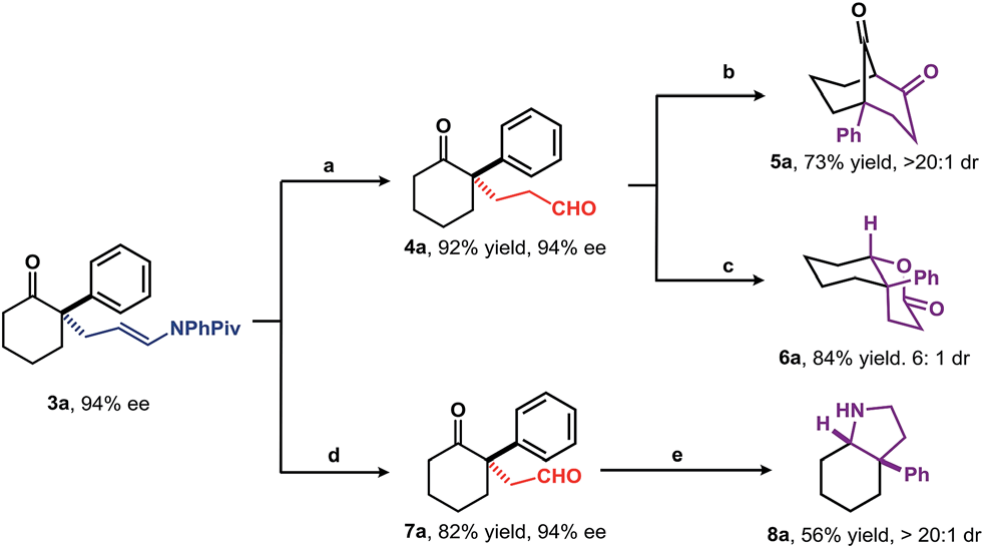
Synthetic transformations of the product **3a**. Reaction conditions: (a) 1 N HCl, Et_2_O, rt, 3 min; (b) (1) 2 N HCl, Et_2_O, rt, overnight; (2) DMP, DCM, rt; (c) SmI_2_, iPrSH, THF, rt; (d) O_3_, Me_2_S, DCM, –78 °C ∼ rt; (e) NH_4_OAc, NaBH_3_CN, HOAc, EtOH, rt. DMP = Dess–Martin periodinane.

## Conclusions

In summary, we have achieved the asymmetric addition of unactivated α-branched cyclic ketones to allenamides catalyzed by a chiral phosphoric acid catalyst. The reaction generates a chiral quaternary stereocenter with broad substrate scope, including various aryl, alkenyl, alkynyl, and alkyl substitution as well as modification of the cyclic ketone ring. The products are easily transformed into their corresponding 1,4- and 1,5-ketoaldehyde derivatives, which are both important building blocks in organic synthesis. Moreover, the two-step protocol to produce 1,5-ketoaldehyes represents a viable entry into the direct enantioselective Michael addition of unactivated ketones to acrolein.

## Supplementary Material

Supplementary information
