# Supplementary material for: Asymmetric addition of α-branched cyclic ketones to allenamides catalyzed by a chiral phosphoric acid
Source: Chem Sci. 2016 Jan 19;7(4):2653–6. doi: 10.1039/c5sc04202j (PMC5155590; doi:10.1039/c5sc04202j)

Electronic Supplementary Material (ESI) for Chemical Science.
This journal is © The Royal Society of Chemistry 2016

Asymmetric Addition of α-Branched Cyclic Ketones to Allenamides Catalyzed by a Chiral Phosphoric Acid

Xiaoyu Yang and F. Dean Toste*

Department of Chemistry, University of California, Berkeley, California 94720, United States.

Table of Contents

General information	S2
Synthesis of allenamides	S3
Synthesis of products	S5
Determination of the absolute stereochemistry of the products	S15
Transformations of products	S16
References	S19
HPLC traces	S20
NMR spectra	S47

General Information:

Unless otherwise noted, all commercial reagents were used without further purification. 5Å MS were dried in oven at 200 ºC overnight and used when after cooling to rt. Dichloromethane, toluene, ether, THF and triethylamine were purified by passage through an activated alumina column under argon. Thin-layer chromatography (TLC) analysis of reaction mixtures was performed using Merck silica gel 60 F254 TLC plates, and visualized under UV or by staining with ceric ammonium molybdate or potassium permanganate. Flash column chromatography was carried out on Merck Silica Gel 60 Å, 230 X 400 mesh. Nuclear magnetic resonance (NMR) spectra were recorded using Bruker AV-600, DRX-500, AV-500, AVQ-400, AVB-400 and AV-300 spectrometers. ^{1}H and ^{13}C chemical shifts are reported in ppm downfield of tetramethylsilane and referenced to residual solvent peak ($CHCl_3$; δH = 7.26 and δC = 77.16). Multiplicities are reported using the following abbreviations: s = singlet, d = doublet, t = triplet, q = quartet, m = multiplet, br = broad resonance. Mass spectral data were obtained from the Micro-Mass/Analytical Facility operated by the College of Chemistry, University of California, Berkeley. Enantiomeric excesses were determined on a Shimadzu VP Series Chiral HPLC using IA, IB, IC, OD, ASH, AD, OJ columns. The synthesis of phosphoric acids **(*S*)-TRIP**[1], **(*R*)-H$_8$_TRIP**,[2] **(*R*)-C$_8$_TRIP**[3], **(*S*)-PA1**[4] **and (*R*)-TCYP**[5] has been previously described. Racemic products were synthesized by carrying out the reactions using **(±)-TRIP** as catalyst.

Synthesis of allenamides:

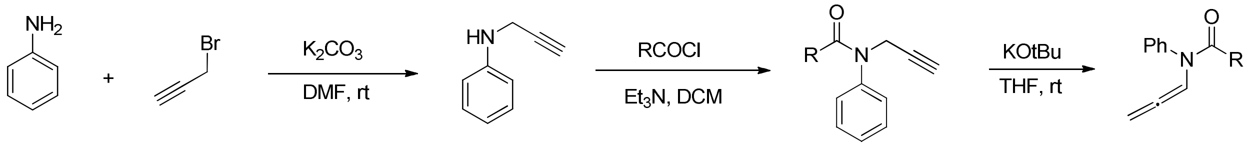

Typical procedure for preparation of allenamides:

To a solution of aniline (9.1 mL, 100 mmol) in DMF (150 mL) was added 3-bromopropyne (11.2 mL, 80% in toluene, 100 mmol) and K_2CO_3 at rt. After stirring overnight, the mixture was quenched with H_2O and extracted with Et_2O for 3 times. The combined organic layer was then successively washed with H_2O and brine, dried over Na_2SO_4, filtered and concentrated *in vacuo* to give a residue, which was purified by flash chromatography (20:1 – 15:1, Hexane/ Ethyl Ether) to afford the amine **S1**.

To the solution of **S1** (1.0 equiv.) in DCM (0.5 M) was added DIPEA (1.5 equiv.) and RCOCl (1.1 equiv.) at 0 °C. After stirring for 6 h, the mixture was diluted with DCM, washed with 1 N HCl solution and extracted with DCM for 3 times. The combined organic layer was then washed with satd. $NaHCO_3$ solution and brine, dried over Na_2SO_4, filtered and concentrated *in vacuo* to give a residue, which was purified by flash chromatography to afford the amide **S2**.

To the solution of **S2** (1.0 equiv.) in THF (0.2 M) was added KOtBu (0.1 equiv.) at rt. After consumption of the starting material by TLC monitoring, the mixture was filtered through a pad of silica gel. After concentration of the filtrate, the residue was purified by flash chromatography to afford the corresponding allenamides.

N-phenyl-*N*-(propa-1,2-dien-1-yl)acetamide

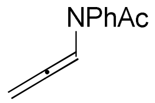

^{1}H NMR (400 MHz, $CDCl_3$) δ 7.69 (t, *J* = 6.5 Hz, 1H), 7.49 – 7.32 (m, 3H), 7.22 – 7.15 (m, 2H), 5.01 (s, 1H), 4.99 (s, 1H), 1.90 (s, 3H). ^{13}C NMR (100 MHz, $CDCl_3$) δ 202.67, 168.57, 140.16, 129.16, 128.52, 100.99, 86.46, 23.05. m/z HRMS (EI) found $[M]^+$ 173.0838, $C_{11}H_{11}NO^+$ requires 173.0841.

N-phenyl-*N*-(propa-1,2-dien-1-yl)benzamide

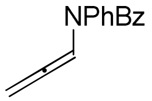

^{1}H NMR (400 MHz, $CDCl_3$) δ 7.65 (t, *J* = 6.6 Hz, 1H), 7.34 (d, *J* = 7.1 Hz, 2H), 7.28 – 7.22 (m, 3H), 7.21 – 7.15 (m, 3H), 7.08 (d, *J* = 7.6 Hz, 2H), 5.09 (s, 1H), 5.07 (s, 1H). ^{13}C NMR (100 MHz, $CDCl_3$) δ 202.80, 168.59, 140.39, 135.21, 130.11, 128.92, 128.89, 128.56, 127.88, 127.58, 102.07, 86.87. m/z HRMS (EI) found $[M]^+$ 235.0996, $C_{16}H_{13}NO^+$ requires 235.0997.

tert-butyl phenyl(propa-1,2-dien-1-yl)carbamate

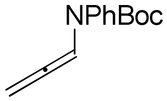

^{1}H NMR (400 MHz, $CDCl_3$) δ 7.42 – 7.35 (m, 2H), 7.33 – 7.27 (m, 2H), 7.25 – 7.19 (m, 2H), 5.07 (s, 1H), 5.05 (s, 1H), 1.48 (s, 9H). ^{13}C NMR (100 MHz, $CDCl_3$) δ 201.62, 152.47, 139.38, 128.62, 128.16, 127.21, 102.22, 86.51, 81.57, 28.30. m/z HRMS (EI) found $[M]^+$ 231.1258, $C_{14}H_{17}NO_2^+$ requires 231.1259.

N-benzyl-*N*-(propa-1,2-dien-1-yl)benzamide

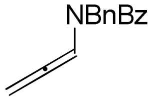

^{1}H NMR (400 MHz, $CDCl_3$) δ 8.16 – 6.31 (m, 11H), 5.41 – 5.10 (m, 2H), 5.04 – 4.59 (m, 2H). ^{13}C NMR (100 MHz, $CDCl_3$) δ 200.52, 137.53, 130.45, 128.56, 128.07, 127.23, 102.54, 87.39, 47.70. m/z HRMS (EI) found $[M]^+$ 249.1155, $C_{17}H_{15}NO^+$ requires 249.1154.

N-phenyl-*N*-(propa-1,2-dien-1-yl)isobutyramide

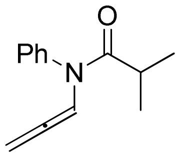

^{1}H NMR (400 MHz, $CDCl_3$) δ 7.67 (t, *J* = 6.6 Hz, 1H), 7.47 – 7.32 (m, 3H), 7.17 (d, *J* = 8.3 Hz, 2H), 4.95 (s, 1H), 4.94 (s, 1H), 2.45 (hept, *J* = 6.8 Hz, 1H), 1.04 (d, *J* = 6.7 Hz, 6H). ^{13}C NMR (100 MHz, $CDCl_3$) δ 203.00, 175.51, 139.79, 129.45, 128.67, 128.52, 101.32, 86.22, 31.84, 19.72. m/z HRMS (EI) found $[M]^+$ 201.1154, $C_{13}H_{15}NO^+$ requires 201.1154.

N-phenyl-*N*-(propa-1,2-dien-1-yl)pivalamide

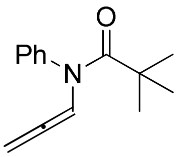

^{1}H NMR (400 MHz, $CDCl_3$) δ 7.68 (t, *J* = 6.4 Hz, 1H), 7.43 – 7.29 (m, 3H), 7.18 (dd, *J* = 8.0, 1.7 Hz, 2H), 4.88 (s, 1H), 4.86 (s, 1H), 1.06 (s, 9H). ^{13}C NMR (100 MHz, $CDCl_3$) δ 202.86, 175.99, 140.61, 130.29, 128.75, 128.49, 104.30, 86.98, 41.32, 29.41. m/z HRMS (EI) found $[M]^+$ 215.1312, $C_{14}H_{17}NO^+$ requires 215.1310.

Synthesis of the substrates:

Substrates **1a**, **1q**, **1t** and **1u** are commercial available. Other substrates were synthesized according literatures.[6]

Synthesis of products:

General procedure:

To the substrate **1** (0.30 mmol) in a 1 dram (15 x 45 mm) vial equipped with an 8 mm magnetic stirrer bar was added toluene (0.3 ml). Subsequently, allenamide (0.3 mmol), 5Å MS (90 mg), and **(*S*)-TRIP** (23 mg, 0.03 mmol or 46 mg, 0.06 mmol) were added. After all the reagents were dissolved, the mixture was warmed to 40 ºC. After 8h, another portion of allenamide (0.3 mmol) was added into the mixture. After heated at 40 ºC for another 20h or 40h, the mixture was cooled to rt and directly purified by flash column chromatography to afford the desired product.

The relevant racemic products were synthesized in the same procedure expect **(±)-TRIP** (10 mol%) was used as catalyst.

(*S,E*)-*N*-(3-(2-oxo-1-phenylcyclohexyl)prop-1-en-1-yl)-*N*-phenylbenzamide

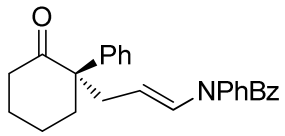

^{1}H NMR (400 MHz, $CDCl_3$) δ 7.40 – 7.15 (m, 12H), 7.03 (dd, *J* = 10.3, 7.5 Hz, 4H), 4.41 (dt, *J* = 14.8, 7.8 Hz, 1H), 2.63 (dd, *J* = 14.0, 2.8 Hz, 1H), 2.52 (dd, *J* = 14.1, 7.4 Hz, 1H), 2.45 – 2.24 (m, 3H), 2.02 – 1.88 (m, 1H), 1.82 – 1.58 (m, 4H). ^{13}C NMR (100 MHz, $CDCl_3$) δ 213.11, 168.72, 140.43, 140.18, 135.65, 131.76, 129.90, 129.29, 129.02, 128.82, 128.66, 127.84, 127.69, 126.86, 126.81, 111.89, 57.57, 41.22, 40.21, 35.22, 28.36, 21.59. m/z HRMS (ESI) found $[M+H]^+$ 410.2106, $C_{28}H_{28}O_2N^+$ requires 410.2115. HPLC (Chiralpak AD column, 85:15 hexanes/ isopropanol, 1 ml/min; tr = 15.5 min (minor), 13.5 min (major); 70% ee.

(*S,E*)-*N*-(3-(2-oxo-1-phenylcyclohexyl)prop-1-en-1-yl)-*N*-phenylacetamide

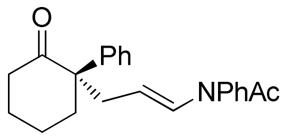

^{1}H NMR (400 MHz, $CDCl_3$) δ 7.46 – 7.31 (m, 3H), 7.26 – 7.19 (m, 3H), 7.15 (t, *J* = 7.3 Hz, 1H), 7.02 (d, *J* = 7.1 Hz, 2H), 6.94 (d, *J* = 7.4 Hz, 2H), 3.99 (dt, *J* = 14.3, 7.0 Hz, 1H), 2.52 (dd, *J* = 14.2, 2.9 Hz, 1H), 2.41 (dd, *J* = 14.0, 7.2 Hz, 1H), 2.36 – 2.17 (m, 3H), 1.93 – 1.84 (m, 1H), 1.77 (s, 3H), 1.70 – 1.51 (m, 4H). ^{13}C NMR (100 MHz, $CDCl_3$) δ 213.14, 168.31, 140.41, 140.06, 130.27, 129.88, 128.77, 128.72, 128.56, 126.79, 126.73, 110.21, 57.50, 41.05, 40.20, 35.12, 28.34, 23.25, 21.55. m/z HRMS (ESI) found $[M+Na]^+$ 370.1769, $C_{23}H_{25}O_2NNa^+$ requires 370.1778. HPLC (Chiralpak IB column, 97:3 hexanes/ isopropanol, 1 ml/min; tr = 13.9 min (minor), 14.6 min (major); 82% ee.

(*S,E*)-*N*-(3-(2-oxo-1-phenylcyclohexyl)prop-1-en-1-yl)-*N*-phenylisobutyramide

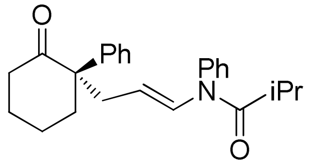

^{1}H NMR (400 MHz, $CDCl_3$) δ 7.47 – 7.32 (m, 3H), 7.31 – 7.18 (m, 3H), 7.15 (t, *J* = 7.3 Hz, 1H), 7.04 (d, *J* = 7.5 Hz, 2H), 6.93 (d, *J* = 7.6 Hz, 2H), 3.93 (dt, *J* = 14.7, 7.5 Hz, 1H), 2.53 (d, *J* = 13.2 Hz, 1H), 2.41

(dd, J = 14.1, 7.1 Hz, 1H), 2.35 – 2.17 (m, 4H), 1.88 (dd, J = 11.0, 5.4 Hz, 1H), 1.76 – 1.51 (m, 4H), 0.99 (d, J = 6.8 Hz, 6H). ^{13}C NMR (100 MHz, $CDCl_3$) δ 213.24, 175.18, 140.41, 139.64, 130.73, 129.81, 128.88, 128.70, 128.49, 126.76, 126.68, 110.60, 57.43, 41.09, 40.17, 35.06, 31.91, 28.33, 21.51, 19.62. m/z HRMS (ESI) found $[M+H]^+$ 376.2264, $C_{25}H_{30}O_2N^+$ requires 376.2271. HPLC (Chiralpak AD column, 90:10 hexanes/ isopropanol, 1 ml/min; tr = 7.4 min (minor), 8.1 min (major); 90% ee.

(*S,E*)-*N*-(3-(2-oxo-1-phenylcyclohexyl)prop-1-en-1-yl)-*N*-phenylpivalamide (**3a**)

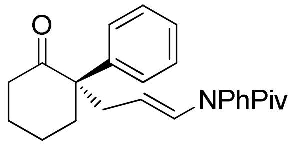

^{1}H NMR (400 MHz, $CDCl_3$) δ 7.43 – 7.28 (m, 3H), 7.24 – 7.11 (m, 4H), 7.04 (d, *J* = 6.6 Hz, 2H), 6.90 (d, *J* = 8.4 Hz, 2H), 3.83 (dt, *J* = 14.8, 7.8 Hz, 1H), 2.49 (dd, *J* = 14.2, 2.7 Hz, 1H), 2.39 (dd, *J* = 14.1, 7.1 Hz, 1H), 2.32 – 2.16 (m, 3H), 1.87 (ddd, *J* = 9.4, 6.1, 3.1 Hz, 1H), 1.71 – 1.45 (m, 4H), 1.01 (s, 9H). ^{13}C NMR (101 MHz, $CDCl_3$) δ 213.21, 175.89, 140.38, 140.34, 133.53, 130.32, 129.09, 128.68, 128.28, 126.79, 126.63, 110.03, 77.48, 77.16, 76.84, 57.49, 41.10, 40.94, 40.17, 35.02, 29.18, 28.34, 21.53. m/z HRMS (ESI) found $[M+H]^+$ 390.2425, $C_{26}H_{31}O_2N^+$ requires 390.2428.HPLC (Chiralpak AD column, 92:8 hexanes/ isopropanol, 1 ml/min; tr = 12.1 min (major), 13.5 min (minor); 94% ee.

(*S,E*)-*N*-(3-(2-oxo-1-(p-tolyl)cyclohexyl)prop-1-en-1-yl)-*N*-phenylpivalamide (**3b**)

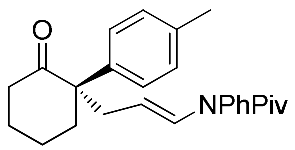

^{1}H NMR (400 MHz, $CDCl_3$) δ 7.43 – 7.30 (m, 3H), 7.16 (d, *J* = 14.3 Hz, 1H), 7.10 – 7.01 (m, 4H), 6.80 (d, *J* = 8.0 Hz, 2H), 3.87 (dt, *J* = 14.0, 7.8 Hz, 1H), 2.46 (dd, *J* = 14.4, 2.8 Hz, 1H), 2.40 – 2.33 (m, 1H), 2.32 – 2.23 (m, 5H), 2.23 – 2.15 (m, 1H), 1.92 – 1.82 (m, 1H), 1.68 – 1.43 (m, 4H), 1.03 (s, 9H). ^{13}C NMR (101 MHz, $CDCl_3$) δ 213.51, 175.99, 140.49, 137.46, 136.26, 133.50, 130.37, 129.44, 129.15, 128.27, 126.72, 110.28, 57.22, 41.19, 40.98, 40.16, 35.06, 29.21, 28.37, 21.59, 21.02. m/z HRMS (ESI) found $[M+H]^+$ 404.2575, $C_{27}H_{34}O_2N^+$ requires 404.2584. HPLC Chiralpak AD column, 96:4 hexanes/ isopropanol, 1 ml/min; tr = 10.9 min (minor), 13.5 min (major); 94% ee.

(*S,E*)-*N*-(3-(1-(naphthalen-2-yl)-2-oxocyclohexyl)prop-1-en-1-yl)-*N*-phenylpivalamide (**3c**)

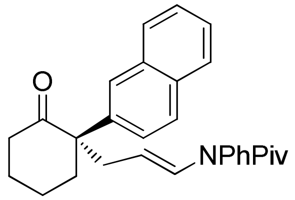

^{1}H NMR (400 MHz, $CDCl_3$) δ 7.84 – 7.71 (m, 3H), 7.54 – 7.43 (m, 2H), 7.38 (t, *J* = 1.3 Hz, 1H), 7.34 – 7.24 (m, 3H), 7.20 (d, *J* = 14.1 Hz, 1H), 7.06 (dd, *J* = 8.6, 1.9 Hz, 1H), 7.00 (d, *J* = 6.5 Hz, 2H). 3.86 (dt, *J* = 14.0, 7.8 Hz, 1H), 2.67 (dd, *J* = 14.4, 2.8 Hz, 1H), 2.55 (ddd, *J* = 14.3, 7.2, 1.4 Hz, 1H), 2.42 – 2.24 (m, 3H), 1.97 – 1.84 (m, 1H), 1.82 – 1.58 (m, 4H), 1.00 (s, 9H). ^{13}C NMR (101 MHz, $CDCl_3$) δ 213.31, 175.91, 140.34, 137.86, 133.59, 133.42, 132.20, 130.24, 129.05, 128.54, 128.24, 127.93, 127.56, 126.20, 126.00, 125.83, 124.82, 109.92, 57.70, 40.92, 40.38, 35.28, 29.13, 28.40, 21.67. m/z HRMS (ESI) found $[M+Na]^+$ 462.2392, $C_{30}H_{33}O_2NNa^+$ requires 462.2404. HPLC (Chiralpak IC column, 85:15 hexanes/ isopropanol, 1 ml/min; tr = 15.3 min (major), 20.1 min (minor); 94% ee.

(*S,E*)-*N*-(3-(1-(3-bromophenyl)-2-oxocyclohexyl)prop-1-en-1-yl)-*N*-phenylpivalamide (**3d**)

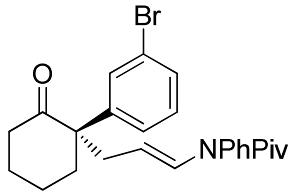

^{1}H NMR (400 MHz, $CDCl_3$) δ 7.44 – 7.36 (m, 2H), 7.34 (d, *J* = 7.2 Hz, 1H), 7.31 – 7.27 (m, 1H), 7.17 (d, *J* = 14.1 Hz, 1H), 7.13 – 7.01 (m, 4H), 6.82 (d, *J* = 7.8 Hz, 1H), 3.74 (dt, *J* = 14.6, 7.9 Hz, 1H), 2.52 – 2.33 (m, 2H), 2.30 – 2.14 (m, 3H), 1.98 – 1.83 (m, 1H), 1.74 – 1.43 (m, 4H), 1.01 (s, 9H). ^{13}C NMR (101 MHz, $CDCl_3$) δ 212.40, 175.86, 142.78, 140.16, 133.93, 130.42, 130.31, 129.87, 129.24, 128.45, 125.65, 122.83, 108.84, 57.46, 41.00, 40.87, 40.22, 35.04, 29.20, 28.26, 21.46. m/z HRMS (ESI) found $[M+H]^+$ 468.1526, $C_{26}H_{31}NO_2Br^+$ requires 468.1533. HPLC (Chiralpak AD column, 96:4 hexanes/ isopropanol, 1 ml/min; tr = 12.0 min (minor), 13.6 min (major); 94% ee.

(*S,E*)-*N*-(3-(2-oxo-1-(4-(trifluoromethyl)phenyl)cyclohexyl)prop-1-en-1-yl)-*N*-phenylpivalamide (**3e**)

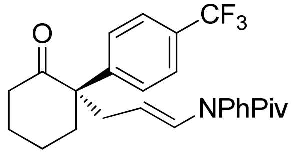

^{1}H NMR (400 MHz, $CDCl_3$) δ 7.47 (d, *J* = 7.9 Hz, 2H), 7.40 – 7.29 (m, 3H), 7.19 (d, *J* = 14.2 Hz, 1H), 7.07 – 6.97 (m, 4H), 3.69 (dt, *J* = 15.0, 7.8 Hz, 1H), 2.57 – 2.36 (m, 2H), 2.32 – 2.12 (m, 3H), 1.96 – 1.82 (m, 1H), 1.74 – 1.53 (m, 4H), 1.00 (s, 9H). ^{13}C NMR (101 MHz, $CDCl_3$) δ 212.19, 175.94, 144.61, 140.28, 134.00, 130.32, 129.16, 128.42, 127.30, 125.61 (q, *J* = 3.6 Hz), 109.13, 57.61, 41.00, 40.82, 40.29, 35.03, 29.16, 28.19, 21.49. m/z HRMS (ESI) found $[M+H]^+$ 458.2291, $C_{27}H_{31}O_2NF_3^+$ requires 458.2301. HPLC (Chiralpak AD column, 92:8 hexanes/ isopropanol, 1 ml/min; tr = 6.5 min (minor), 7.9 min (major); 94% ee.

(*S,E*)-tert-butyl 4-(2-oxo-1-(3-(*N*-phenylpivalamido)allyl)cyclohexyl)benzoate (**3f**)

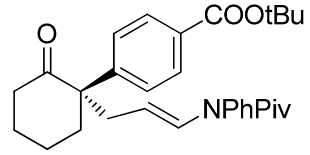

^{1}H NMR (400 MHz, $CDCl_3$) δ 7.85 (d, *J* = 8.4 Hz, 2H), 7.42 – 7.30 (m, 3H), 7.18 (d, *J* = 14.2 Hz, 1H), 7.04 (d, *J* = 6.7 Hz, 2H), 6.96 (d, *J* = 8.4 Hz, 2H), 3.78 (dt, *J* = 14.2, 7.9 Hz, 1H), 2.51 (dd, *J* = 11.9, 2.9 Hz, 1H), 2.42 (ddd, *J* = 14.3, 7.2, 1.4 Hz, 1H), 2.34 – 2.16 (m, 3H), 1.94 – 1.85 (m, 1H), 1.73 – 1.50 (m, 14H), 1.02 (s, 9H). ^{13}C NMR (101 MHz, $CDCl_3$) δ 212.52, 175.96, 165.48, 145.23, 140.33, 133.87, 130.50, 130.35, 129.83, 129.18, 128.39, 126.76, 109.47, 81.12, 57.80, 41.01, 40.92, 40.33, 35.10, 29.20, 28.30, 21.56. m/z HRMS (ESI) found $[M+H]^+$ 490.2941, $C_{31}H_{40}O_4N^+$ requires 490.2952. HPLC (Chiralpak AD column, 92:8 hexanes/ isopropanol, 1 ml/min; tr = 12.1 min (major), 13.5 min (minor); 94% ee.

(*S,E*)-*N*-(3-(1-(benzo[d][1,3]dioxol-5-yl)-2-oxocyclohexyl)prop-1-en-1-yl)-*N*-phenylpivalamide (**3g**)

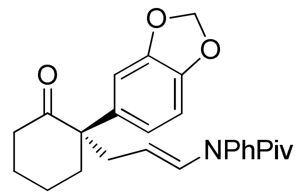

^{1}H NMR (400 MHz, $CDCl_3$) δ 7.43 – 7.29 (m, 3H), 7.18 (d, *J* = 14.2 Hz, 1H), 7.10 – 7.03 (m, 2H), 6.65 (d, *J* = 8.2 Hz, 1H), 6.41 (d, *J* = 1.9 Hz, 1H), 6.34 (dd, *J* = 8.1, 1.9 Hz, 1H), 5.91 (dd, *J* = 7.6, 1.6 Hz, 2H), 3.82 (dt, *J* = 14.0, 8.0 Hz, 1H), 2.41 – 2.34 (m, 2H), 2.29 (dt, *J* = 13.0, 6.4 Hz, 1H), 2.24 – 2.15 (m, 2H), 1.94 – 1.82 (m, 1H), 1.72 – 1.41 (m, 4H), 1.03 (s, 9H). ^{13}C NMR (101 MHz, $CDCl_3$) δ 213.15, 175.92, 148.00, 146.17, 140.39, 134.20, 133.54, 130.37, 129.15, 128.32,

120.08, 110.06, 108.41, 107.38, 101.08, 57.11, 41.12, 40.98, 40.03, 35.34, 29.21, 28.26, 21.49. HRMS (ESI) found $[M+Na]^+$ 456.2142, $C_{27}H_{31}O_4NNa^+$ requires 456.2145. HPLC (Chiralpak AD column, 92:8 hexanes/ isopropanol, 1 ml/min; tr = 12.2 min (minor), 15.3 min (major); 94% ee.

(*S,E*)-*N*-(3-(1-(3-methoxyphenyl)-2-oxocyclohexyl)prop-1-en-1-yl)-*N*-phenylpivalamide (**3h**)

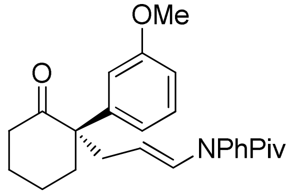

^{1}H NMR (400 MHz, $CDCl_3$) δ 7.42 – 7.28 (m, 3H), 7.16 – 7.08 (m, 2H), 7.04 (dd, *J* = 7.9, 1.7 Hz, 2H), 6.68 (dd, *J* = 8.2, 2.5 Hz, 1H), 6.53 – 6.41 (m, 2H), 3.82 (dt, *J* = 14.6, 7.8 Hz, 1H), 3.73 (s, 3H), 2.50 – 2.34 (m, 2H), 2.33 – 2.14 (m, 3H), 1.87 (ddd, *J* = 12.8, 5.6, 2.6 Hz, 1H), 1.76 – 1.44 (m, 4H), 1.01 (s, 9H). ^{13}C NMR (101 MHz, $CDCl_3$) δ 213.06, 175.86, 159.83, 141.95, 140.29, 133.44, 130.29, 129.65, 129.08, 128.23, 119.12, 113.20, 111.33, 109.86, 57.43, 55.18, 40.97, 40.92, 40.20, 35.07, 29.16, 28.22, 21.52. HRMS (ESI) found $[M+Na]^+$ 442.2355, $C_{27}H_{33}O_3N^+$ requires 442.2353. HPLC (Chiralpak IC column, 80:20 hexanes/ isopropanol, 1 ml/min; tr = 14.1 min (major), 15.4 min (minor); 96% ee.

(*S,E*)-*N*-(3-(2-oxo-1-(o-tolyl)cyclohexyl)prop-1-en-1-yl)-*N*-phenylpivalamide (**3i**)

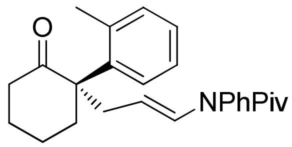

^{1}H NMR (400 MHz, $CDCl_3$) δ 7.44 – 7.28 (m, 3H), 7.20 (d, *J* = 14.2 Hz, 1H), 7.10 – 6.98 (m, 5H), 6.99 – 6.92 (m, 1H), 3.77 – 3.66 (m, 1H), 2.71 – 2.61 (m, 1H), 2.51 (dd, *J* = 14.3, 6.7 Hz, 1H), 2.43 – 2.28 (m, 2H), 2.28 – 2.18 (m, 1H), 2.03 (s, 3H), 1.99 – 1.89 (m, 1H), 1.78 – 1.64 (m, 2H), 1.63 – 1.55 (m, 1H), 1.42 – 1.33 (m, 1H), 1.02 (s, 9H). ^{13}C NMR (101 MHz, $CDCl_3$) δ 216.88, 175.94, 140.35, 138.16, 136.71, 133.40, 132.59, 130.43, 129.11, 128.31, 127.88, 126.78, 125.67, 109.96, 58.69, 41.02, 40.79, 39.09, 37.01, 30.73, 29.25, 21.53, 21.14. HRMS (ESI) found $[M+Na]^+$ 426.2400, $C_{27}H_{33}O_2NNa^+$ requires 426.2404. HPLC (Chiralpak IC column, 85:15 hexanes/ isopropanol, 1 ml/min; tr = 15.1 min (major), 17.1 min (minor); 87% ee.

(*R,E*)-*N*-(3-(2-oxo-1-(thiophen-2-yl)cyclohexyl)prop-1-en-1-yl)-*N*-phenylpivalamide (**3j**)

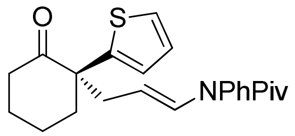

^{1}H NMR (400 MHz, $CDCl_3$) δ 7.41 – 7.29 (m, 3H), 7.24 (d, *J* = 16.0 Hz, 1H), 7.12 (d, *J* = 5.1 Hz, 1H), 7.09 – 7.04 (m, 2H), 6.85 (dd, *J* = 5.1, 3.5 Hz, 1H), 6.52 (d, *J* = 3.6 Hz, 1H), 3.94 (dt, *J* = 14.9, 7.7 Hz, 1H), 2.62 – 2.16 (m, 5H), 1.97 – 1.77 (m, 2H), 1.77 – 1.65 (m, 2H), 1.63 – 1.49 (m, 1H), 1.03 (s, 9H). ^{13}C NMR (101 MHz, $CDCl_3$) δ 210.85, 175.95, 145.85, 140.24, 133.80, 130.38, 129.12, 128.33, 126.89, 124.63, 124.26, 109.49, 55.26, 41.81, 40.98, 39.47, 37.55, 29.20, 27.34, 21.70. m/z HRMS (ESI) found $[M+Na]^+$ 418.1801, $C_{24}H_{29}O_2NSNa^+$ requires 418.1811. HPLC (Chiralpak IC column, 85:15 hexanes/ isopropanol, 1 ml/min; tr = 15.2 min (major), 16.7 min (minor); 96% ee.

(*S,E*)-*N*-(3-(2-oxo-1-phenylcyclopentyl)prop-1-en-1-yl)-N-phenylpivalamide (**3k**)

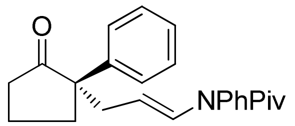

^{1}H NMR (400 MHz, $CDCl_3$) δ 7.40 – 7.28 (m, 4H), 7.22 (d, *J* = 4.3 Hz, 4H), 7.15 (dt, *J* = 8.5, 4.1 Hz, 1H), 7.09 – 7.02 (m, 2H), 3.90 (dt, *J* = 14.0, 7.7 Hz, 1H), 2.49 – 2.28 (m, 3H), 2.28 – 2.18 (m, 1H), 2.11 (dt, *J* = 18.9, 8.3 Hz, 1H), 1.96 (ddd, *J* = 13.3, 9.6, 6.6 Hz, 1H), 1.88 – 1.78 (m, 1H), 1.77 – 1.66 (m, 1H), 1.03 (s, 9H). ^{13}C NMR (101 MHz, $CDCl_3$) δ 219.22, 176.02, 140.22, 139.64, 133.97, 130.30, 129.15, 128.41, 128.39, 126.73, 126.69, 109.68, 57.01, 41.02, 39.90, 37.88, 33.42, 29.20, 18.55. m/z HRMS (ESI) found $[M+Na]^+$ 398.2082, $C_{25}H_{29}O_2NNa^+$ requires 398.2091. HPLC (Chiralpak AD column, 96:4 hexanes/ isopropanol, 1 ml/min; tr = 14.3 min (major), 16.1 min (minor); 89% ee.

(*S,E*)-*N*-(3-(4-oxo-3-phenyltetrahydro-2H-pyran-3-yl)prop-1-en-1-yl)-*N*-phenylpivalamide (**3l**)

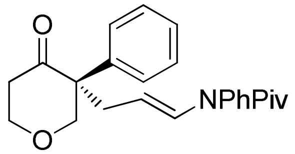

^{1}H NMR (400 MHz, $CDCl_3$) δ 7.39 – 7.29 (m, 3H), 7.29 – 7.21 (m, 3H), 7.21 – 7.15 (m, 1H), 7.11 (d, *J* = 7.2 Hz, 2H), 7.01 (d, *J* = 6.4 Hz, 2H), 4.53 (dd, *J* = 12.6, 1.8 Hz, 1H), 4.19 – 4.09 (m, 1H), 3.80 – 3.61 (m, 2H), 3.50 (d, *J* = 12.5 Hz, 1H), 2.66 – 2.46 (m, 2H), 2.39 – 2.27 (m, 1H), 2.20 – 2.09 (m, 1H), 1.02 (s,

9H). ^{13}C NMR (101 MHz, $CDCl_3$) δ 208.10, 176.04, 140.24, 138.81, 133.89, 130.34, 129.21, 128.84, 128.45, 127.15, 127.08, 108.60, 73.75, 69.19, 58.38, 41.08, 40.51, 36.08, 29.25. HRMS (ESI) found $[M+Na]^+$ 414.2037, $C_{25}H_{29}O_3NNa^+$ requires 414.2040. HPLC (Chiralpak IC column, 80:20 hexanes/ isopropanol, 1 ml/min; tr = 10.4 min (major), 12.6 min (minor); 90% ee.

N-((*E*)-3-((*S*)-2-oxo-1-((*E*)-styryl)cyclohexyl)prop-1-en-1-yl)-*N*-phenylpivalamide (**3m**)

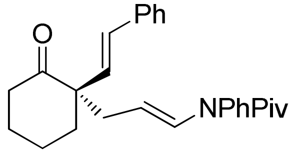

^{1}H NMR (400 MHz, $CDCl_3$) δ 7.44 – 7.15 (m, 9H), 7.10 (d, J = 7.1 Hz, 2H), 6.16 – 5.97 (m, 2H), 4.13 (dt, J = 14.8, 7.5 Hz, 1H), 2.61 – 2.12 (m, 4H), 1.97 – 1.84 (m, 2H), 1.78 – 1.67 (m, 2H), 1.66 – 1.54 (m, 2H), 1.03 (s, 9H). ^{13}C NMR (101 MHz, $CDCl_3$) δ 212.35, 175.99, 140.40, 136.92, 133.90, 133.03, 131.01, 130.34, 129.18, 128.55, 128.38, 127.61, 126.19, 109.95, 54.86, 41.05, 39.69, 38.78, 36.41, 29.24, 27.23, 21.67. m/z HRMS (ESI) found $[M+H]^+$ 416.2574, $C_{28}H_{34}O_2N^+$ requires 416.2584. HPLC (Chiralpak AD column, 92:8 hexanes/ isopropanol, 1 ml/min; tr = 8.3 min (minor), 9.0 min (major); 94% ee.

N-((*E*)-3-((*S*)-2-oxo-1-((*Z*)-styryl)cyclohexyl)prop-1-en-1-yl)-*N*-phenylpivalamide (**3n**)

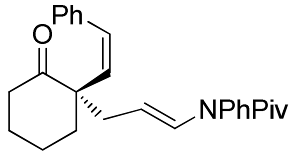

^{1}H NMR (400 MHz, $CDCl_3$) δ 7.47 – 7.33 (m, 4H), 7.22 – 7.12 (m, 5H), 7.01 – 6.93 (m, 2H), 6.54 (d, J = 12.5 Hz, 1H), 5.41 (d, J = 12.6 Hz, 1H), 4.26 (dt, J = 14.1, 7.8 Hz, 1H), 2.58 – 2.42 (m, 1H), 2.34 – 2.17 (m, 2H), 1.94 – 1.78 (m, 3H), 1.75 – 1.65 (m, 1H), 1.63 – 1.55 (m, 1H), 1.53 – 1.43 (m, 1H), 1.41 – 1.31 (m, 1H), 1.06 (s, 9H). ^{13}C NMR (101 MHz, $CDCl_3$) δ 213.60, 176.05, 140.35, 136.39, 135.46, 133.74, 131.90, 130.51, 129.27, 128.71, 128.48, 127.94, 127.43, 109.76, 55.44, 41.82, 41.14, 40.39, 38.34, 29.32, 29.05, 21.97. HRMS (ESI) found $[M+Na]^+$ 438.2405, $C_{28}H_{33}O_2NNa^+$ requires 438.2404. HPLC (Chiralpak AD column, 96:4 hexanes/ isopropanol, 1 ml/min; tr = 12.4 min (major), 16.9 min (minor); 91% ee.

(*S*,*E*)-*N*-(3-(2-oxo-1-vinylcyclohexyl)prop-1-en-1-yl)-*N*-phenylpivalamide (**3o**)

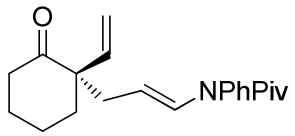

^{1}H NMR (400 MHz, $CDCl_3$) δ 7.46 – 7.32 (m, 4H), 7.16 – 7.10 (m, 2H), 5.68 (dd, *J* = 17.8, 10.8 Hz, 1H), 5.08 (d, *J* = 10.8 Hz, 1H), 4.81 (d, *J* = 17.8 Hz, 1H), 4.09 (dt, *J* = 15.0, 7.8 Hz, 1H), 2.50 – 2.36 (m, 1H), 2.32 – 2.14 (m, 3H), 1.93 – 1.75 (m, 2H), 1.73 – 1.48 (m, 4H), 1.05 (s, 9H). ^{13}C NMR (101 MHz, $CDCl_3$) δ 212.66, 176.08, 141.23, 140.38, 133.91, 130.45, 129.25, 128.50, 116.02, 109.66, 55.37, 41.16, 39.60, 38.31, 35.79, 29.33, 27.25, 21.50. HRMS (ESI) found $[M+H]^+$ 340.2271, $C_{22}H_{30}O_2N^+$ requires 340.2271. HPLC (Chiralpak OJ column, 95:5 hexanes/ isopropanol, 1 ml/min; tr = 13.3 min (major), 14.5 min (minor); 91% ee.

(*S,E*)-*N*-(3-(1-(2-methylprop-1-en-1-yl)-2-oxocyclohexyl)prop-1-en-1-yl)-*N*-phenylpivalamide (**3p**)

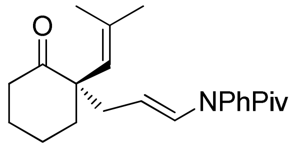

^{1}H NMR (400 MHz, $CDCl_3$) δ 7.46 – 7.27 (m, 4H), 7.13 (d, *J* = 6.9 Hz, 2H), 4.94 (s, 1H), 4.18 (dt, *J* = 14.9, 7.9 Hz, 1H), 2.56 (td, *J* = 13.0, 6.0 Hz, 1H), 2.40 (dd, *J* = 14.0, 7.4 Hz, 1H), 2.18 – 2.06 (m, 2H), 2.03 – 1.92 (m, 1H), 1.81 (d, *J* = 13.9 Hz, 1H), 1.70 (t, *J* = 13.2 Hz, 1H), 1.68 – 1.48 (m, 6H), 1.32 (s, 3H), 1.07 (s, 9H). ^{13}C NMR (101 MHz, $CDCl_3$) δ 215.46, 176.04, 140.65, 135.55, 133.30, 130.46, 129.18, 128.47, 128.32, 110.93, 54.31, 41.67, 41.09, 40.04, 37.37, 29.32, 29.19, 27.11, 21.90, 18.45. m/z HRMS (ESI) found $[M+H]^+$ 368.2577, $C_{24}H_{34}O_2N^+$ requires 368.2584. HPLC (Chiralpak IC column, 85:15 hexanes/ isopropanol, 1 ml/min; tr = 12.3 min (major), 13.4 min (minor); 88% ee.

(*S,E*)-*N*-(3-(2-oxo-[1,1'-bi(cyclohexan)]-1'-en-1-yl)prop-1-en-1-yl)-*N*-phenylpivalamide (**3q**)

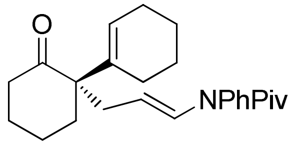

^{1}H NMR (400 MHz, $CDCl_3$) δ 7.46 – 7.34 (m, 3H), 7.30 (d, *J* = 14.3 Hz, 1H), 7.15 – 7.09 (m, 2H), 5.22 (t, *J* = 3.8 Hz, 1H), 4.01 (dt, *J* = 14.8, 7.8 Hz, 1H), 2.47 – 2.34 (m, 2H), 2.20 – 2.06 (m, 2H), 2.05 – 1.80 (m, 4H), 1.81 – 1.71 (m, 1H), 1.71 – 1.61 (m, 1H), 1.60 – 1.29 (m, 8H), 1.06 (s, 9H). ^{13}C NMR (101 MHz, $CDCl_3$) δ 214.51, 175.88, 140.57, 135.45, 132.75, 130.56, 129.11, 128.36, 124.78, 111.07, 58.46, 41.10,

40.36, 36.91, 35.03, 29.34, 28.19, 25.62, 25.27, 23.03, 22.29, 21.73. HRMS (ESI) found $[M+H]^+$ 394.2739, $C_{26}H_{36}O_2N^+$ requires 394.2741. HPLC (Chiralpak AD column, 96:4 hexanes/ isopropanol, 1 ml/min; tr = 10.5 min (minor), 11.5 min (major); 91% ee.

(*S,E*)-*N*-(3-(2-oxo-1-(phenylethynyl)cyclohexyl)prop-1-en-1-yl)-*N*-phenylpivalamide (**3r**)

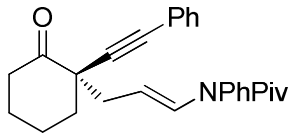

^{1}H NMR (400 MHz, $CDCl_3$) δ 7.55 (d, *J* = 14.4 Hz, 1H), 7.45 – 7.35 (m, 3H), 7.34 – 7.24 (m, 5H), 7.23 – 7.17 (m, 2H), 4.43 (dt, *J* = 14.5, 7.7Hz, 1H), 2.99 (td, *J* = 13.4, 5.8 Hz, 1H), 2.66 (ddd, *J* = 14.0, 6.6, 1.4 Hz, 1H), 2.36 – 2.27 (m, 1H), 2.26 – 2.17 (m, 1H), 2.15 – 2.02 (m, 3H), 1.82 – 1.71 (m, 1H), 1.70 – 1.58 (m, 1H), 1.50 (td, *J* = 13.2, 3.6 Hz, 1H), 1.11 (s, 9H). ^{13}C NMR (101 MHz, $CDCl_3$) δ 208.29, 176.04, 140.39, 134.04, 131.61, 130.51, 129.22, 128.33, 128.23, 128.18, 123.04, 109.62, 89.94, 86.42, 50.78, 41.11, 39.65, 39.23, 36.74, 29.29, 28.09, 22.41. m/z HRMS (ESI) found $[M+H]^+$ 414.2416, $C_{28}H_{32}O_2N^+$ requires 414.2428. HPLC (Chiralpak AD column, 92:8 hexanes/ isopropanol, 1 ml/min; tr = 6.7 min (minor), 8.2 min (major); 93% ee.

(*S,E*)-*N*-(3-(1-(hex-1-yn-1-yl)-2-oxocyclohexyl)prop-1-en-1-yl)-*N*-phenylpivalamide (**3s**)

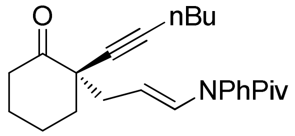

^{1}H NMR (400 MHz, $CDCl_3$) δ 7.51 – 7.30 (m, 4H), 7.16 (d, *J* = 7.5 Hz, 2H), 4.30 (dt, *J* = 14.7, 7.6 Hz, 1H), 2.86 (td, *J* = 13.3, 5.7 Hz, 1H), 2.45 (dd, *J* = 14.0, 6.5 Hz, 1H), 2.17 (d, *J* = 13.2 Hz, 1H), 2.12 – 2.02 (m, 3H), 2.01 – 1.83 (m, 3H), 1.70 – 1.60 (m, 1H), 1.58 – 1.45 (m, 1H), 1.39 – 1.21 (m, 5H), 1.06 (s, 9H), 0.86 (t, *J* = 7.0 Hz, 3H). ^{13}C NMR (101 MHz, $CDCl_3$) δ 209.20, 176.00, 140.47, 133.71, 130.50, 129.16, 128.32, 110.03, 86.46, 80.48, 49.94, 41.10, 39.47, 38.90, 37.04, 30.96, 29.31, 28.01, 22.26, 21.96, 18.44, 13.69. m/z HRMS (ESI) found $[M+H]^+$ 394.2733, $C_{26}H_{36}O_2N^+$ requires 394.2741. HPLC (Chiralpak AD column, 96:4 hexanes/ isopropanol, 1 ml/min; tr = 7.0 min (minor), 7.6 min (major); 91% ee.

(*S,E*)-*N*-(3-(1-methyl-2-oxocyclohexyl)prop-1-en-1-yl)-*N*-phenylpivalamide (**3t**)

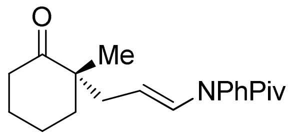

^{1}H NMR (400 MHz, $CDCl_3$) δ 7.47 – 7.34 (m, 4H), 7.19 – 7.11 (m, 2H), 4.09 (dt, *J* = 14.7, 7.8 Hz, 1H), 2.35 – 2.21 (m, 3H), 2.11 (dd, *J* = 13.9, 7.5 Hz, 1H), 1.84 – 1.55 (m, 5H), 1.49 – 1.37 (m, 1H), 1.04 (s, 9H), 0.88 (s, 3H). ^{13}C NMR (101 MHz, $CDCl_3$) δ 215.41, 176.10, 140.23, 133.87, 130.50, 129.31, 128.59, 109.05, 48.98, 41.21, 38.94, 38.62, 38.33, 29.35, 27.45, 22.69, 21.14. HRMS (ESI) found $[M+Na]^+$ 350.2088, $C_{21}H_{29}O_2NNa^+$ requires 350.2091. HPLC (Chiralpak AD column, 94:4 hexanes/ isopropanol, 0.5 ml/min; tr = 18.2 min (minor), 18.7 min (major); 70% ee.

(*S,E*)-*N*-(3-(1-butyl-2-oxocyclohexyl)prop-1-en-1-yl)-*N*-phenylpivalamide (**3u**)

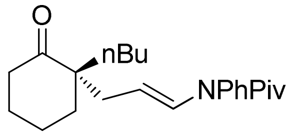

^{1}H NMR (400 MHz, $CDCl_3$) δ 7.46 – 7.33 (m, 4H), 7.17 – 7.12 (m, 2H), 4.07 (dt, *J* = 14.2, 7.8 Hz, 1H), 2.36 – 2.18 (m, 3H), 2.10 (ddd, *J* = 14.3, 7.3, 1.4 Hz, 1H), 1.89 – 1.78 (m, 1H), 1.66 – 1.44 (m, 5H), 1.27 – 1.12 (m, 4H), 1.06 (s, 9H), 0.94 – 0.83 (m, 2H), 0.80 (t, *J* = 7.2 Hz, 3H). ^{13}C NMR (101 MHz, $CDCl_3$) δ 215.16, 176.08, 140.40, 133.67, 130.47, 129.29, 128.51, 109.74, 51.93, 41.16, 39.38, 36.64, 35.43, 34.48, 29.34, 27.26, 25.53, 23.34, 20.93, 14.00. HRMS (ESI) found $[M+Na]^+$ 392.2556, $C_{24}H_{35}O_2NNa^+$ requires 392.2560. HPLC (Chiralpak IA column, 96:4 hexanes/ isopropanol, 1 ml/min; tr = 9.0 min (minor), 9.8 min (major); 78% ee.

Determination of the absolute stereochemistry of the products:

(*S*)-2-(1-(benzo[d][1,3]dioxol-5-yl)-2-oxocyclohexyl)acetaldehyde (**7g**)

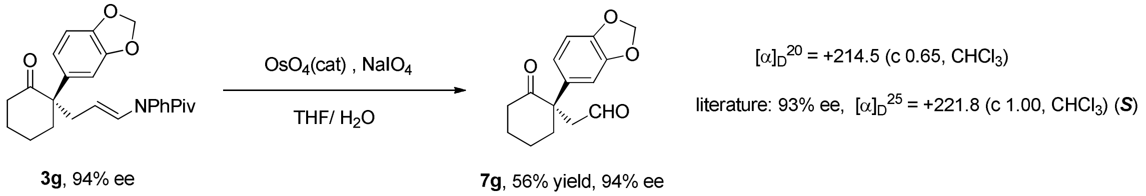

To a solution of **3g** (65 mg, 0.15 mmol) in THF / H_2O (2 mL / 2 mL) was added OsO_4 (48 uL, 4% in water, 0.0075 mmol) and $NaIO_4$ (112 mg, 0.525 mmol) at rt. After stirring overnight, the

mixture was quenched with satd. Na_2SO_3 solution and then extracted with Et_2O for 2 times. The combined organic layer was then washed with brine, dried over Na_2SO_4, filtered and concentrated *in vacuo* to give a residue, which was purified by flash chromatography (8:1 Hexane / EA) to afford the titled product **7g** (22.0 mg, 56% yield). ^{1}H NMR (400 MHz, $CDCl_3$) δ 9.57 (t, *J* = 2.5 Hz, 1H), 6.80 (d, *J* = 8.1 Hz, 1H), 6.71 (d, *J* = 2.0 Hz, 1H), 6.63 (dd, *J* = 8.1, 1.9 Hz, 1H), 6.00 – 5.94 (m, 2H), 2.70 – 2.58 (m, 3H), 2.51 – 2.41 (m, 1H), 2.40 – 2.33 (m, 1H), 2.03 – 1.89 (m, 2H), 1.80 – 1.67 (m, 3H). $[\alpha]_D^{20}$ = +214.5 (c 0.65, $CHCl_3$). The absolute configuration of the stereocenter was determined to be (*S*) by comparison of the optical rotation of **7g** with literature.[7] HPLC (Chiralpak IC column, 85:15 hexanes/ isopropanol, 1 ml/min; tr = 18.4 min (major), 21.1 min (minor); 94% ee.

Transformations of the product:

(*S*)-3-(2-oxo-1-phenylcyclohexyl)propanal (**4a**)

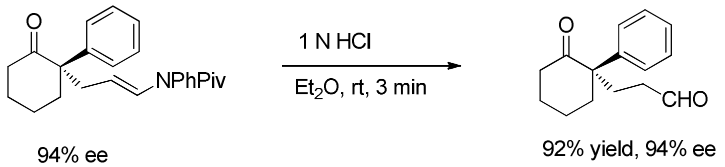

To a solution of **3a** (105 mg, 0.27 mmol) in Et_2O (5 mL) was added 2 N HCl solution (5 mL) at rt. After 3 min, the mixture was quenched with satd. $NaHCO_3$ solution and extracted with Et_2O for 3 times. The combined organic layer was then washed with brine, dried over Na_2SO_4, filtered and concentrated *in vacuo* to give a residue, which was purified by flash chromatography (15:1 Hexane / EA) to afford the titled product **4a** (57 mg, 92% yield). ^{1}H NMR (400 MHz, $CDCl_3$) δ 9.51 (s, 1H), 7.34 (t, *J* = 7.6 Hz, 2H), 7.24 (t, *J* = 7.3 Hz, 1H), 7.12 (d, *J* = 7.7 Hz, 2H), 2.66 (dd, *J* = 11.3, 3.4 Hz, 1H), 2.38 (td, *J* = 13.4, 6.3 Hz, 1H), 2.31 – 2.08 (m, 4H), 1.98 – 1.88 (m, 1H), 1.87 – 1.78 (m, 1H), 1.77 – 1.57 (m, 4H). ^{13}C NMR (101 MHz, $CDCl_3$) δ 213.34, 202.27, 139.97, 129.17, 127.21, 127.04, 56.79, 40.22, 39.28, 35.97, 32.55, 28.44, 21.64. HRMS (EI) found $[M]^+$ 230.1306, $C_{15}H_{18}O_2^+$ requires 230.1307. HPLC (Chiralpak IC column, 85:15 hexanes/ isopropanol, 1 ml/min; tr = 13.4 min (major), 16.8 min (minor); 94% ee.

(*1R,5S*)-5-phenylbicyclo[3.3.1]nonane-2,9-dione (**5a**)

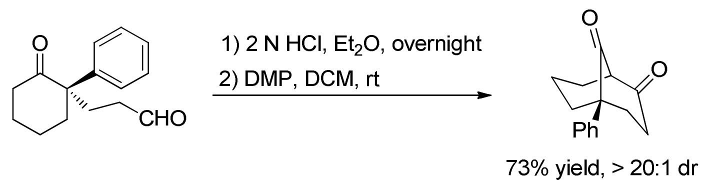

4a (18 mg, 0.078 mmol) was dissolved in 2 N HCl solution (3 mL) at rt. After stirring overnight, the solvent was removed *in vacuo* to give a residue, which was redissovled in DCM (2 mL) and followed by addition of DMP (66 mg, 0.15 mml) at rt. After stirring overnight, the mixture was quenched with satd. $NaHCO_3$ solution and extracted with DCM for 3 times. The combined organic layer was then washed with brine, dried over Na_2SO_4, filtered and concentrated *in vacuo* to give a residue, which was purified by flash chromatography (10:1 Hexane / EA) to afford the titled product **5a** (13.0 mg, 73% yield). ^{1}H NMR (400 MHz, $CDCl_3$) δ 7.39 (dd, *J* = 8.3, 6.8 Hz, 2H), 7.33 – 7.27 (m, 3H), 3.34 (dd, *J* = 4.6, 3.0 Hz, 1H), 2.85 (ddd, *J* = 16.7, 7.1, 5.2 Hz, 1H), 2.65 (dt, *J* = 16.7, 9.1 Hz, 1H), 2.56 – 2.26 (m, 5H), 2.20 – 2.08 (m, 1H), 1.94 – 1.83 (m, 2H). ^{13}C NMR (101 MHz, $CDCl_3$) δ 210.23, 210.05, 143.50, 128.33, 127.25, 127.17, 64.09, 53.60, 41.42, 40.90, 35.27, 32.12, 20.00. HRMS (EI) found $[M]^+$ 228.1155, $C_{15}H_{16}O_2^+$ requires 228.1150.

(4a*S*,8aS)-4a-phenyloctahydro-2H-chromen-2-one (**6a**)

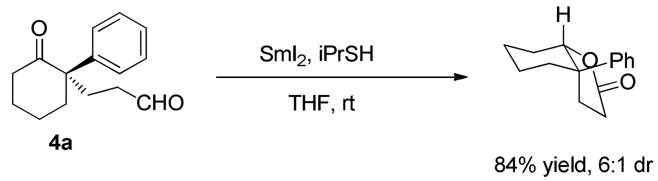

To a solution of SmI_2 (652 uL, 0.1 N in THF, 0.65 mmol) was added iPrSH (4.8 uL, 0.052 mmol) under Ar at rt. Then a solution of **4a** (30 mg, 0.13 mmol) in THF (1 mL) was slowly added into the above mixture. After stirring for 1 h, the mixture was quenched by filtering through a pad of silica gel. Concentration of the filtrate *in vacuo* gave a residue, which was purified by flash chromatography (5:1 Hexane/ EA) to afford the titled product **6a** (21.6 mg, 72% yield) with the minor isomer (3.7 mg, 12 % yield). Data for the major diastereoisomer: ^{1}H NMR (400 MHz, $CDCl_3$) δ 7.50 (d, *J* = 7.7 Hz, 2H), 7.33 (t, *J* = 7.5 Hz, 2H), 7.22 (t, *J* = 7.3 Hz, 1H), 4.38 (dd, *J* = 12.7, 4.3 Hz, 1H), 2.64 – 2.39 (m, 2H), 2.32 – 2.16 (m, 1H), 2.16 – 1.87 (m, 4H), 1.86 – 1.73 (m, 1H), 1.51 – 1.32 (m, 3H), 1.10 – 0.98 (m, 1H). ^{13}C NMR (101 MHz, $CDCl_3$) δ 171.28, 140.33, 128.88, 128.34, 126.64,

86.95, 42.30, 38.88, 37.65, 28.31, 27.78, 25.24, 21.52. HRMS (EI) found $[M]^+$ 230.1309, $C_{15}H_{18}O_2^+$ requires 230.1307.

(*S*)-2-(2-oxo-1-phenylcyclohexyl)acetaldehyde (**7a**)

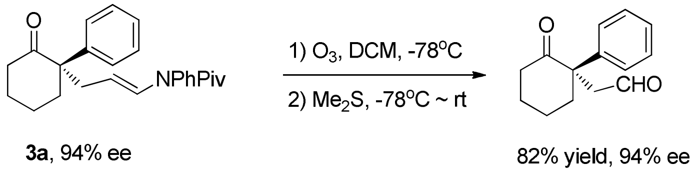

To a solution of **3a** (82 mg, 0.21 mmol) in DCM (10 mL) was bubbled with O_3 until the solution turned blue at -78 °C. To the above blue solution was added Me_2S (3 mL) and the mixture was slowly warmed to rt. After stirring overnight, the mixture was concentrated *in vacuo* to give a residue, which was purified by flash chromatography (DCM) to afford the titled product **7a** (37.2 mg, 82% yield). ^{1}H NMR (400 MHz, $CDCl_3$) δ 9.57 (t, *J* = 2.5 Hz, 1H), 7.39 (t, *J* = 7.6 Hz, 2H), 7.33 – 7.25 (m, 1H), 7.21 (d, *J* = 7.7 Hz, 2H), 2.76 (dd, *J* = 14.4, 3.3 Hz, 1H), 2.72 – 2.62 (m, 2H), 2.48 – 2.36 (m, 2H), 2.06 – 1.91 (m, 2H), 1.83 – 1.68 (m, 3H). ^{13}C NMR (101 MHz, $CDCl_3$) δ 212.11, 201.66, 139.73, 129.51, 127.59, 126.68, 56.37, 53.48, 39.80, 35.91, 28.02, 21.46. HRMS (EI) found $[M]^+$ 216.1146, $C_{14}H_{16}O_2^+$ requires 216.1150. HPLC (Chiralpak IC column, 90:10 hexanes/ isopropanol, 1 ml/min; tr = 15.1 min (minor), 19.5 min (major); 94% ee.

(3a*S*,7a*S*)-3a-phenyloctahydro-1H-indole (**8a**)

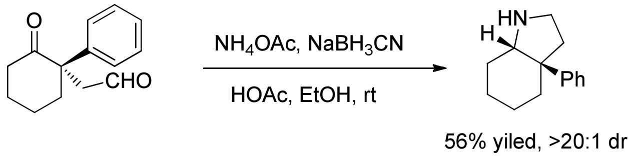

To a solution of **7a** (34 mg, 0.157 mmol) in EtOH (3 mL) was added NH_4OAc (24 mg, 0.315 mmol), $NaBH_3CN$ (40 mg, 0.628 mmol) and HOAc (9 uL, 0.157 mmol) at rt. After stirring for 20 h, the mixture was quenched with 2 N NaOH solution and extracted with DCM for 5 times. The combined organic layer was dried over Na_2SO_4, filtered and concentrated *in vacuo* to give a residue, which was purified by flash chromatography (50: 1: 1 DCM/ MeOH/ Et_3N) to afford the titled product **8a** (17.8 mg, 56% yield). ^{1}H NMR (400 MHz, $CDCl_3$) δ 7.41 – 7.29 (m, 4H), 7.24 – 7.17 (m, 1H), 4.41 (brs, 1H), 3.67 (t, *J* = 4.1 Hz, 1H, 3.31 (dt, *J* = 11.5, 8.2 Hz, 1H), 3.13 (td, *J* = 10.6, 4.4 Hz, 1H), 2.10 (ddd, *J* = 13.1, 8.7, 4.4 Hz, 1H), 2.03 – 1.64 (m, 6H), 1.55 – 1.45 (m, 2H), 1.29 – 1.17 (m,

1H). ^{13}C NMR (101 MHz, $CDCl_3$) δ 145.80, 128.51, 126.65, 126.12, 61.00, 47.99, 42.84, 41.00, 33.35, 25.79, 22.03, 20.89. HRMS (ESI) found $[M+H]^+$ 202.1588, $C_{14}H_{20}N^+$ requires 202.1590.

References:

[1] J. Seayad, A. M. Seayad, B. List, *J. Am. Chem. Soc.* 2006, **128**, 1086-1087.

[2] W. Xie, G. Jiang, H. Liu, J. Hu, X. Pan, H. Zhang, X. Wan, Y. Lai, D. Ma, *Angew. Chem. Int. Ed.* 2013, **52**, 12924-12927.

[3] V. Rauniyar, A. D. Lackner, G. L. Hamilton, F. D. Toste, *Science* 2011, **334**, 1681-1684.

[4] W. Kashikura, J. Itoh, K. Mori, T. Akiyama, *Chem. Asian J.* 2010, **5**, 470-472.

[5] V. Rauniyar, Z. J. Wang, H. E. Burks, F. D. Toste, *J. Am. Chem. Soc.* 2011, **133**, 8486-8489.

[6] a) X. Yang, R. J. Phipps, F. D. Toste, *J. Am. Chem. Soc.* 2014, **136**, 5225-5228; b) X. Yang, F. D. Toste, *J. Am. Chem. Soc.* 2015, **137**, 3205-3208.

[7] T. Kano, Y. Hayashi, K. Maruoka, *J. Am. Chem. Soc.* 2013, **135**, 7134-7137.

(*S,E*)-*N*-(3-(2-oxo-1-phenylcyclohexyl)prop-1-en-1-yl)-*N*-phenylbenzamide

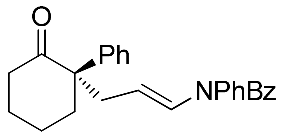

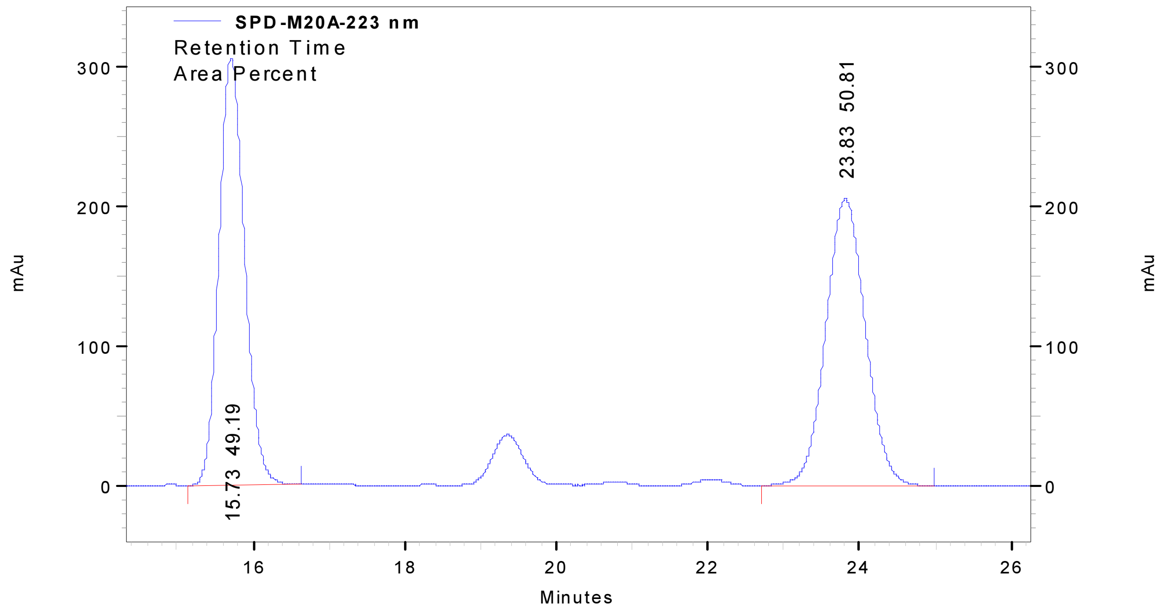

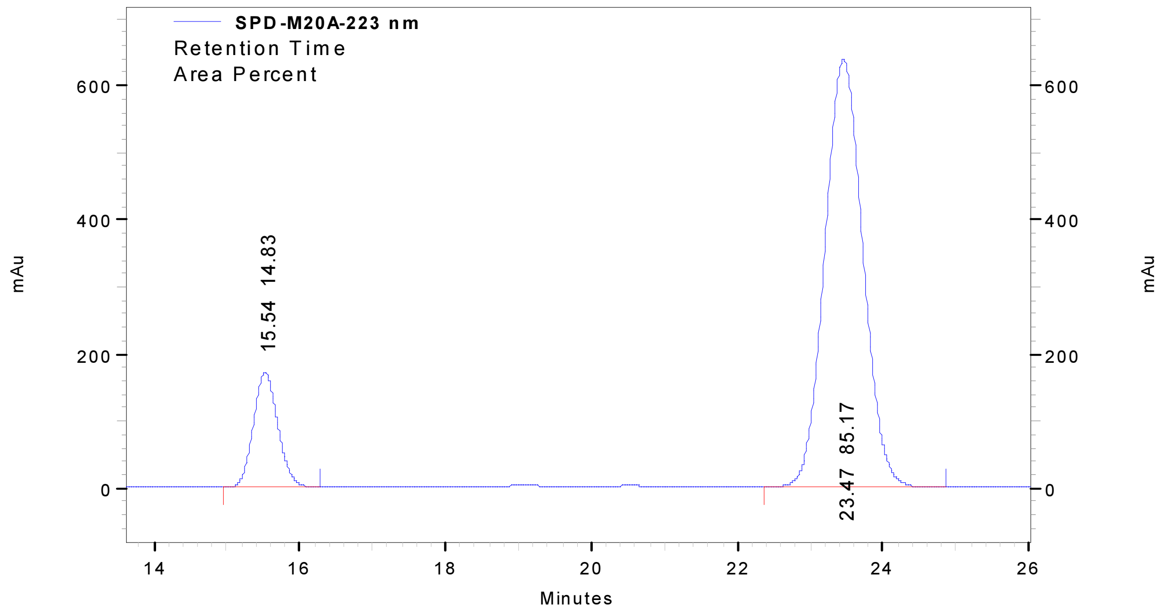

(*S,E*)-*N*-(3-(2-oxo-1-phenylcyclohexyl)prop-1-en-1-yl)-*N*-phenylacetamide

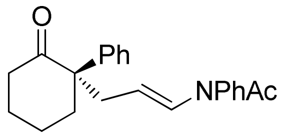

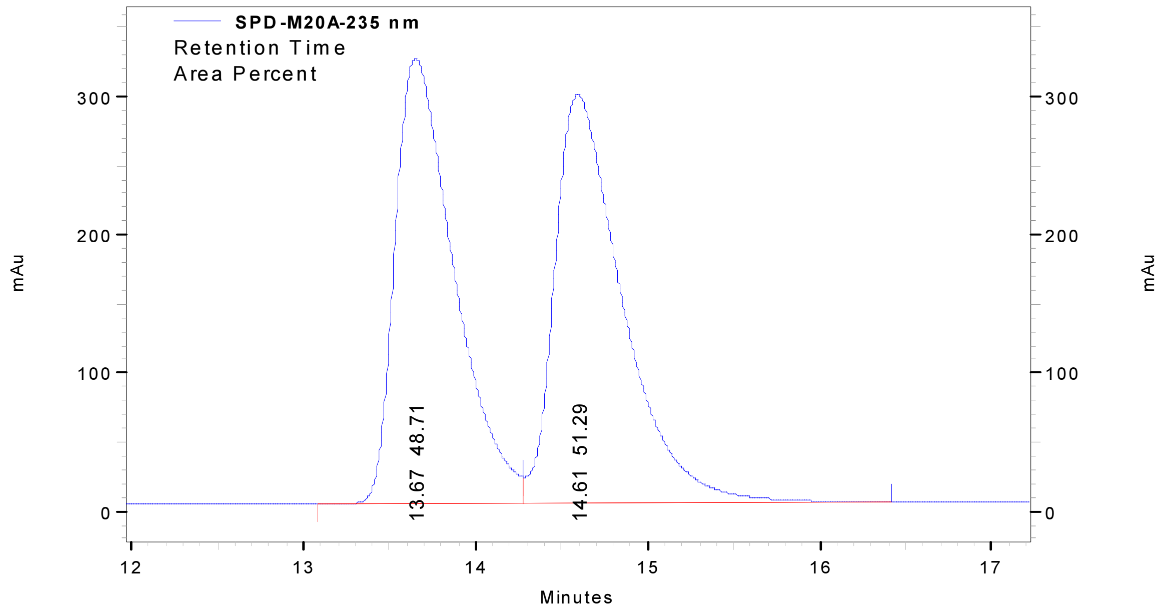

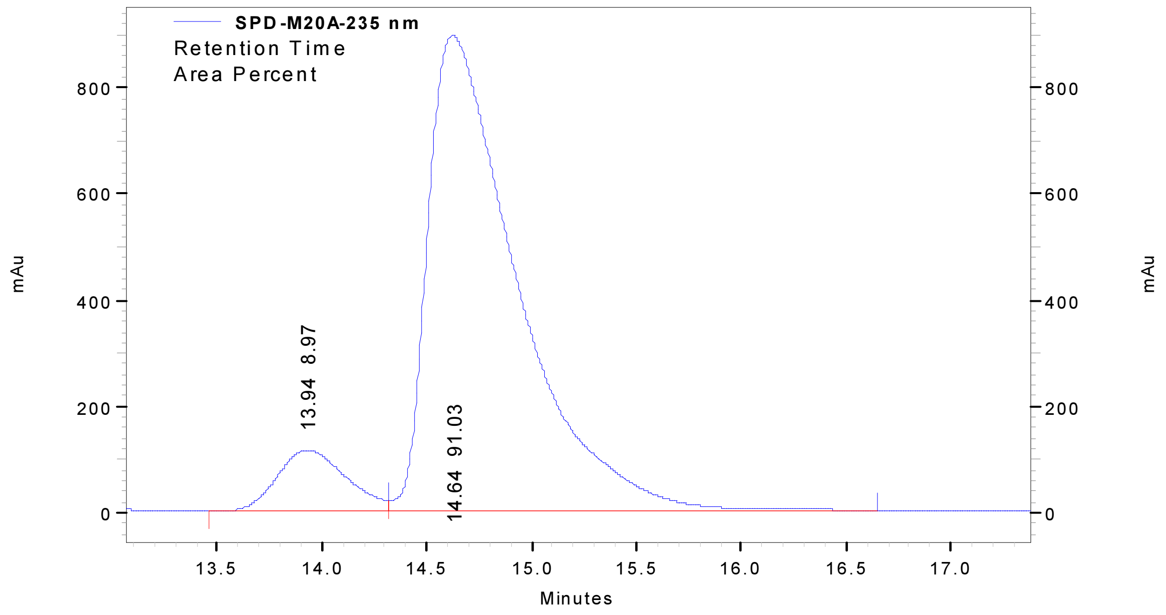

(*S,E*)-*N*-(3-(2-oxo-1-phenylcyclohexyl)prop-1-en-1-yl)-*N*-phenylisobutyramide

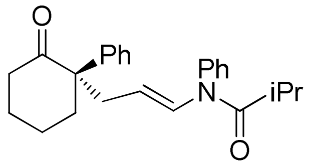

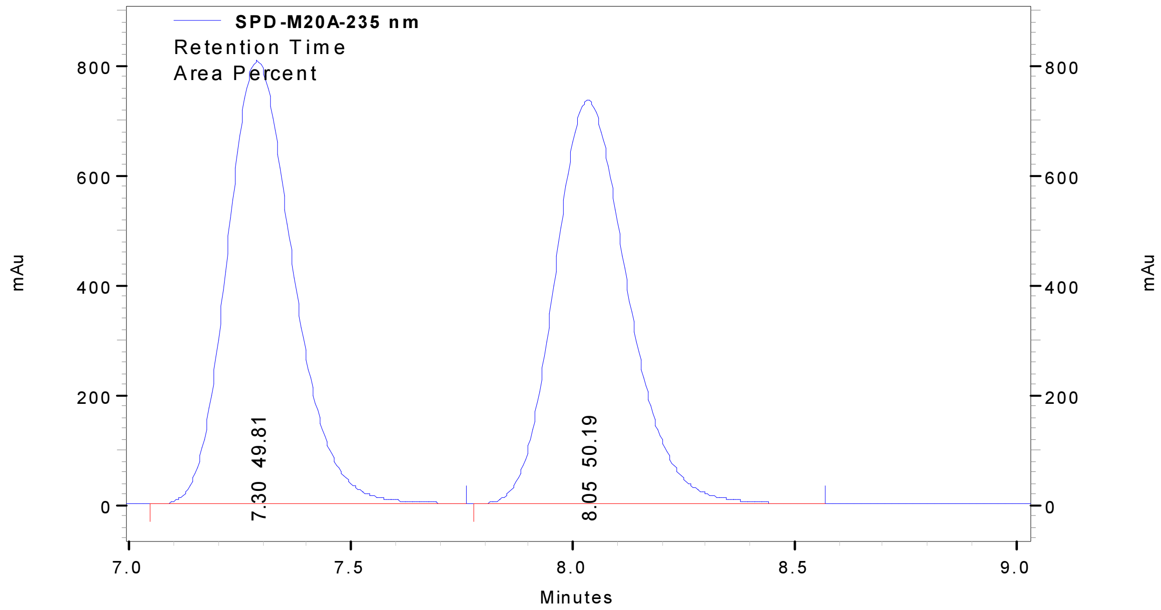

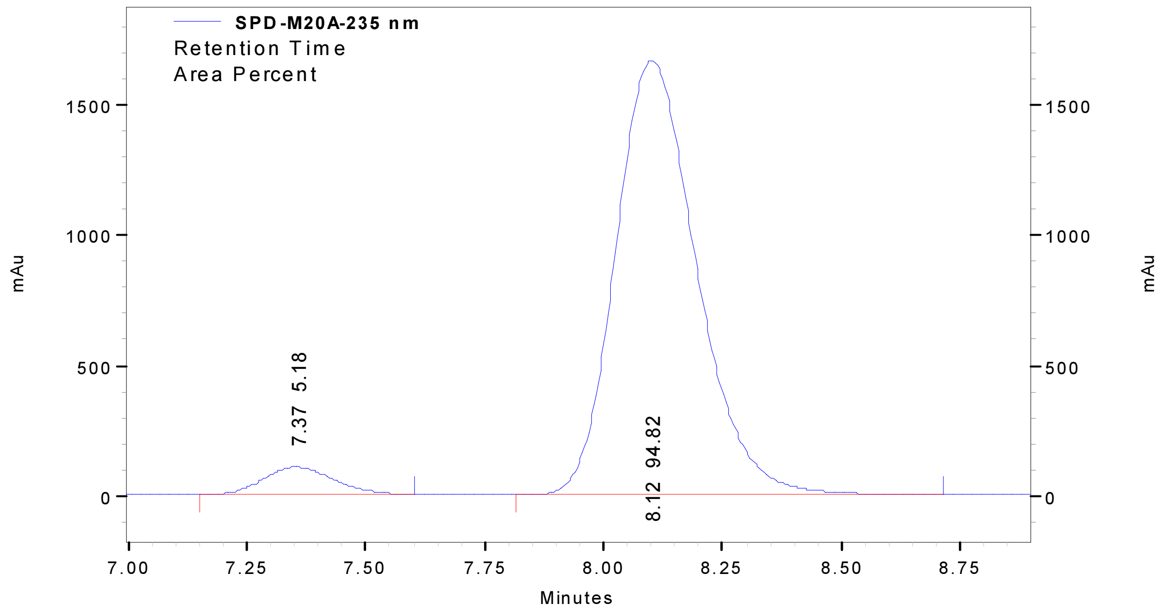

(*S,E*)-*N*-(3-(2-oxo-1-phenylcyclohexyl)prop-1-en-1-yl)-*N*-phenylpivalamide (**3a**)

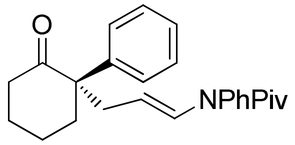

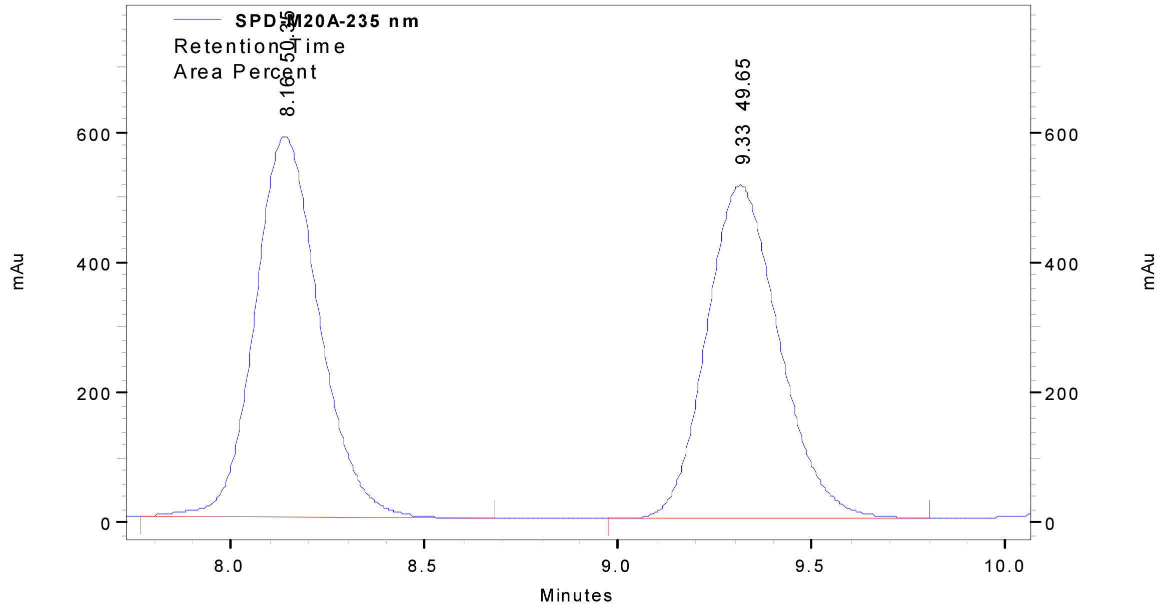

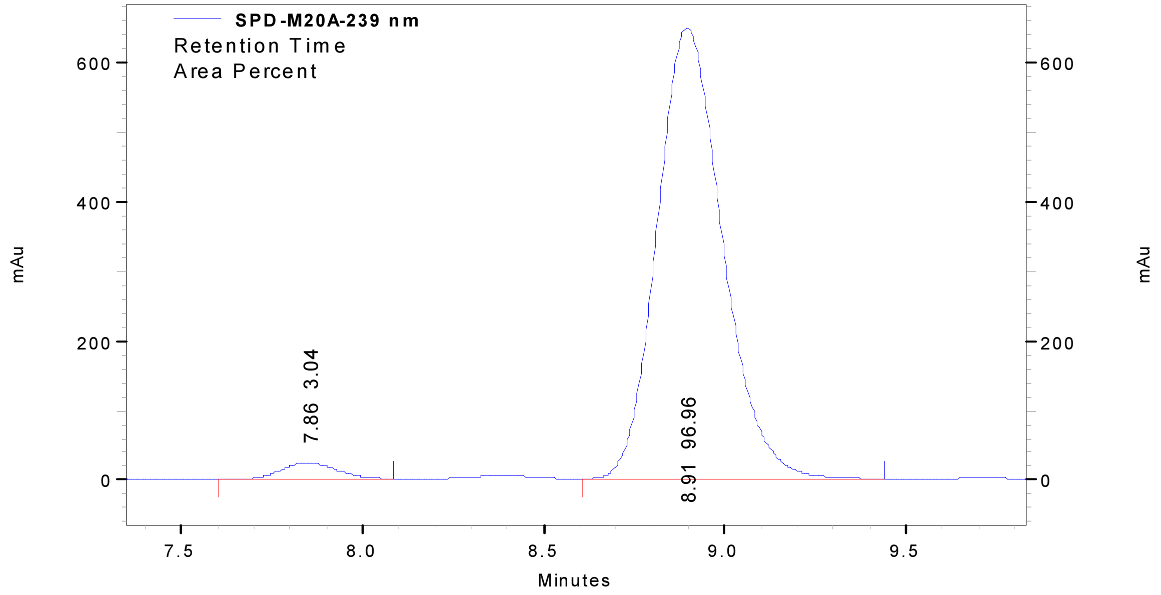

(*S*,*E*)-*N*-(3-(2-oxo-1-(p-tolyl)cyclohexyl)prop-1-en-1-yl)-*N*-phenylpivalamide (**3b**)

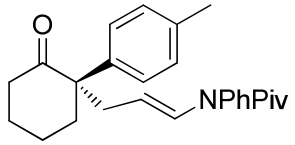

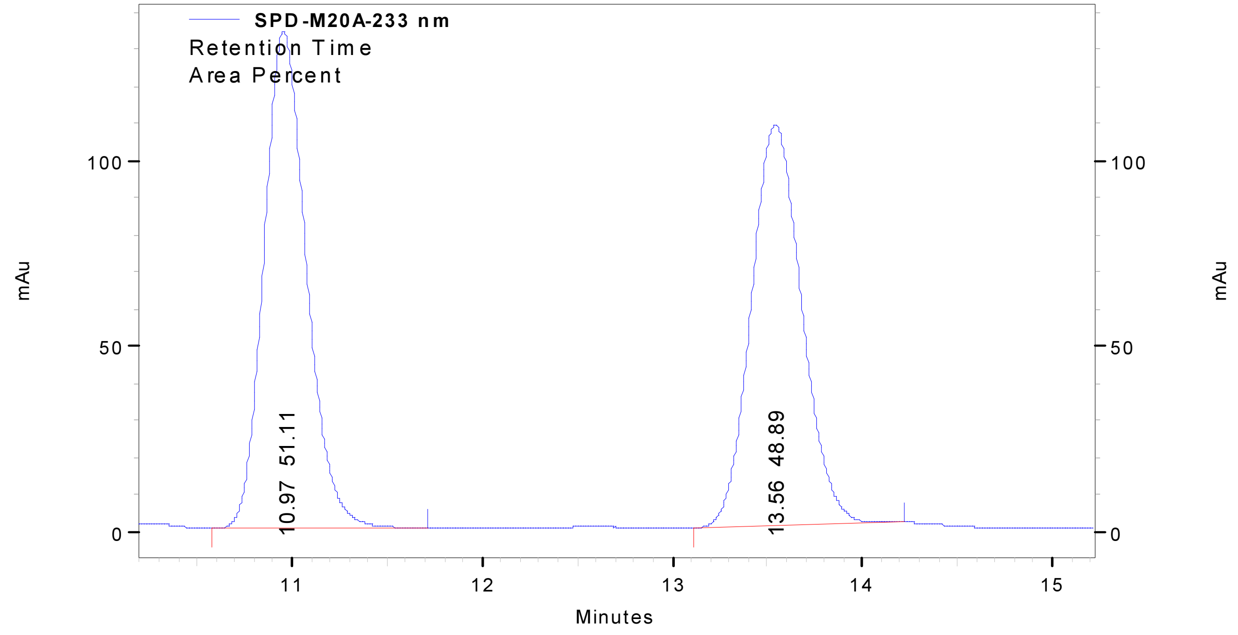

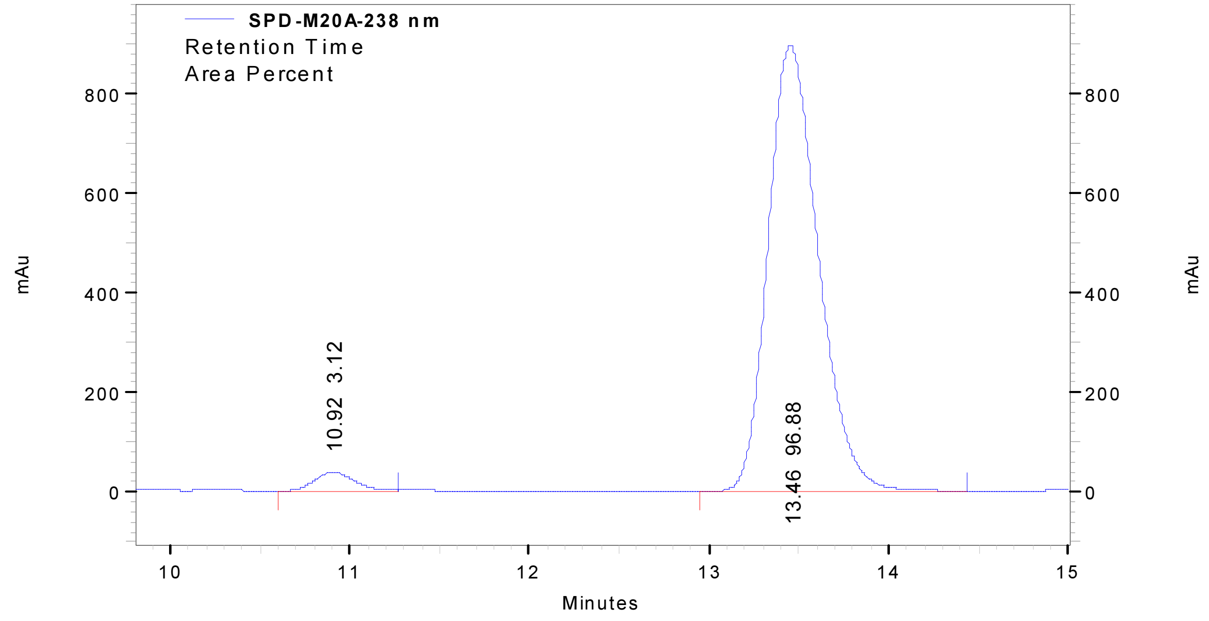

(*S,E*)-*N*-(3-(1-(naphthalen-2-yl)-2-oxocyclohexyl)prop-1-en-1-yl)-*N*-phenylpivalamide (**3c**)

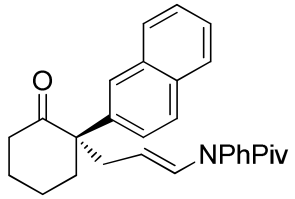

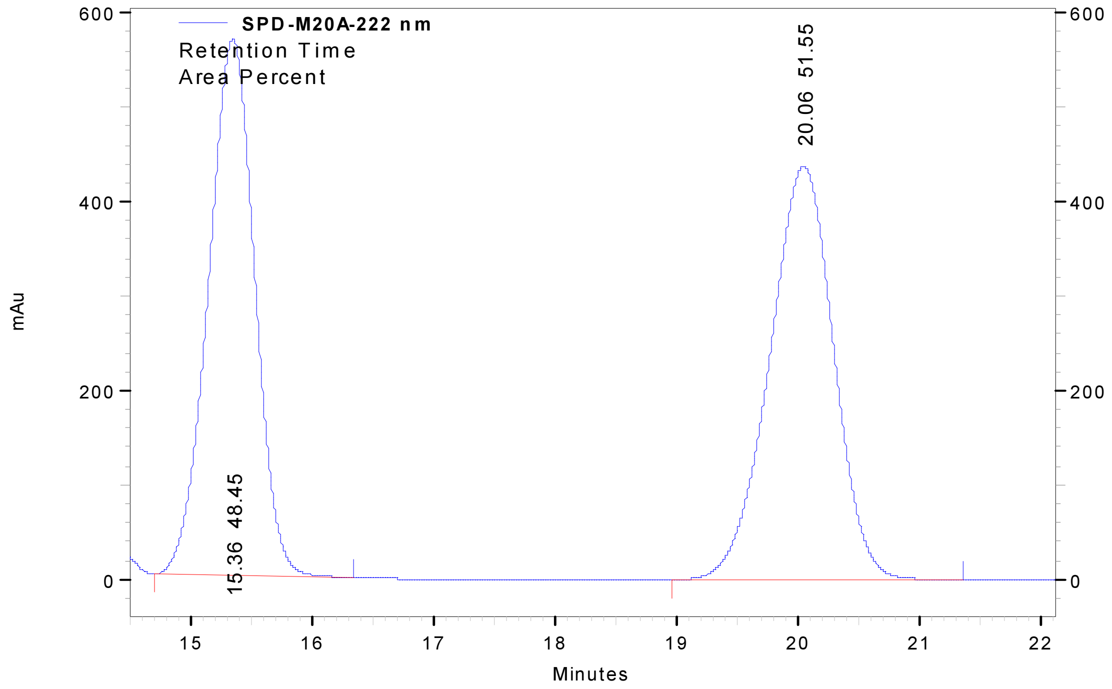

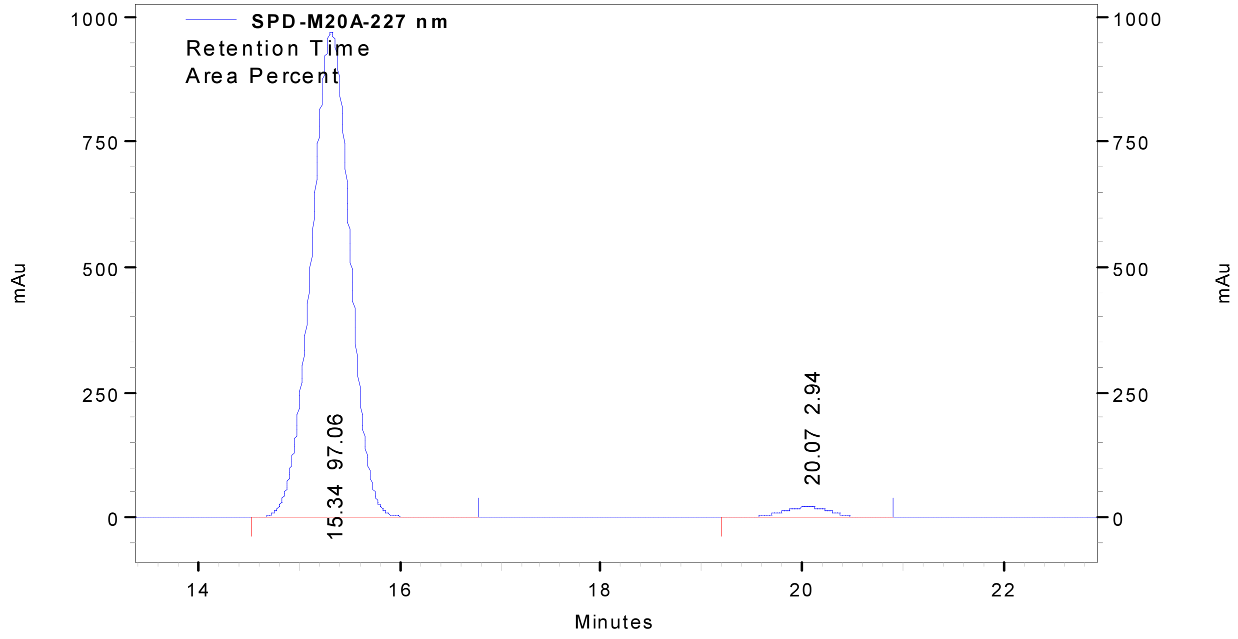

(*S*,*E*)-*N*-(3-(1-(3-bromophenyl)-2-oxocyclohexyl)prop-1-en-1-yl)-*N*-phenylpivalamide (**3d**)

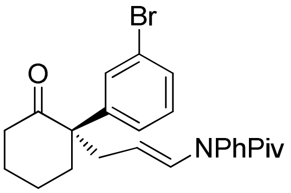

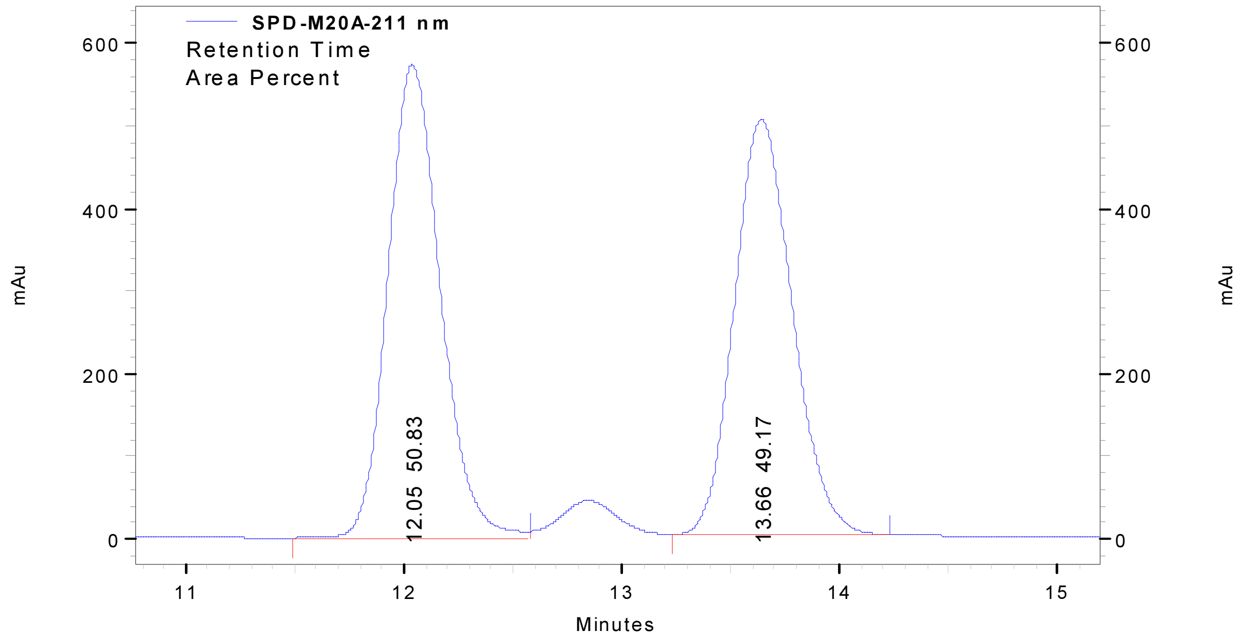

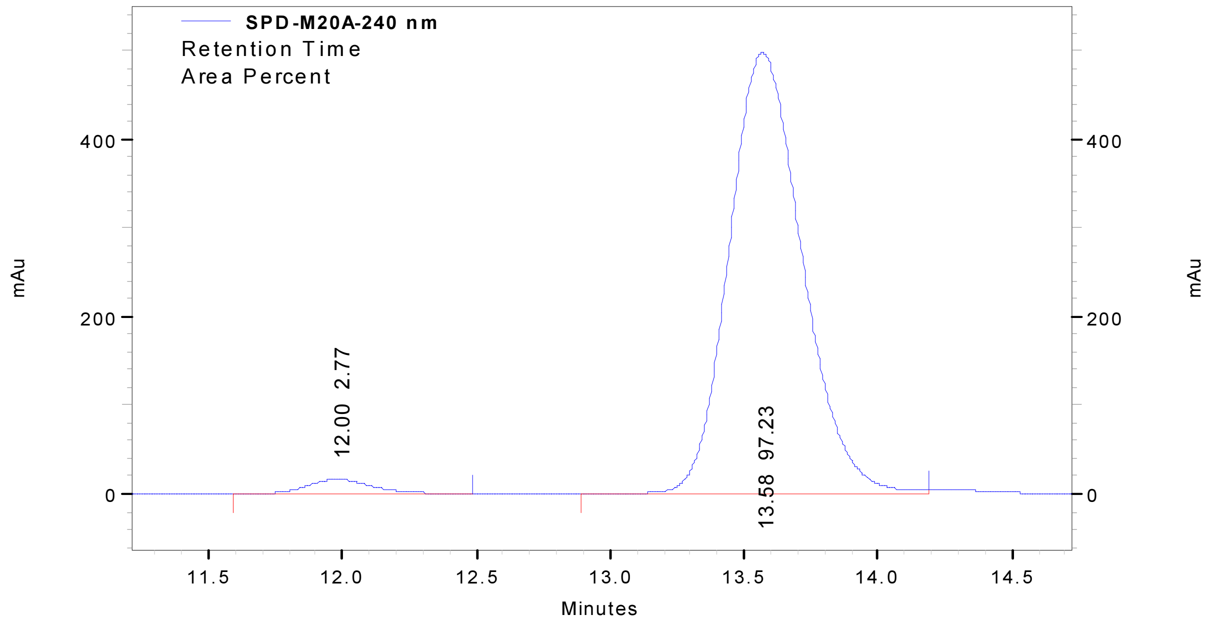

(*S*,*E*)-*N*-(3-(2-oxo-1-(4-(trifluoromethyl)phenyl)cyclohexyl)prop-1-en-1-yl)-*N*-phenylpivalamide (**3e**)

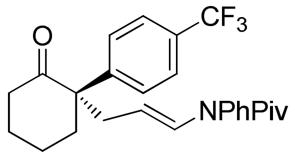

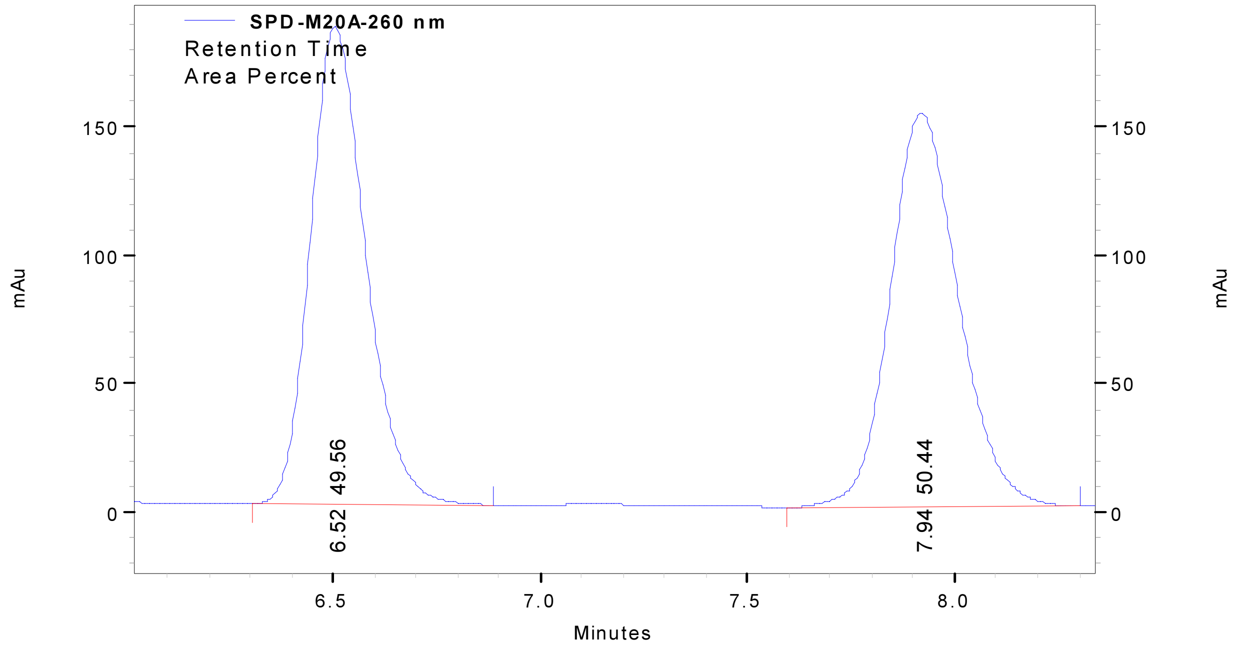

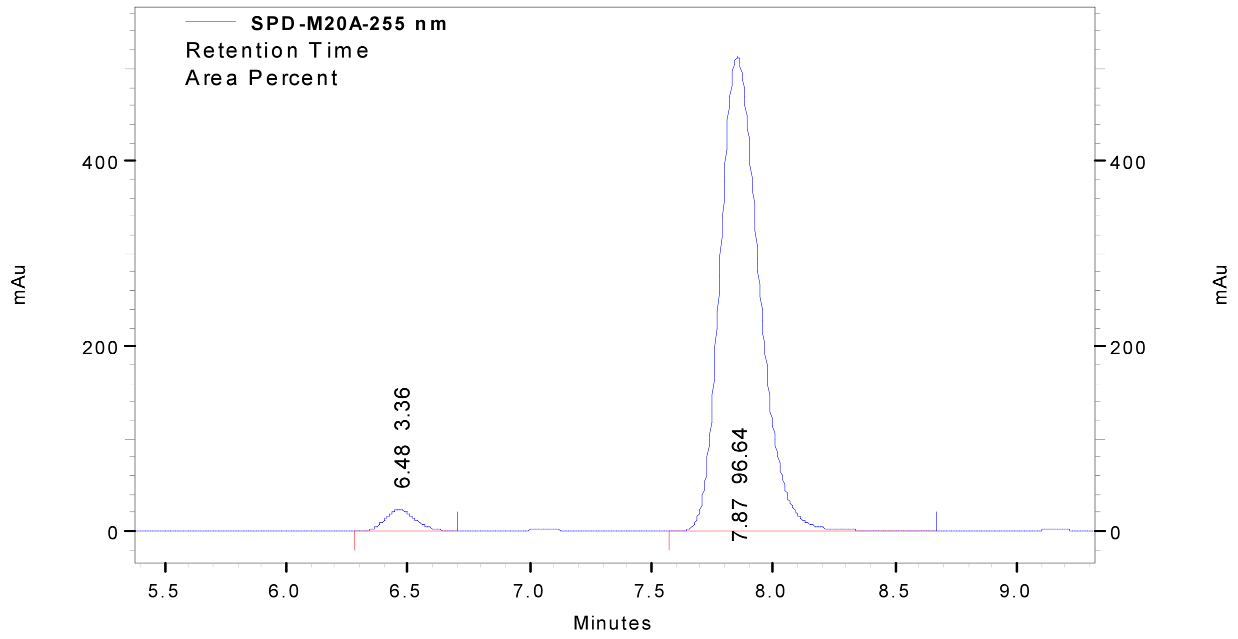

(*S*,*E*)-tert-butyl 4-(2-oxo-1-(3-(*N*-phenylpivalamido)allyl)cyclohexyl)benzoate (**3f**)

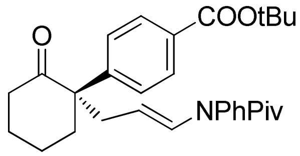

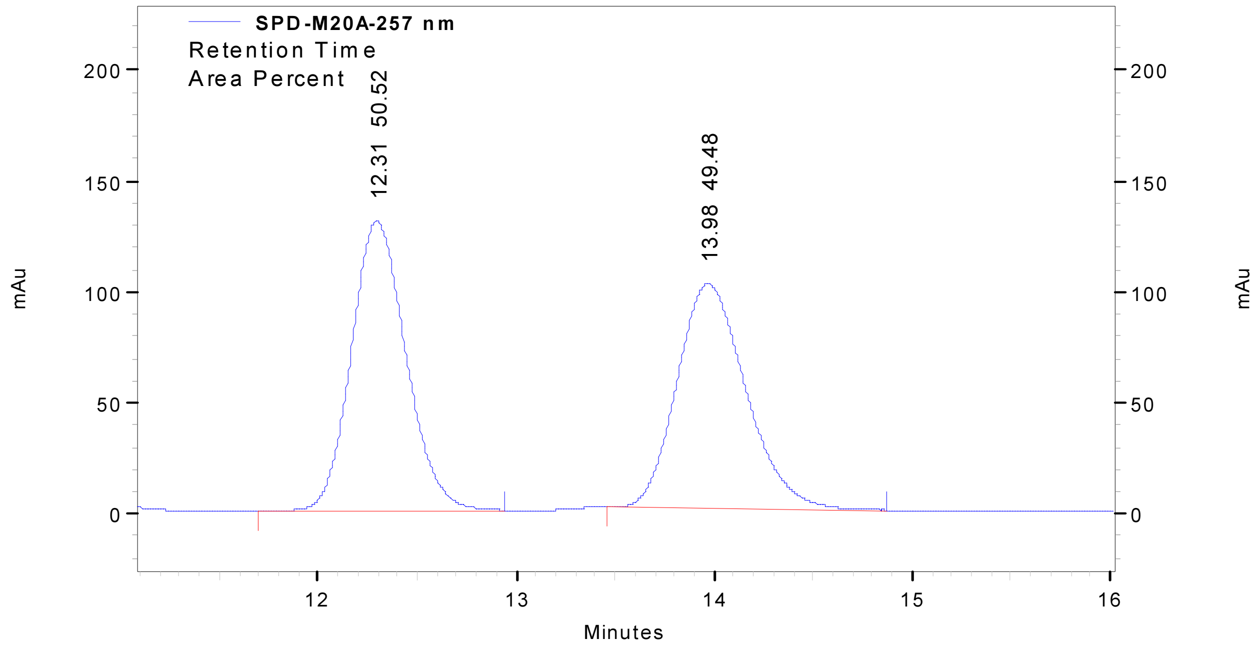

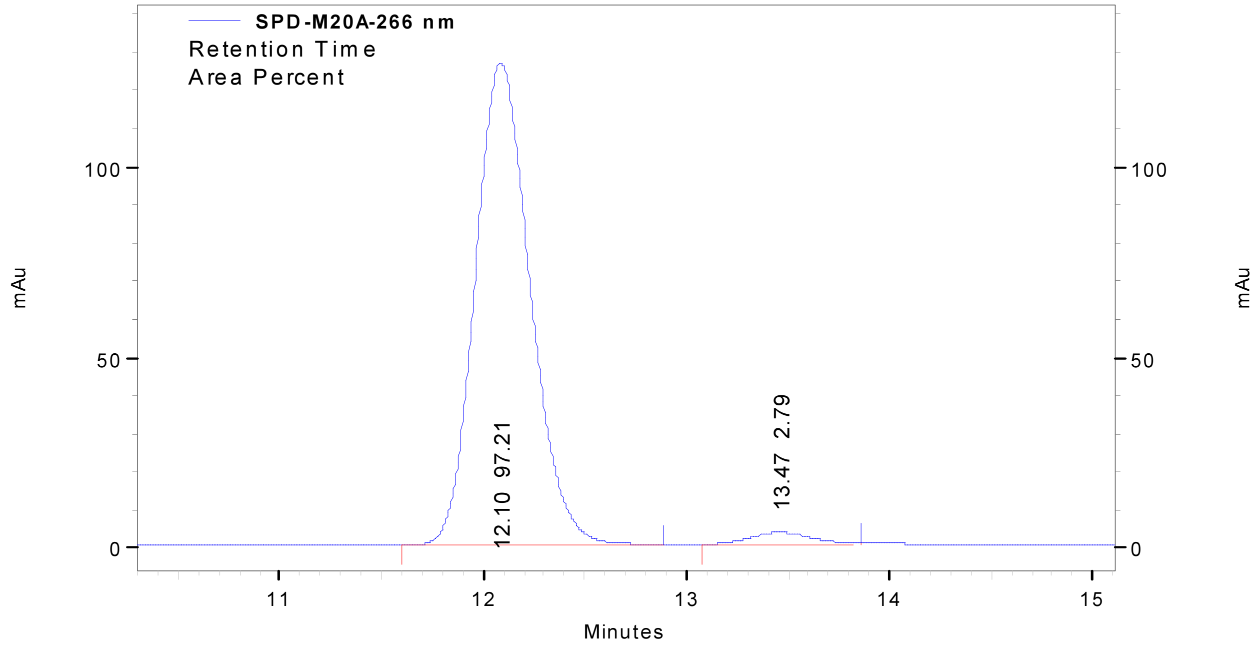

(*S,E*)-*N*-(3-(1-(benzo[d][1,3]dioxol-5-yl)-2-oxocyclohexyl)prop-1-en-1-yl)-*N*-phenylpivalamide (**3g**)

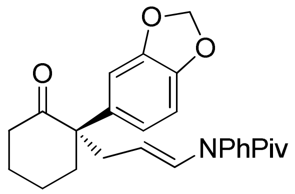

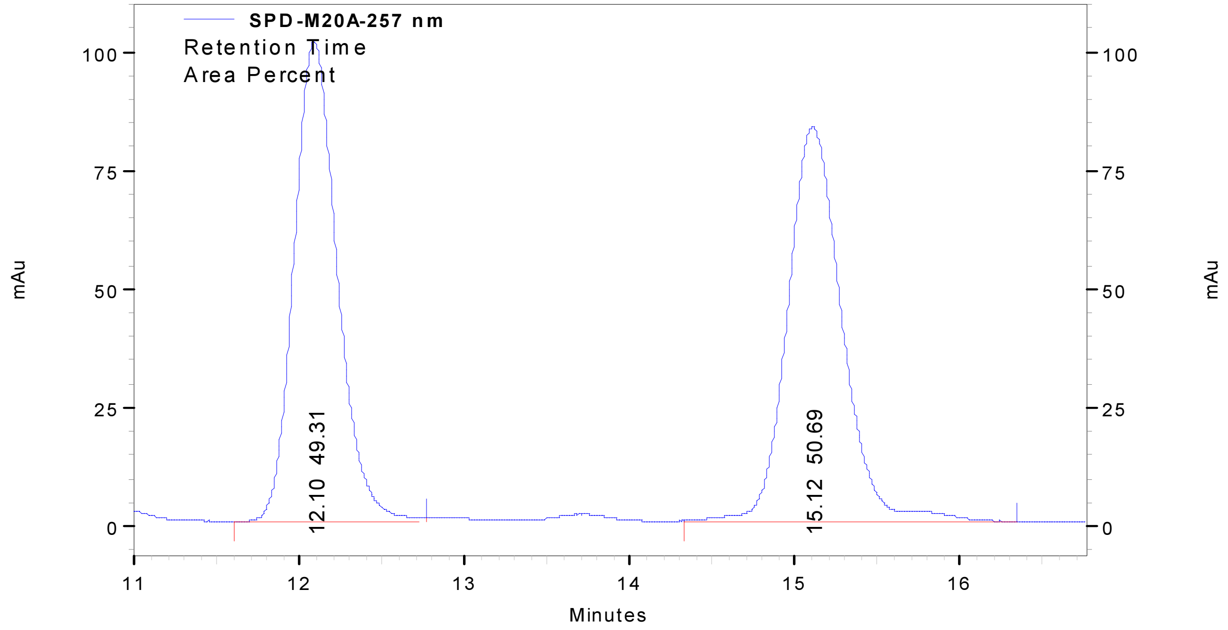

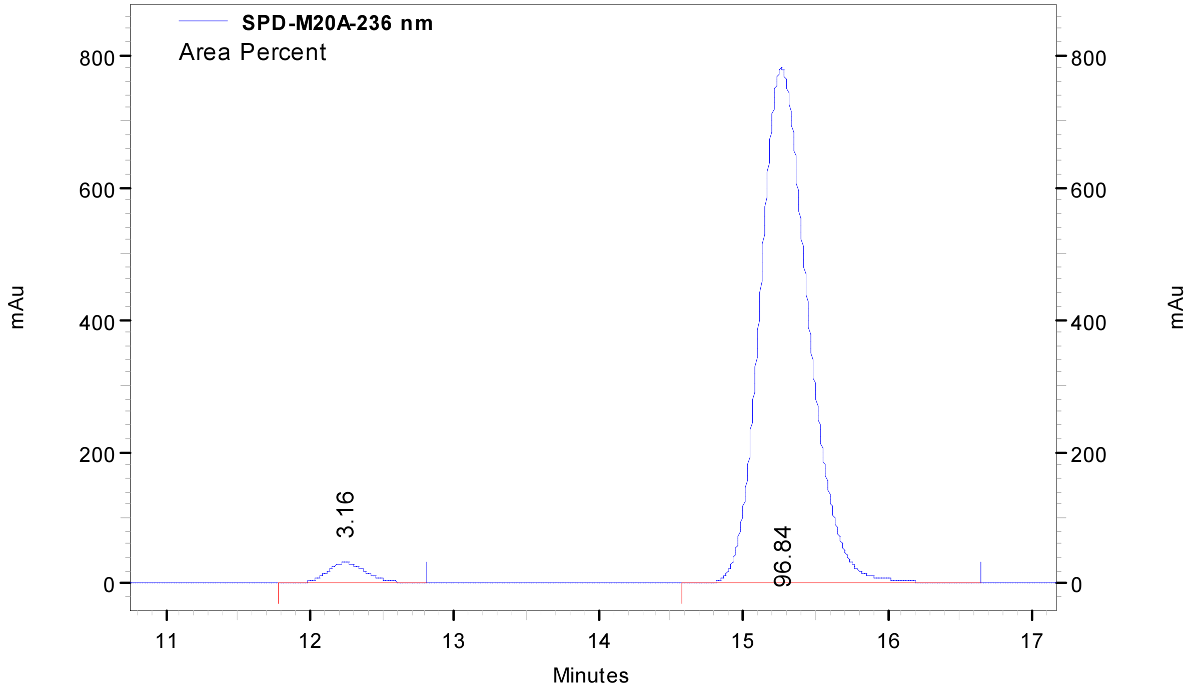

(*S*,*E*)-*N*-(3-(1-(3-methoxyphenyl)-2-oxocyclohexyl)prop-1-en-1-yl)-*N*-phenylpivalamide (**3h**)

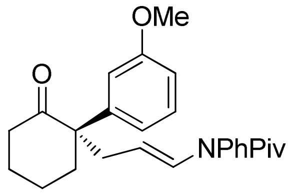

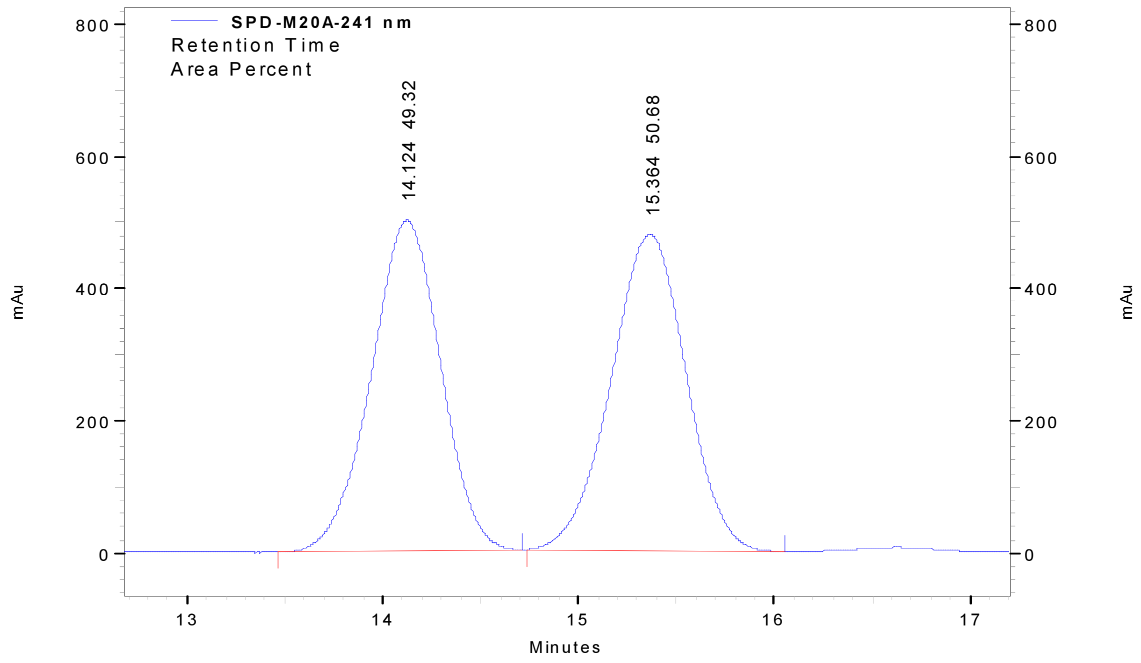

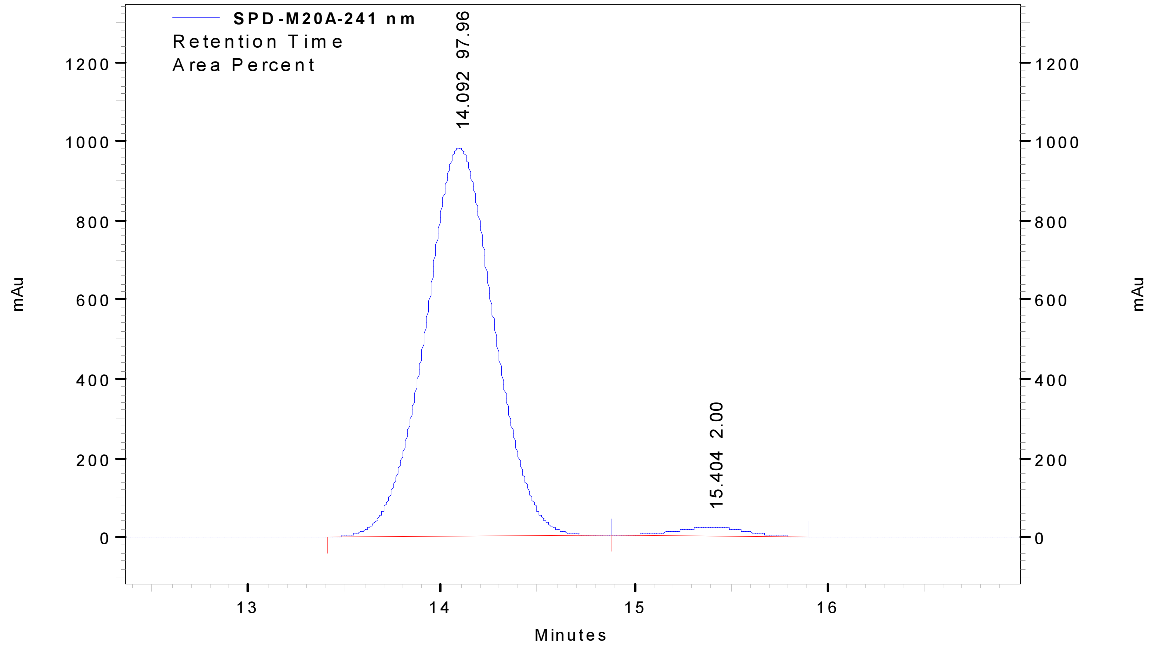

(*S,E*)-*N*-(3-(2-oxo-1-(o-tolyl)cyclohexyl)prop-1-en-1-yl)-*N*-phenylpivalamide (**3i**)

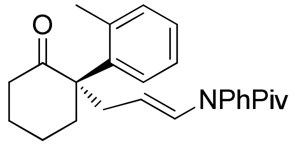

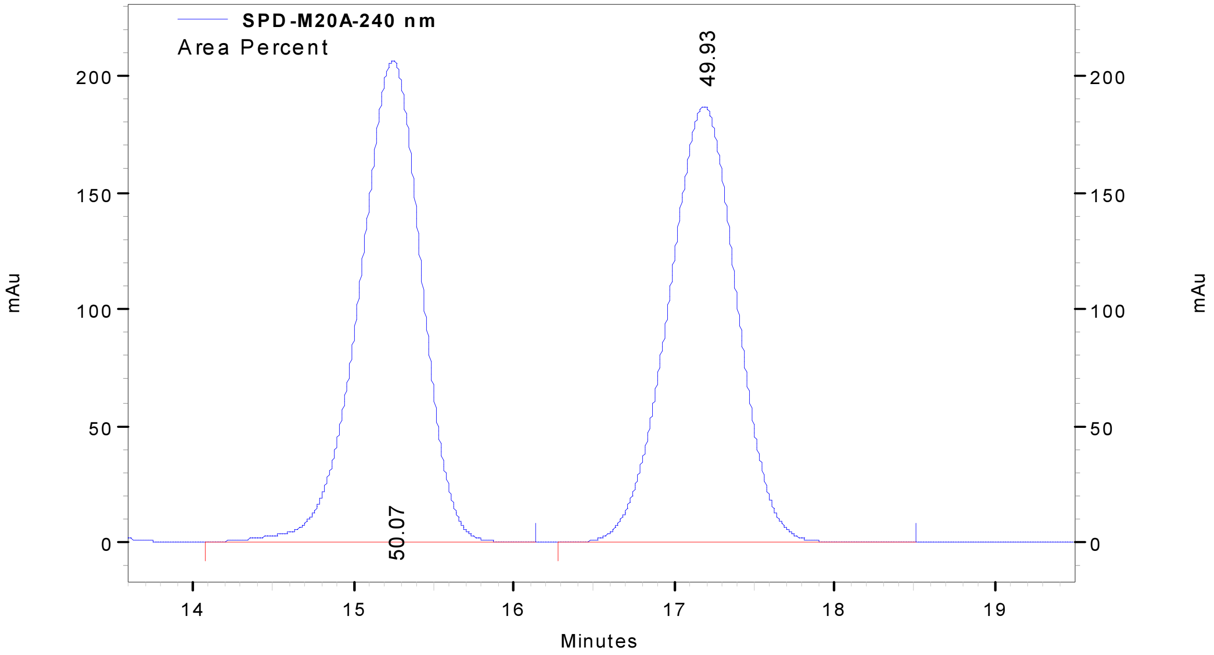

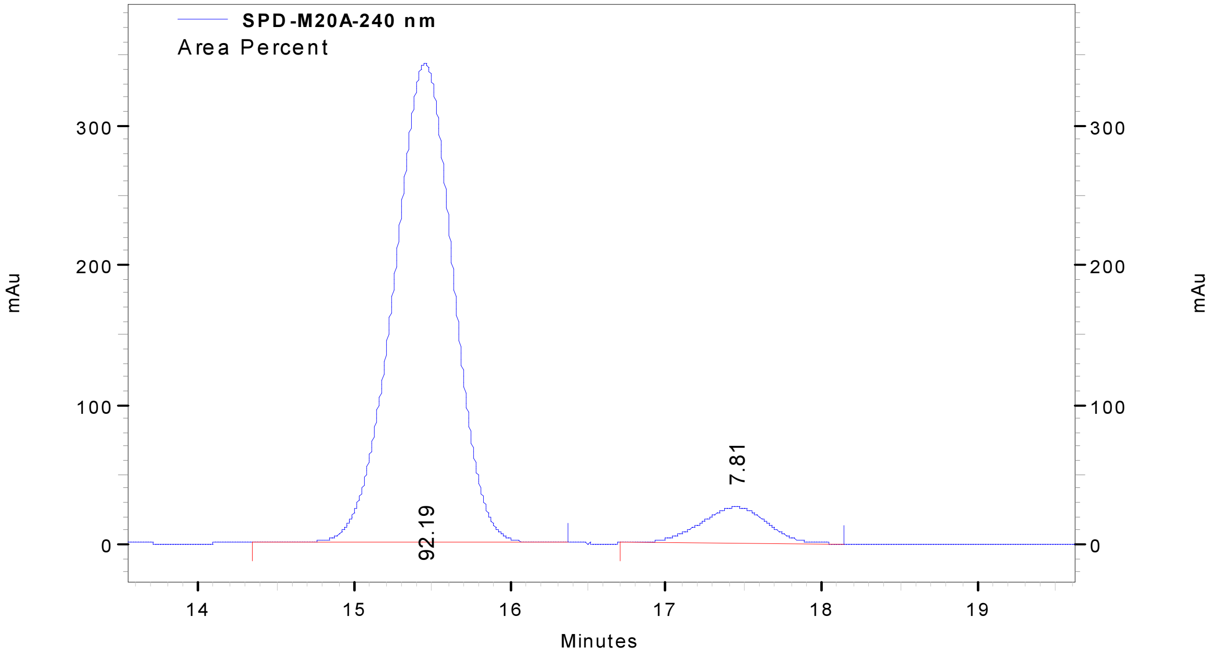

(*R,E*)-*N*-(3-(2-oxo-1-(thiophen-2-yl)cyclohexyl)prop-1-en-1-yl)-*N*-phenylpivalamide (**3j**)

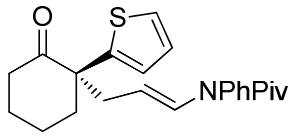

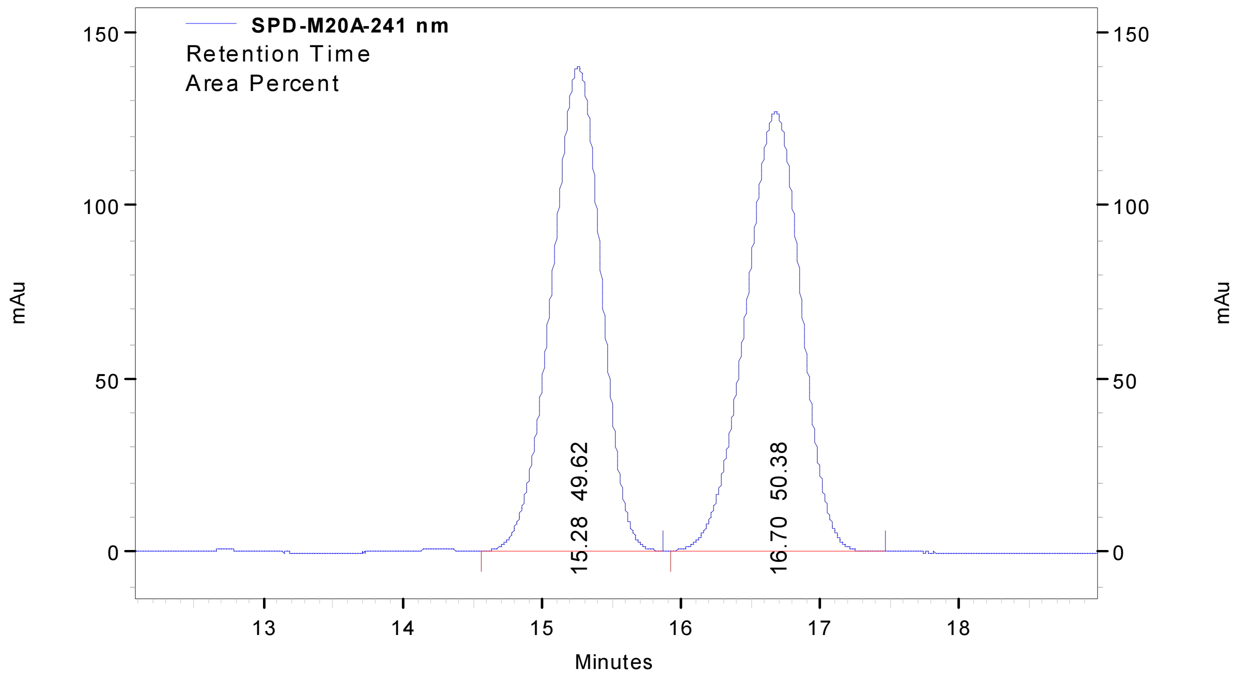

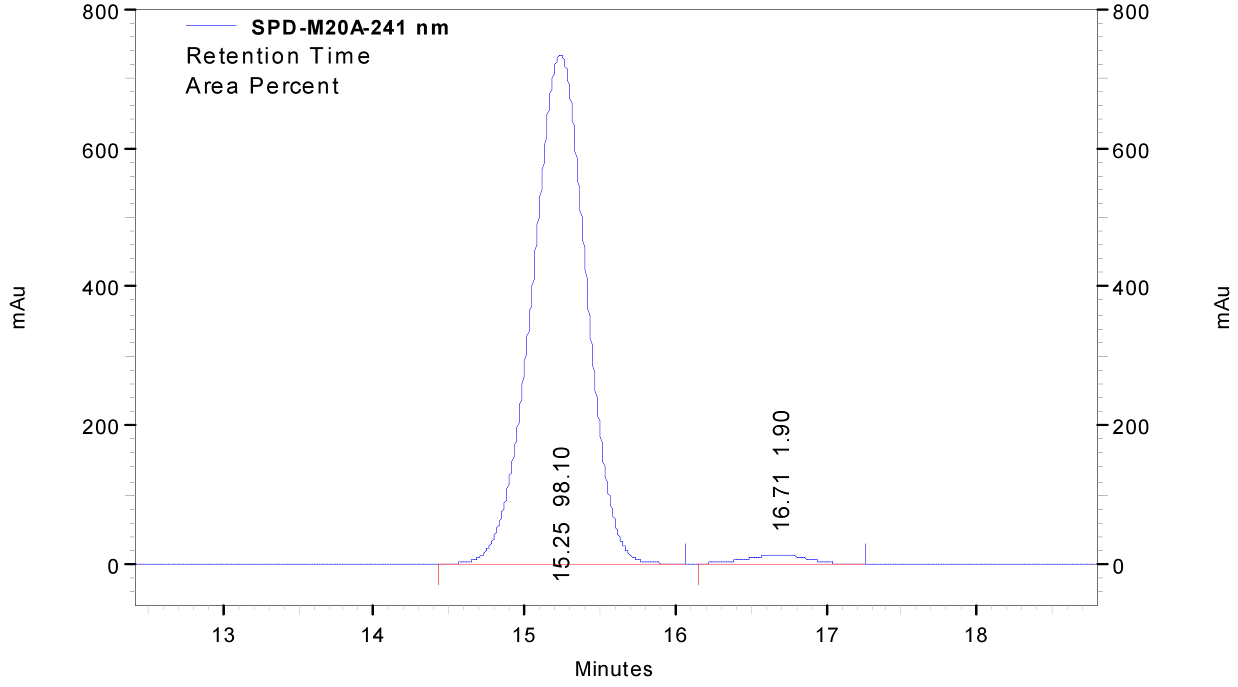

(*S*,*E*)-*N*-(3-(2-oxo-1-phenylcyclopentyl)prop-1-en-1-yl)-*N*-phenylpivalamide (**3k**)

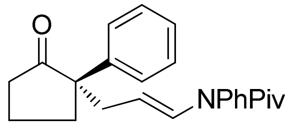

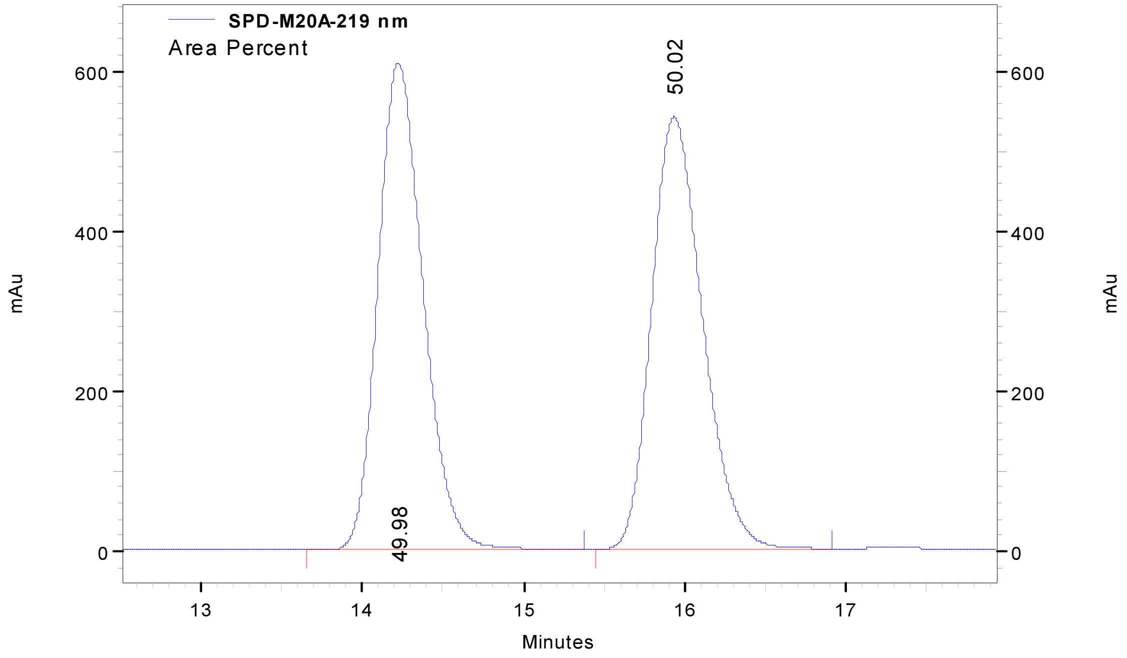

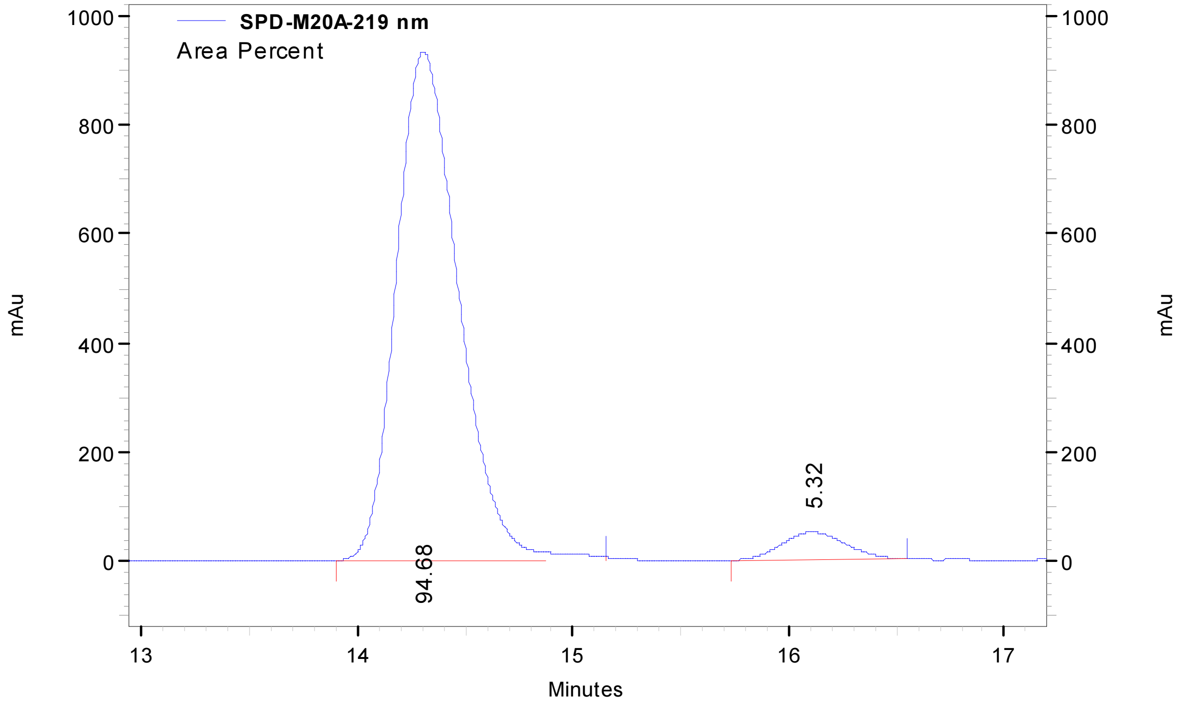

(*S,E*)-*N*-(3-(4-oxo-3-phenyltetrahydro-2H-pyran-3-yl)prop-1-en-1-yl)-*N*-phenylpivalamide (**3l**)

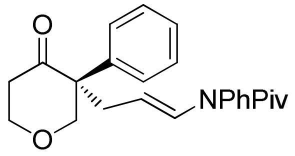

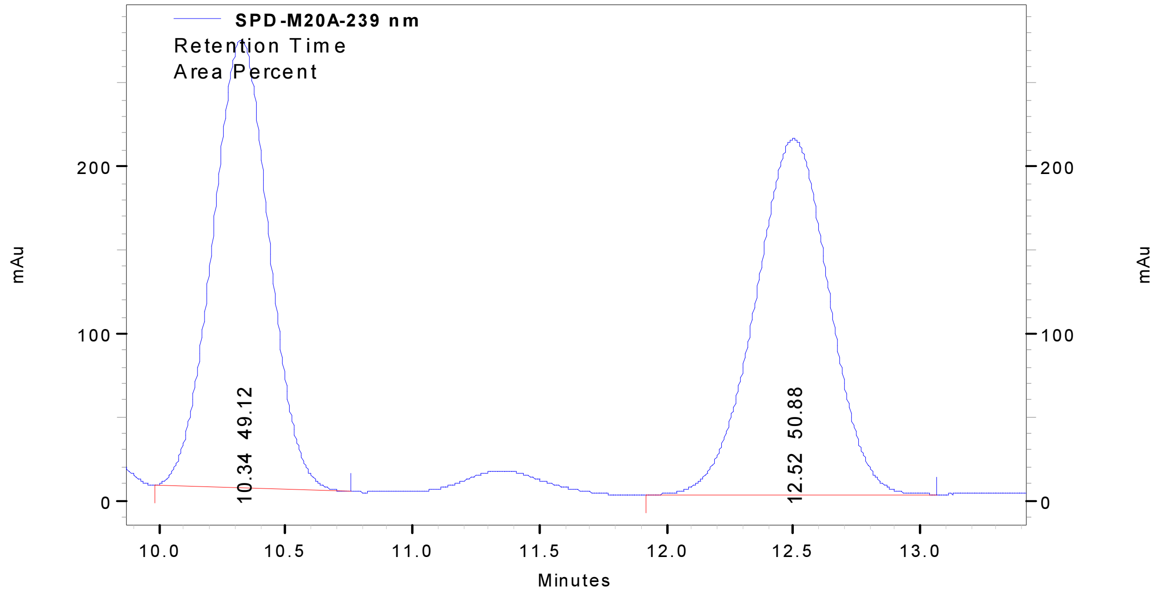

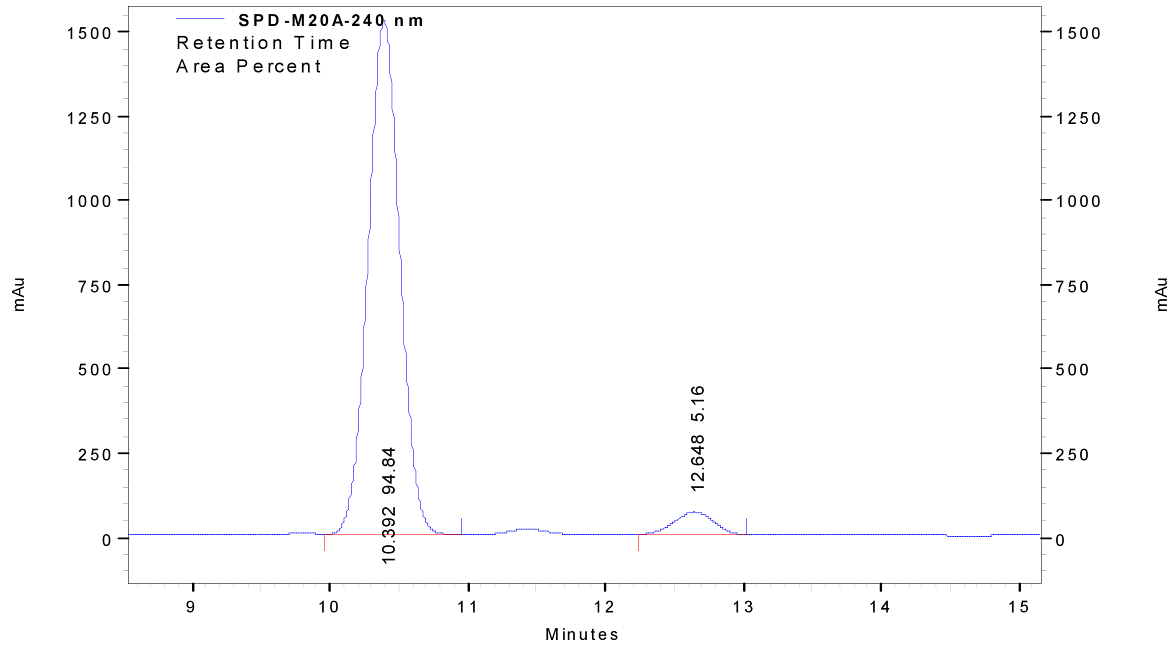

N-((*E*)-3-((*S*)-2-oxo-1-((*E*)-styryl)cyclohexyl)prop-1-en-1-yl)-*N*-phenylpivalamide (**3m**)

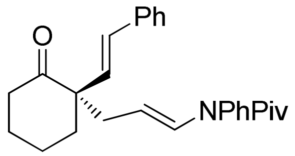

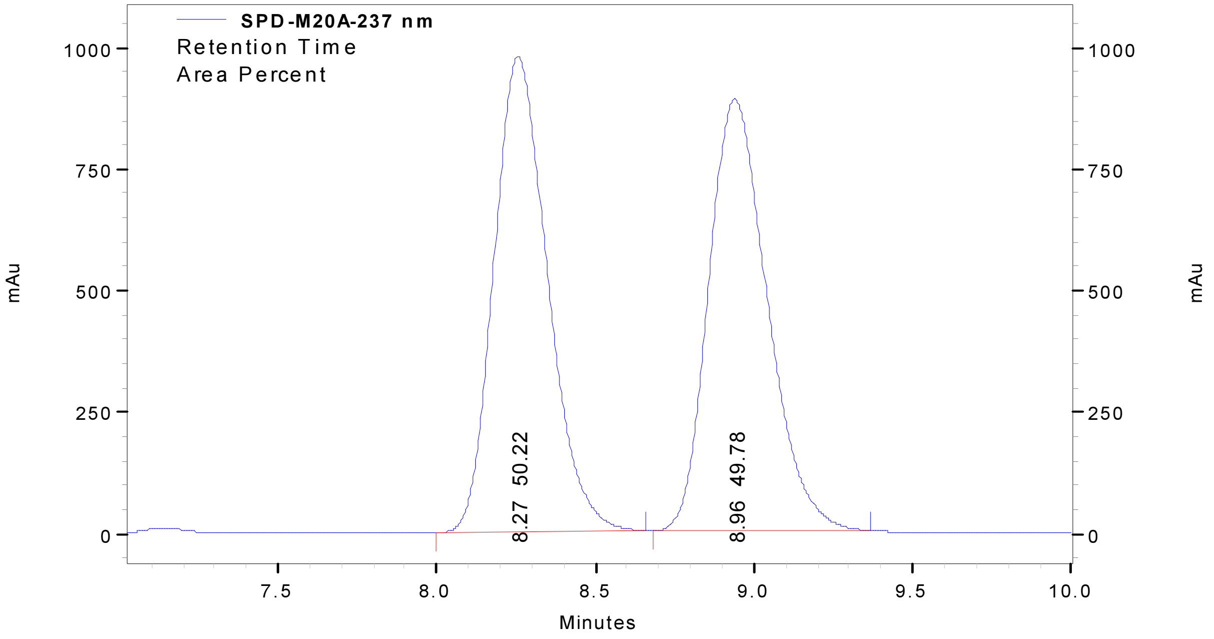

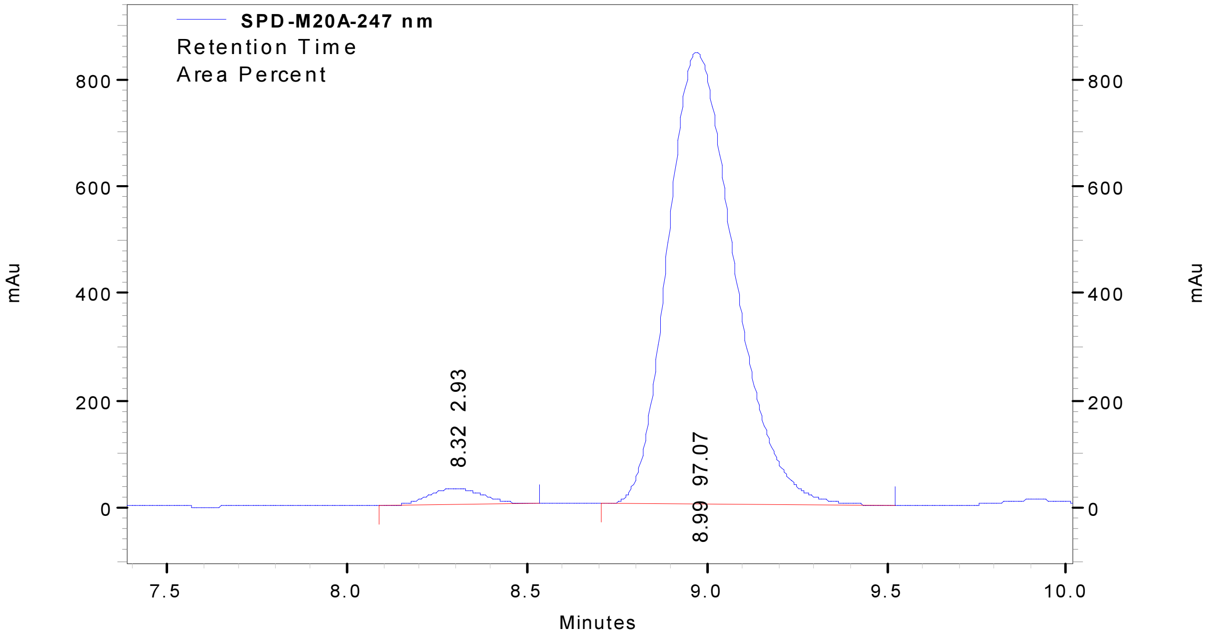

N-((*E*)-3-((*S*)-2-oxo-1-((*Z*)-styryl)cyclohexyl)prop-1-en-1-yl)-*N*-phenylpivalamide (**3n**)

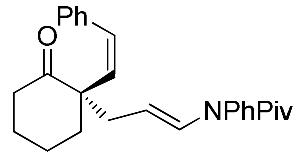

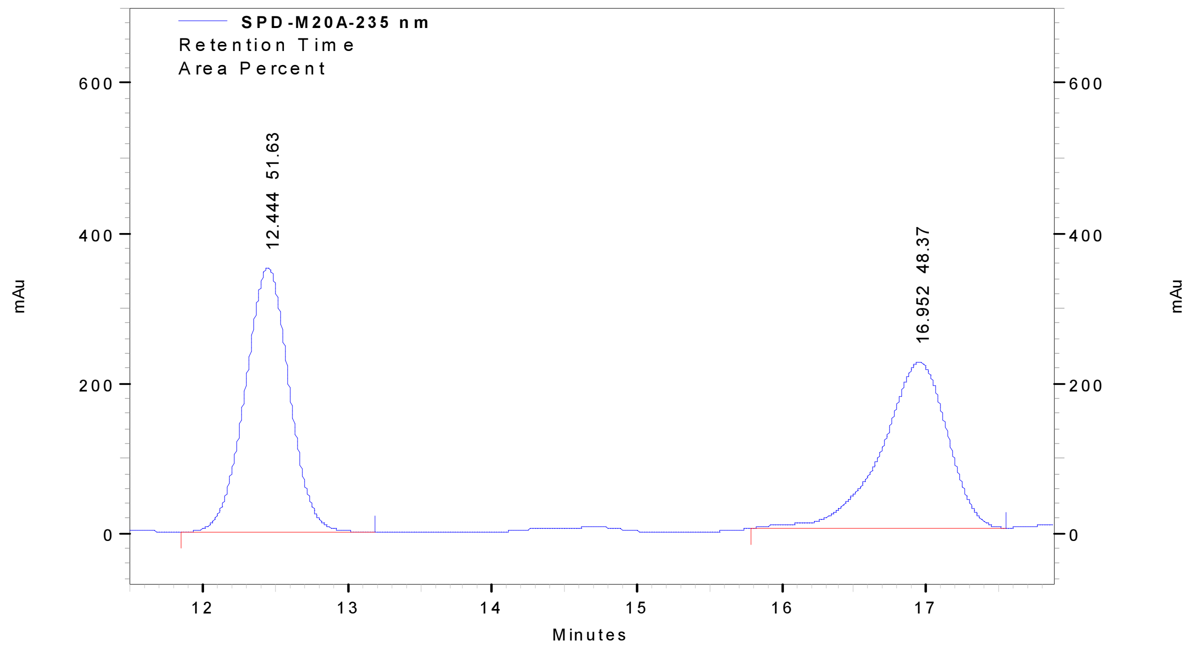

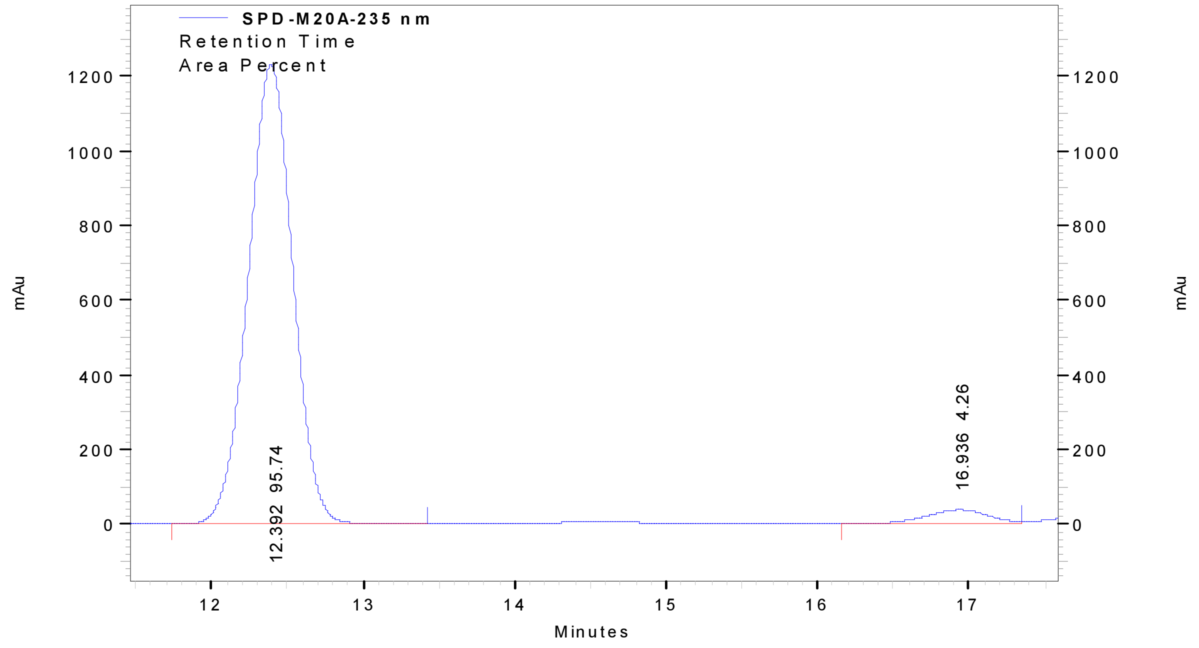

(*S*,*E*)-*N*-(3-(2-oxo-1-vinylcyclohexyl)prop-1-en-1-yl)-*N*-phenylpivalamide (**3o**)

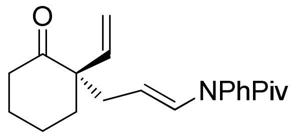

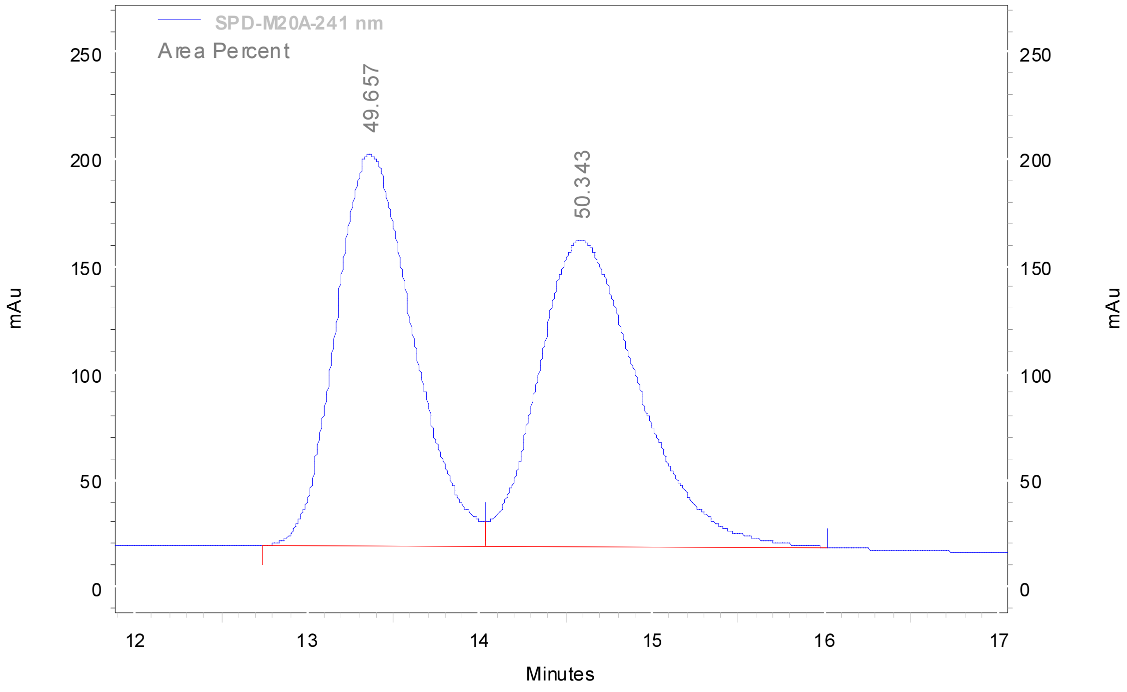

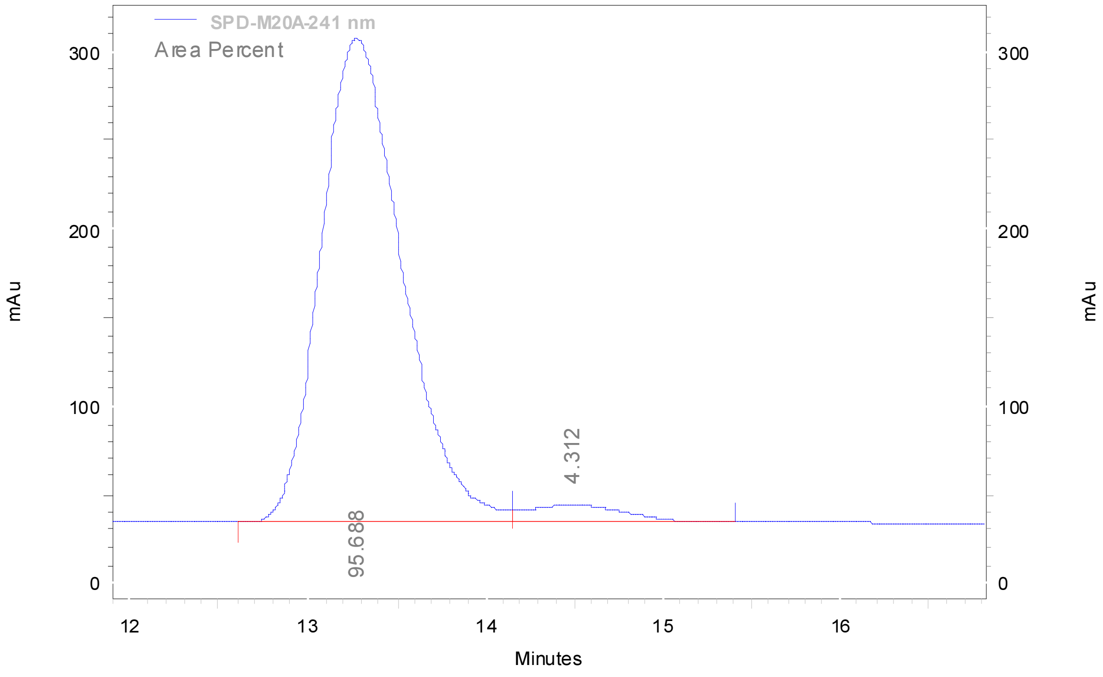

(*S*,*E*)-*N*-(3-(1-(2-methylprop-1-en-1-yl)-2-oxocyclohexyl)prop-1-en-1-yl)-*N*-phenylpivalamide (**3p**)

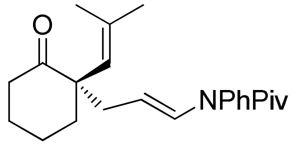

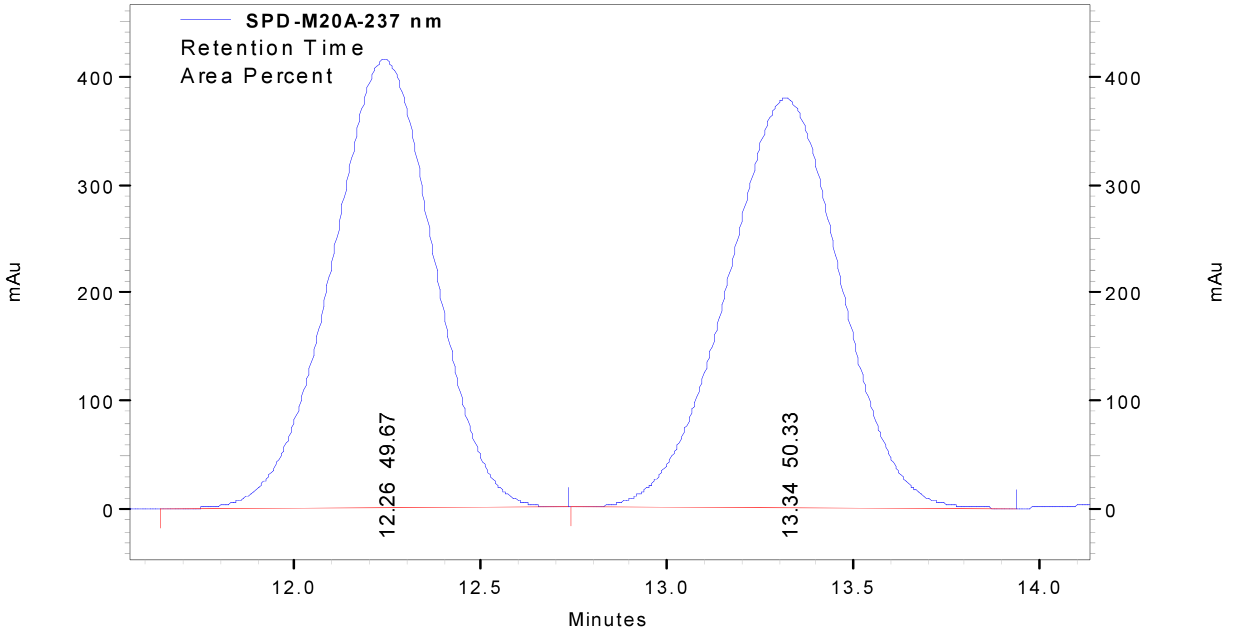

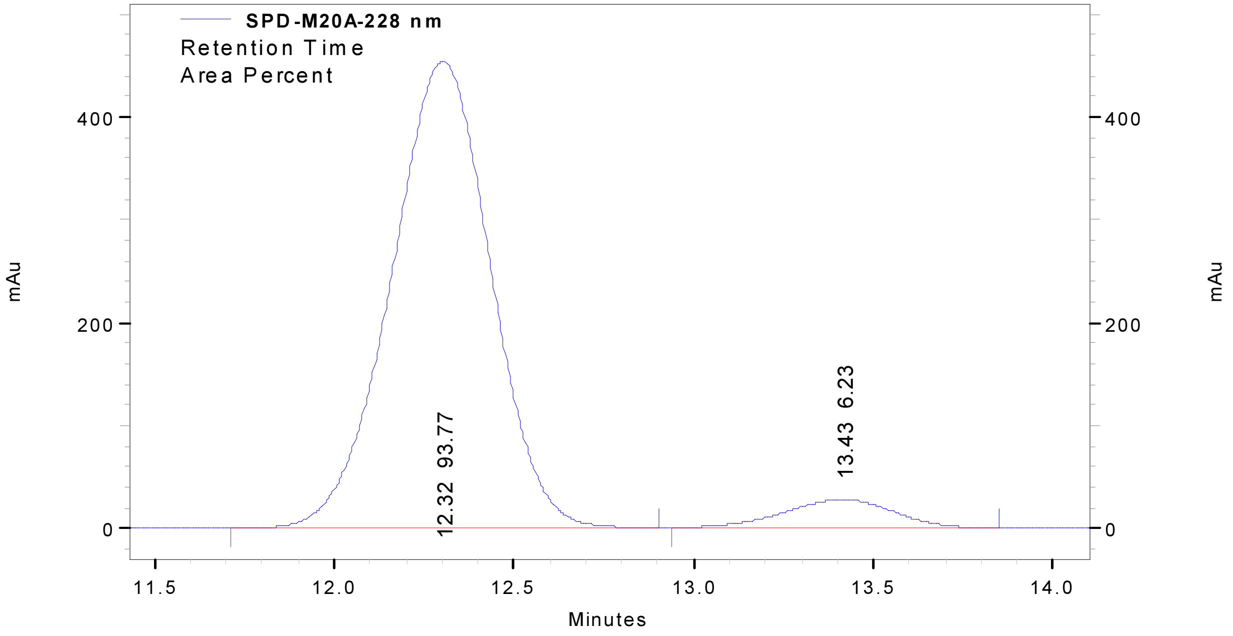

(*S*,*E*)-*N*-(3-(2-oxo-[1,1'-bi(cyclohexan)]-1'-en-1-yl)prop-1-en-1-yl)-*N*-phenylpivalamide (**3q**)

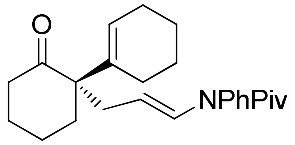

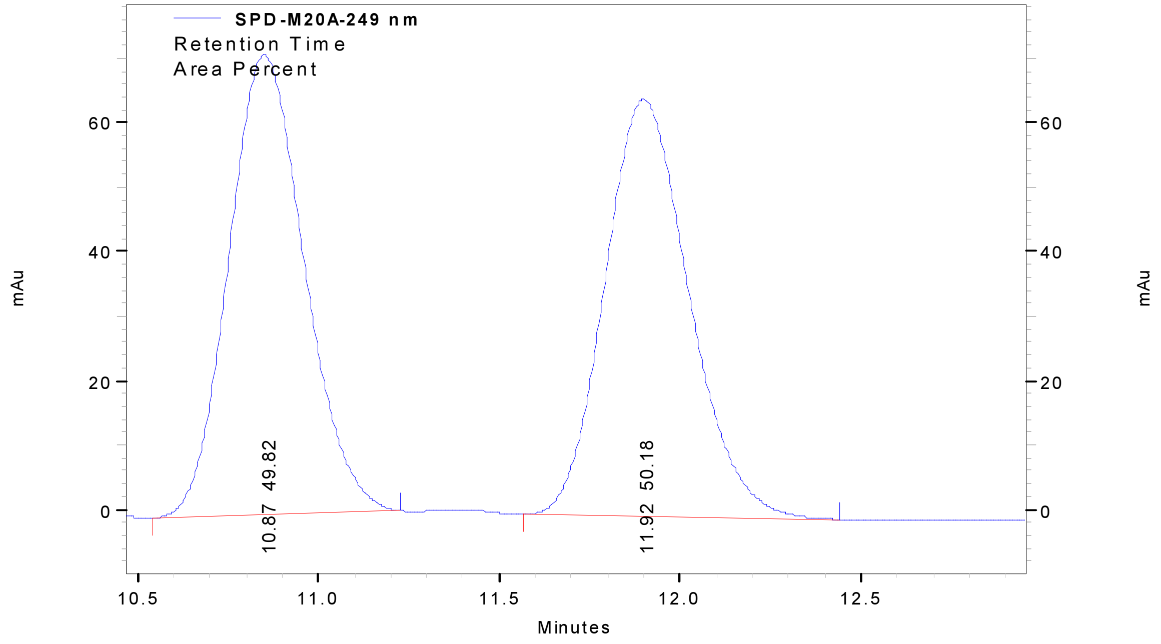

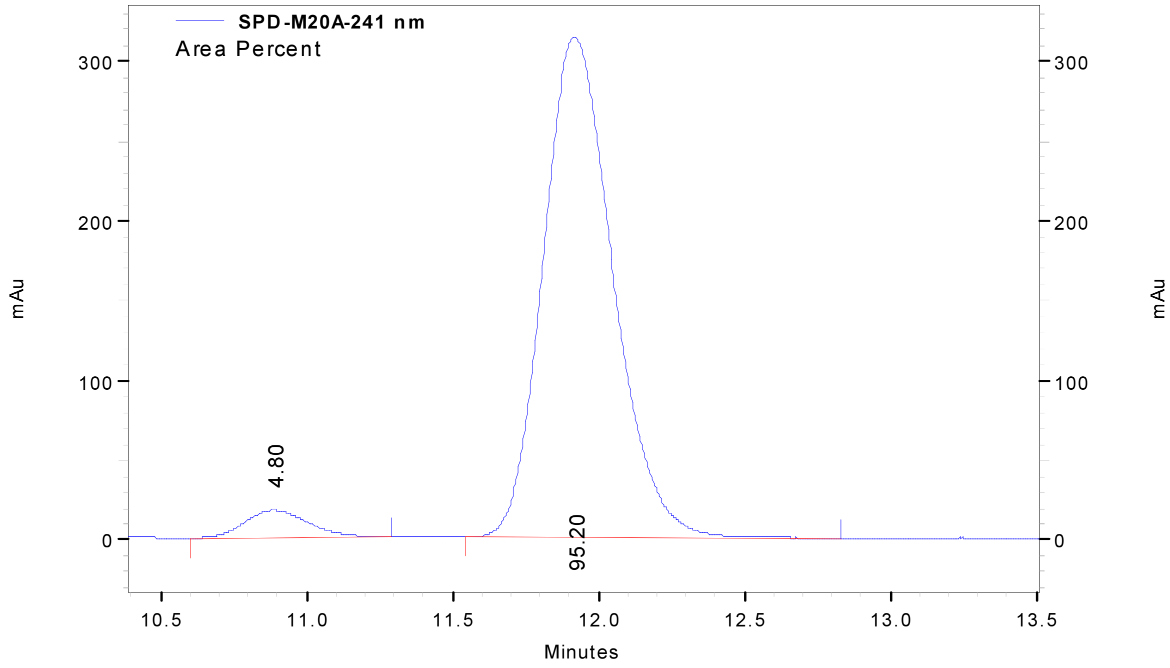

(*S*,*E*)-*N*-(3-(2-oxo-1-(phenylethynyl)cyclohexyl)prop-1-en-1-yl)-*N*-phenylpivalamide (**3r**)

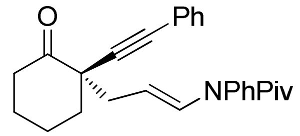

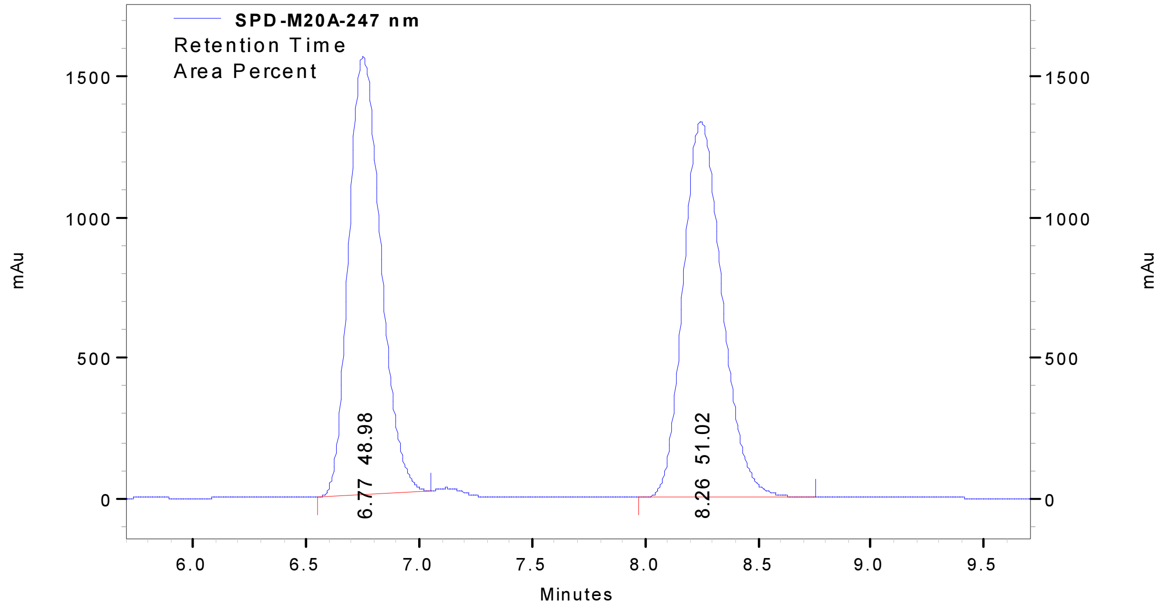

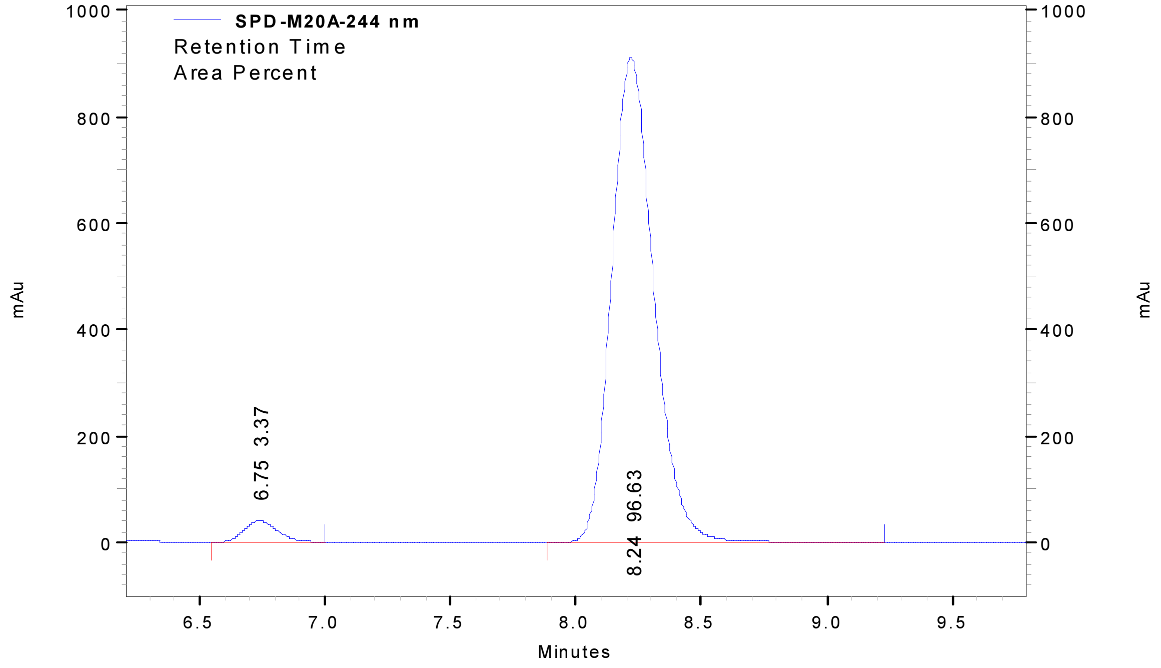

(*S*,*E*)-*N*-(3-(1-(hex-1-yn-1-yl)-2-oxocyclohexyl)prop-1-en-1-yl)-*N*-phenylpivalamide (**3s**)

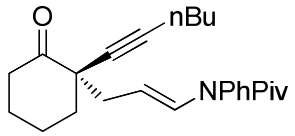

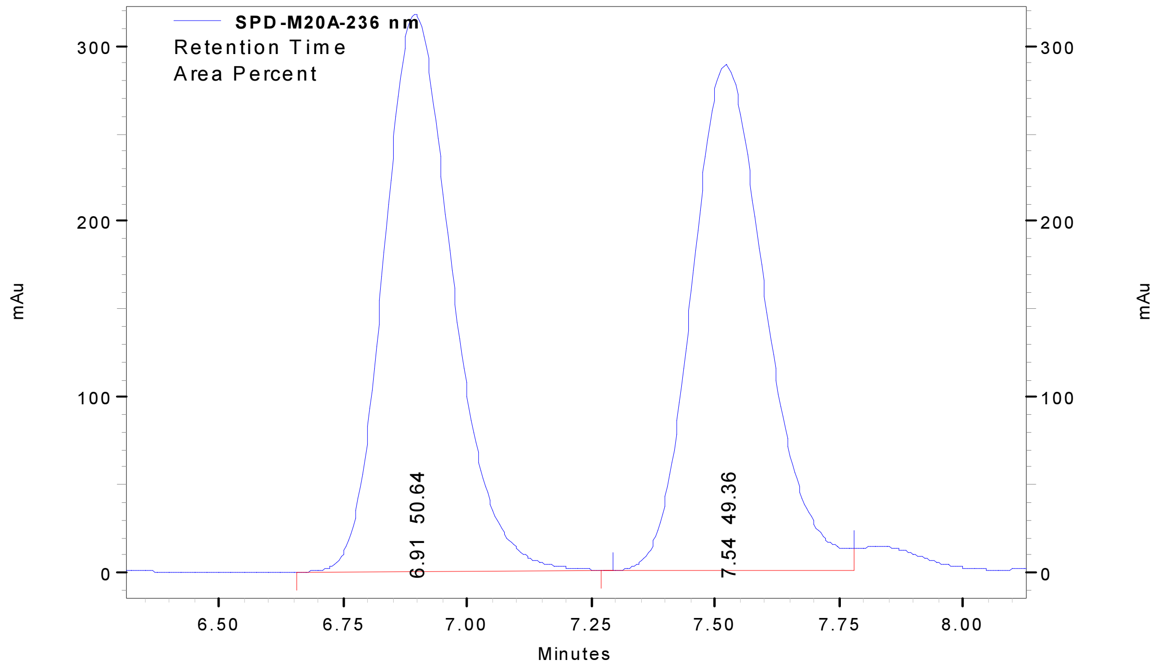

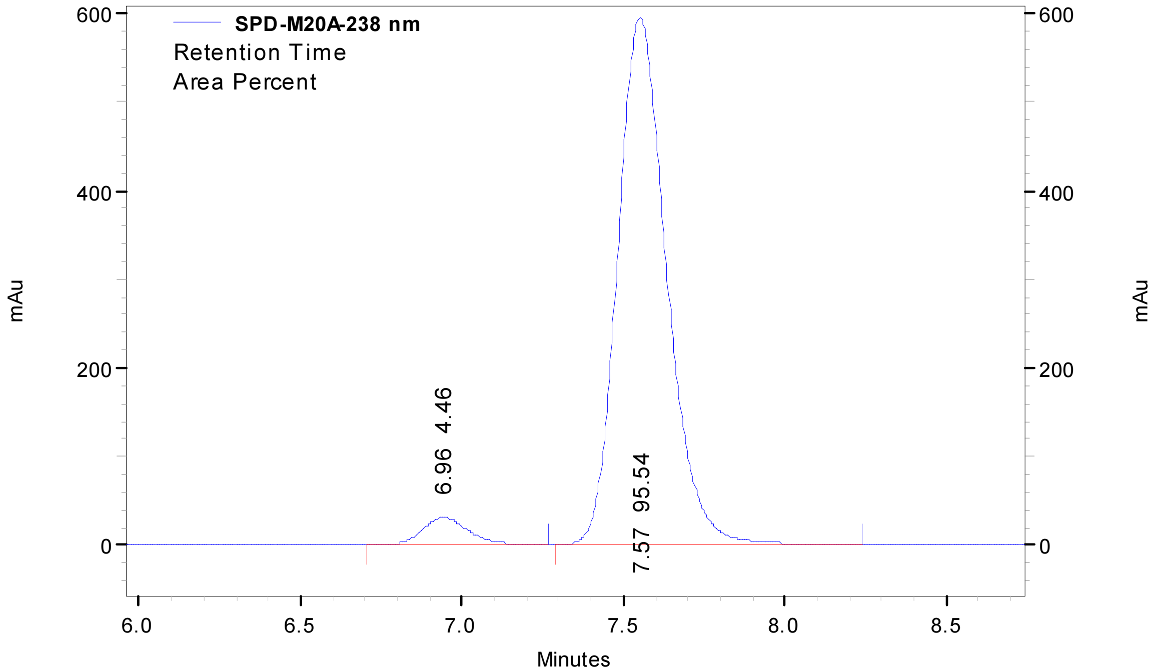

(*S*,*E*)-*N*-(3-(1-methyl-2-oxocyclohexyl)prop-1-en-1-yl)-*N*-phenylpivalamide (**3t**)

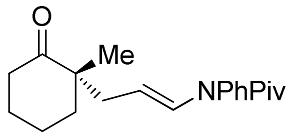

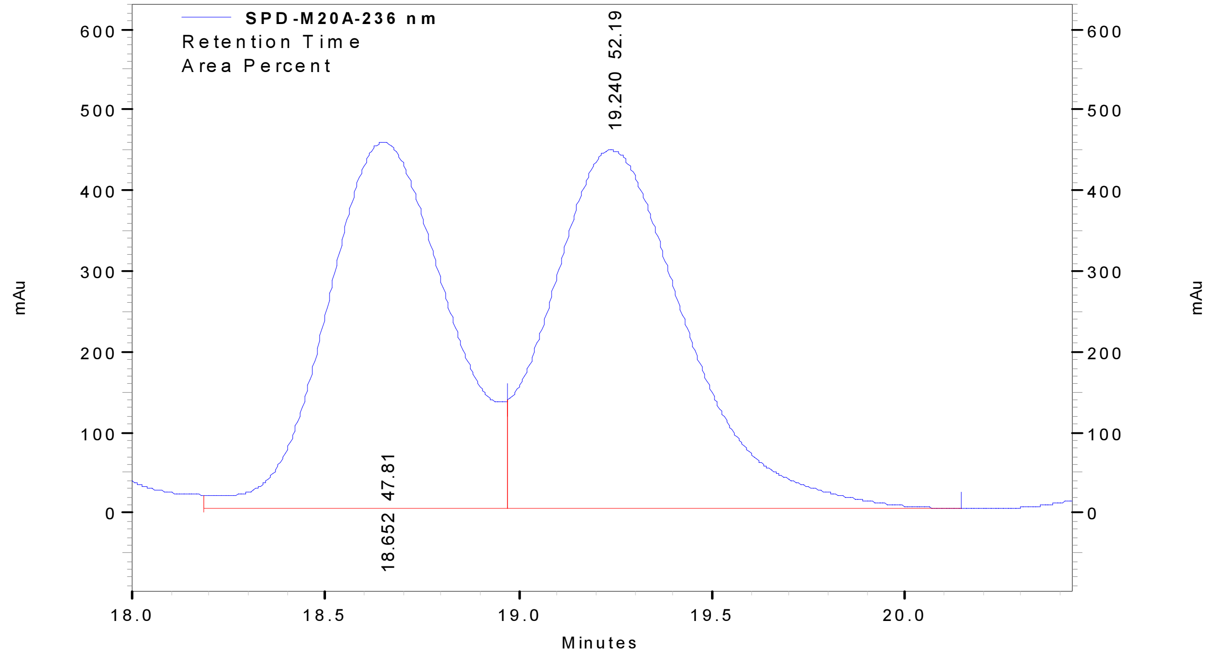

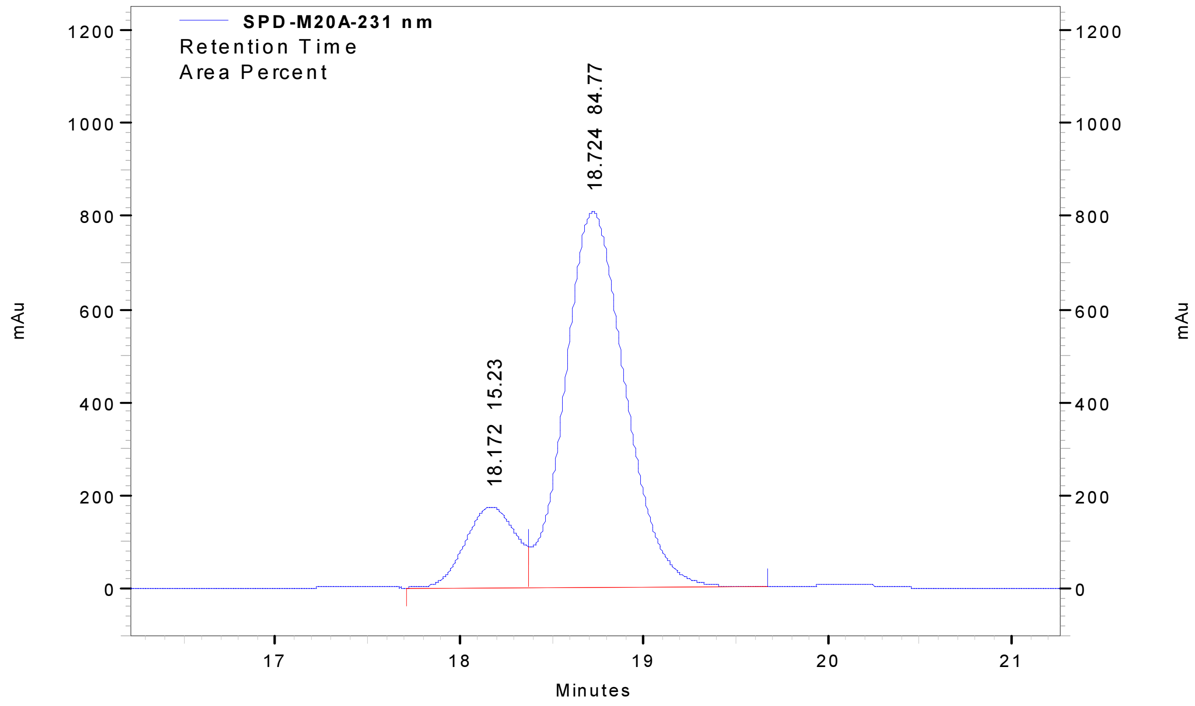

(*S,E*)-*N*-(3-(1-butyl-2-oxocyclohexyl)prop-1-en-1-yl)-*N*-phenylpivalamide (**3u**)

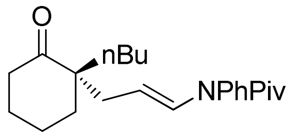

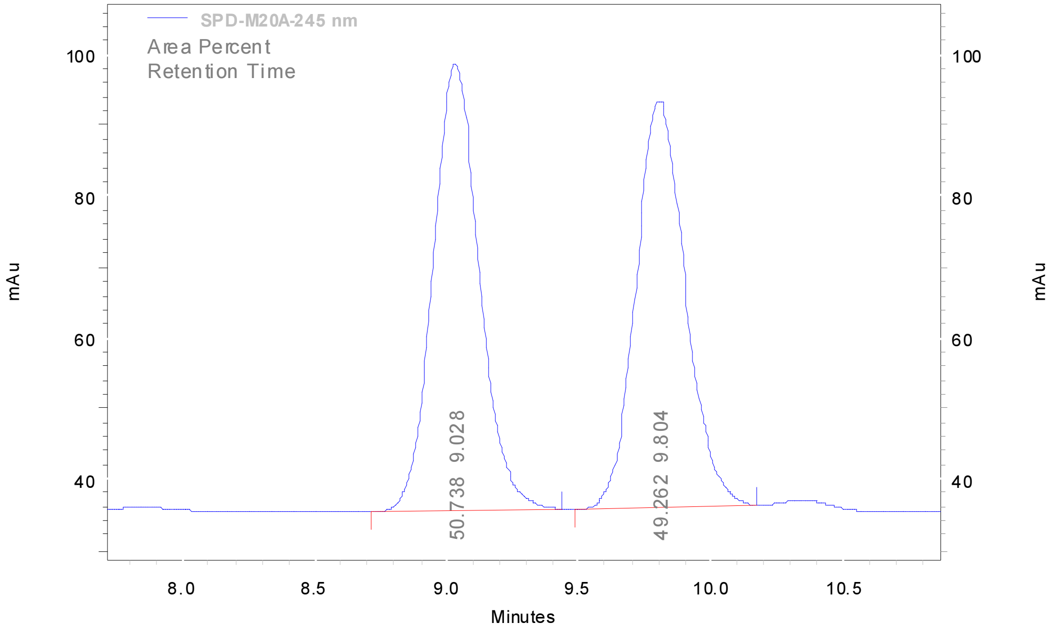

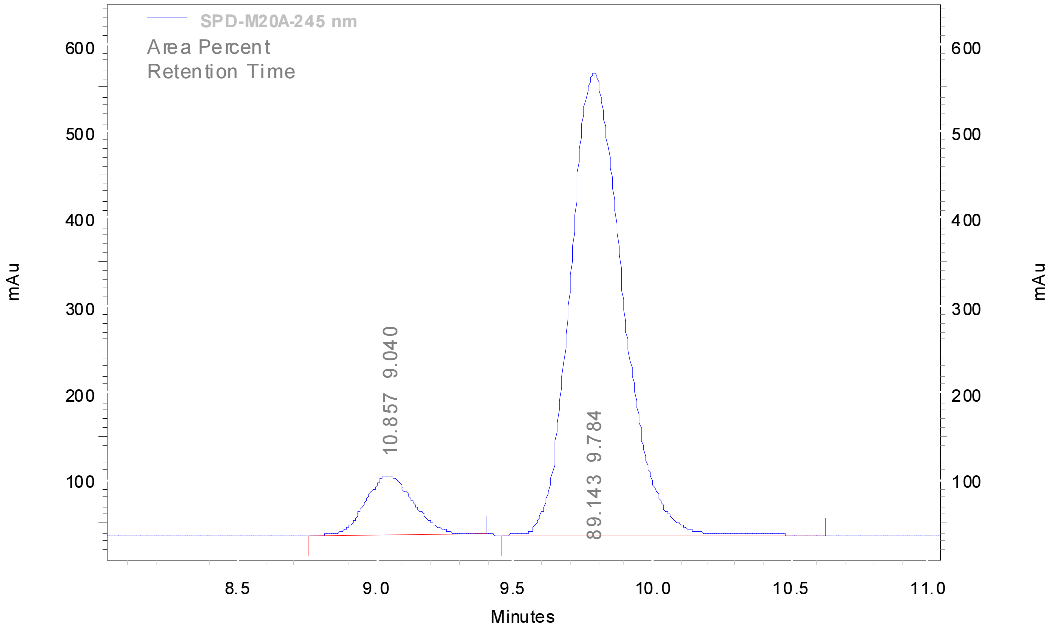

(*S*)-2-(1-(benzo[d][1,3]dioxol-5-yl)-2-oxocyclohexyl)acetaldehyde (**7g**)

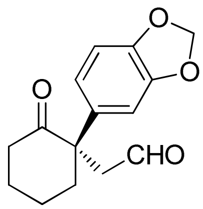

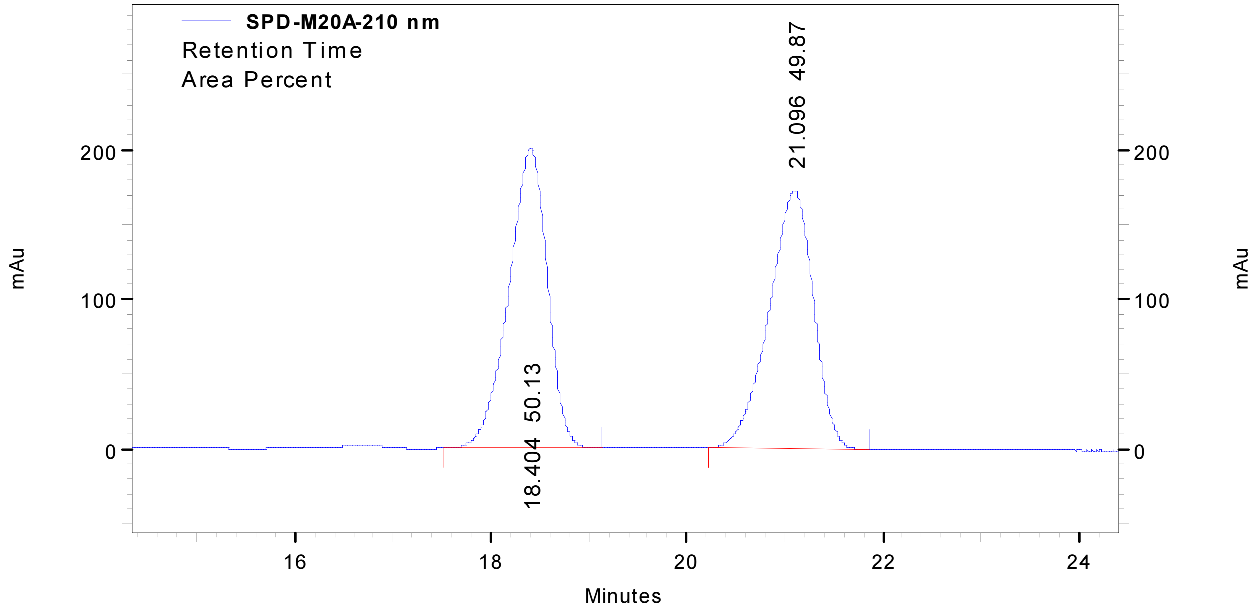

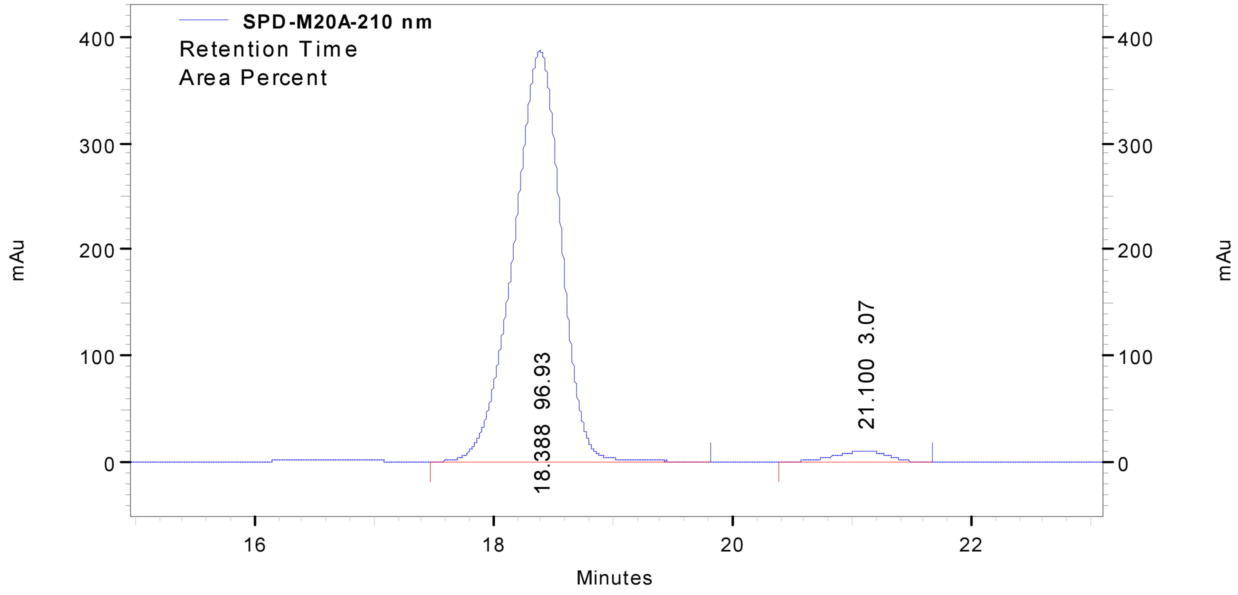

(*S*)-3-(2-oxo-1-phenylcyclohexyl)propanal (**4a**)

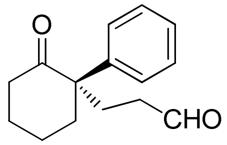

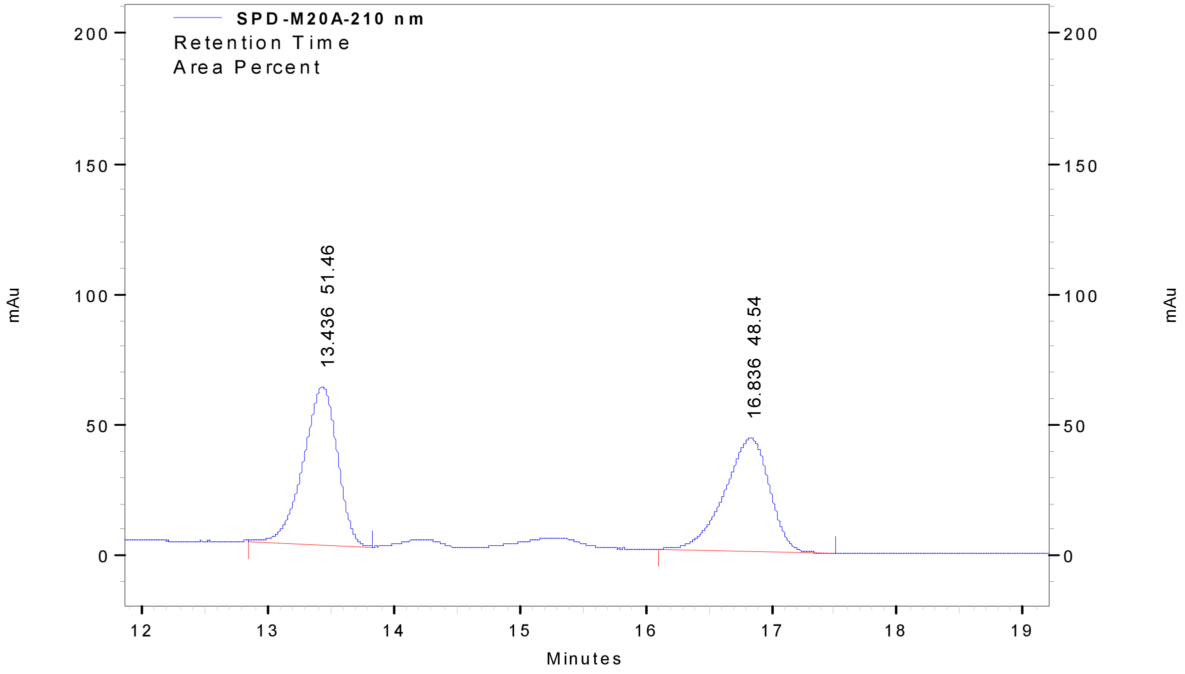

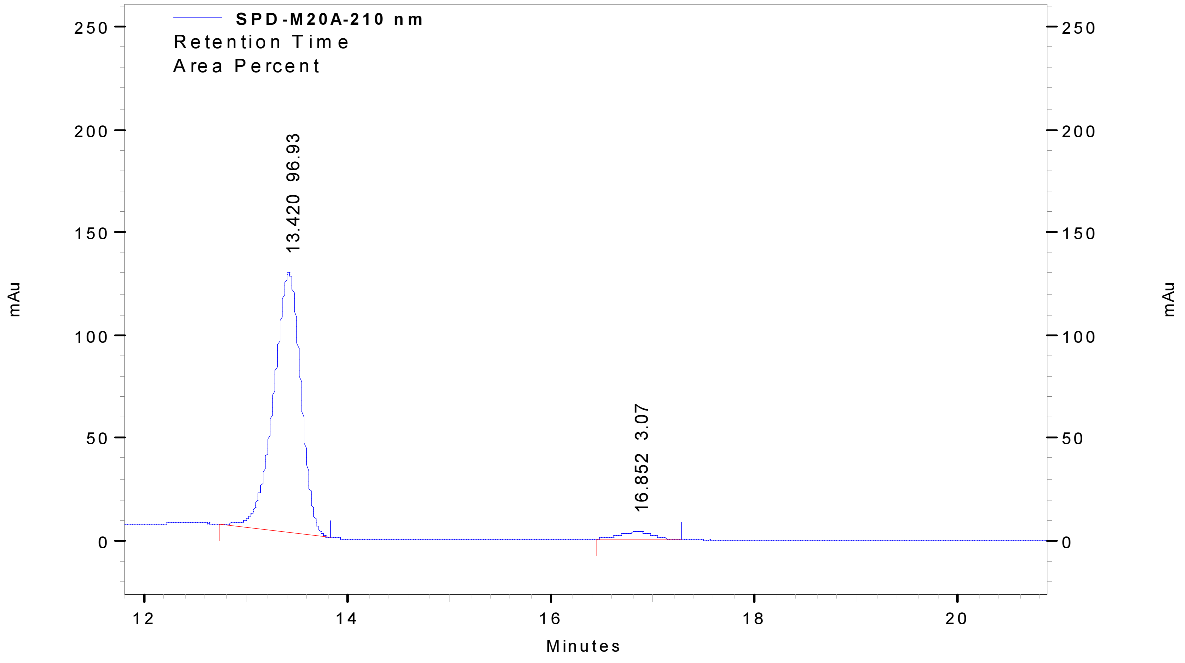

(*S*)-2-(2-oxo-1-phenylcyclohexyl)acetaldehyde (**7a**)

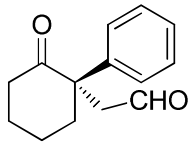

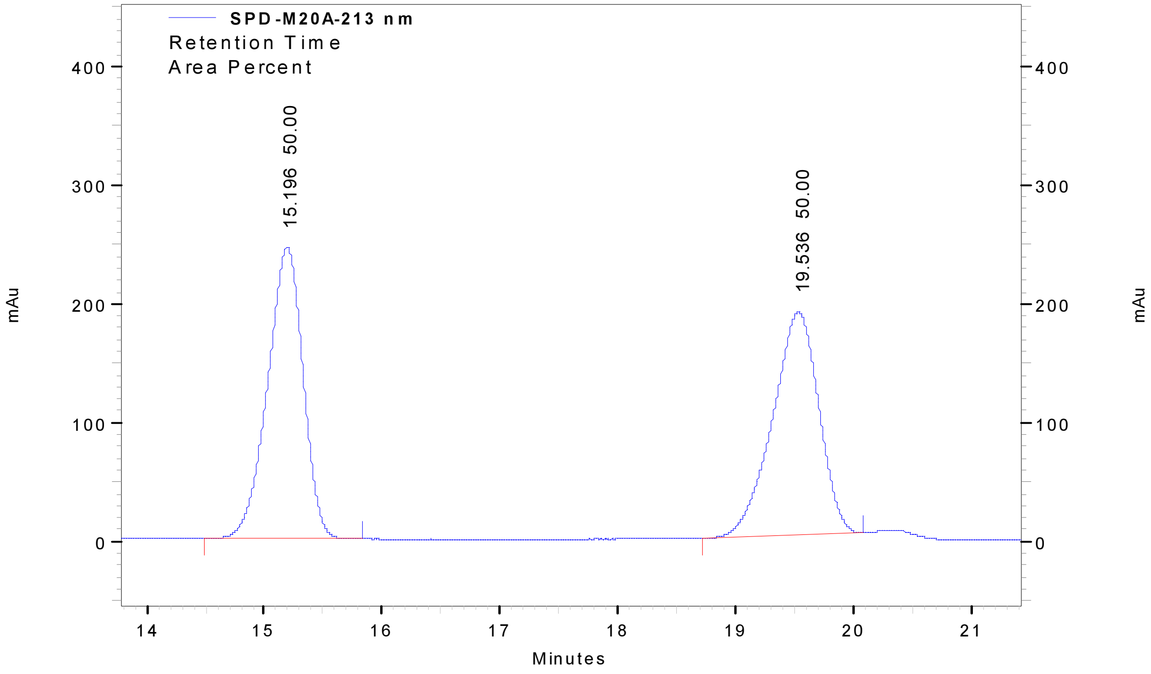

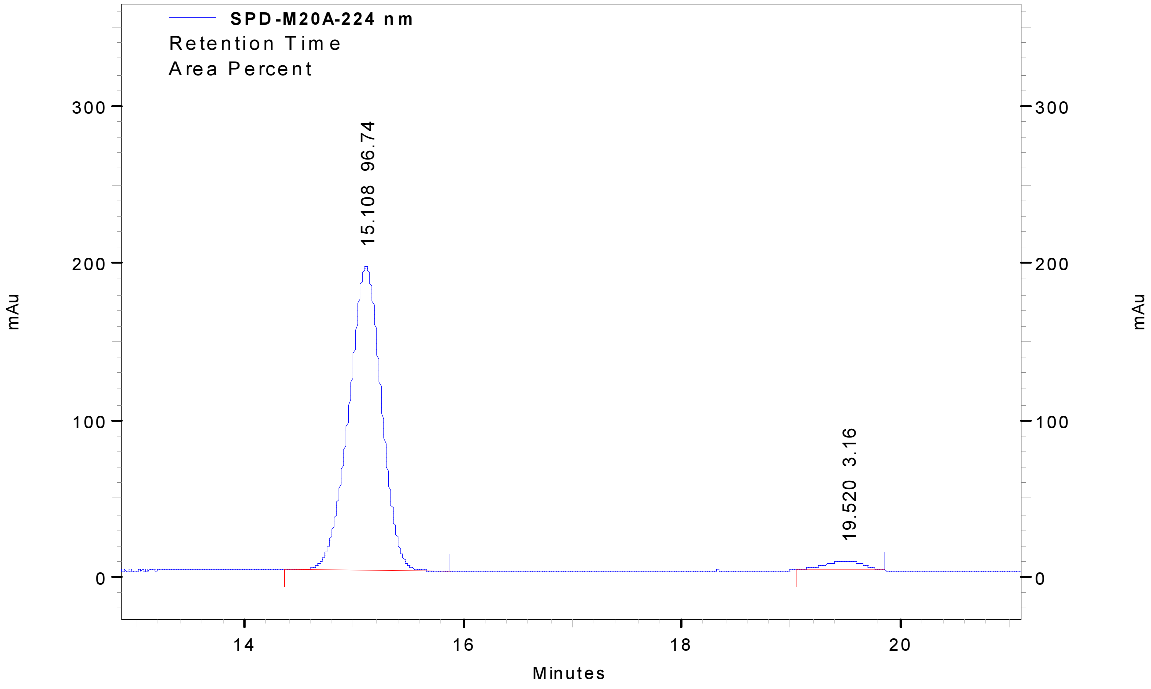

N-phenyl-*N*-(propa-1,2-dien-1-yl)acetamide

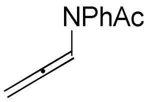

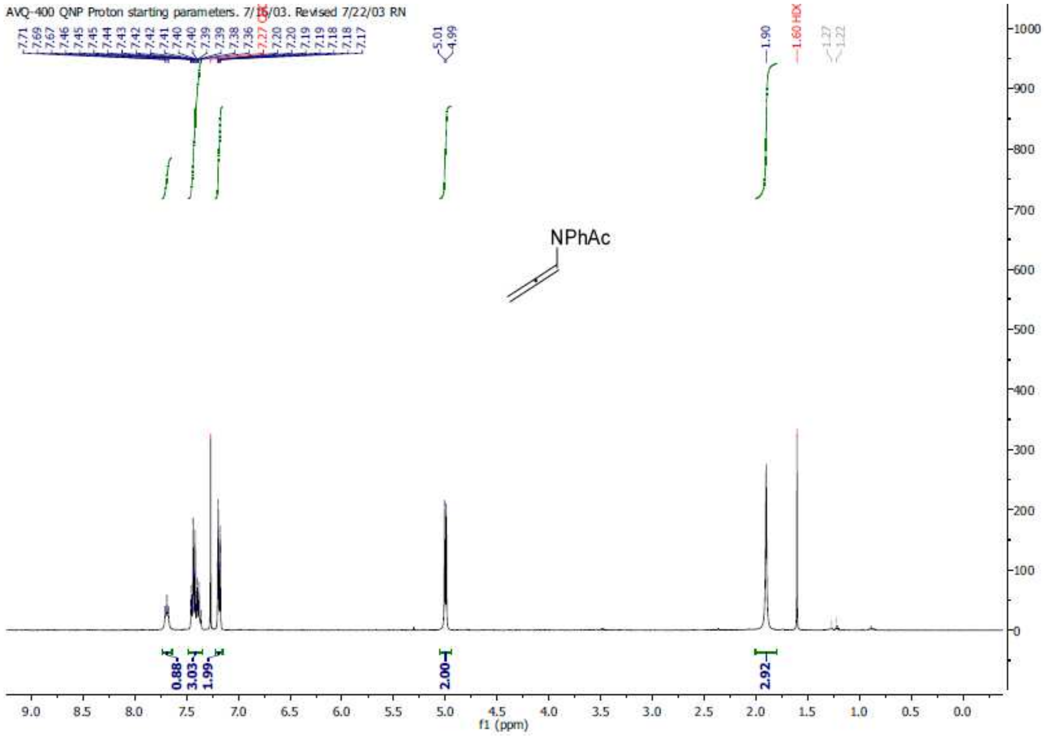

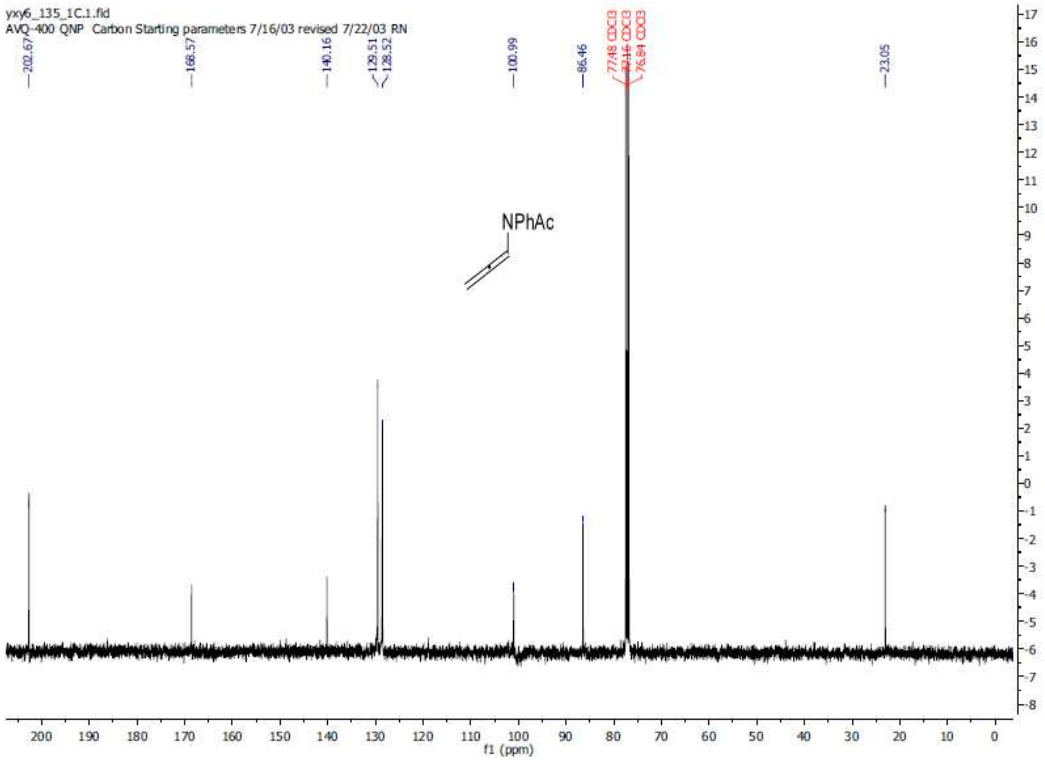

N-phenyl-*N*-(propa-1,2-dien-1-yl)benzamide

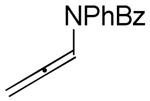

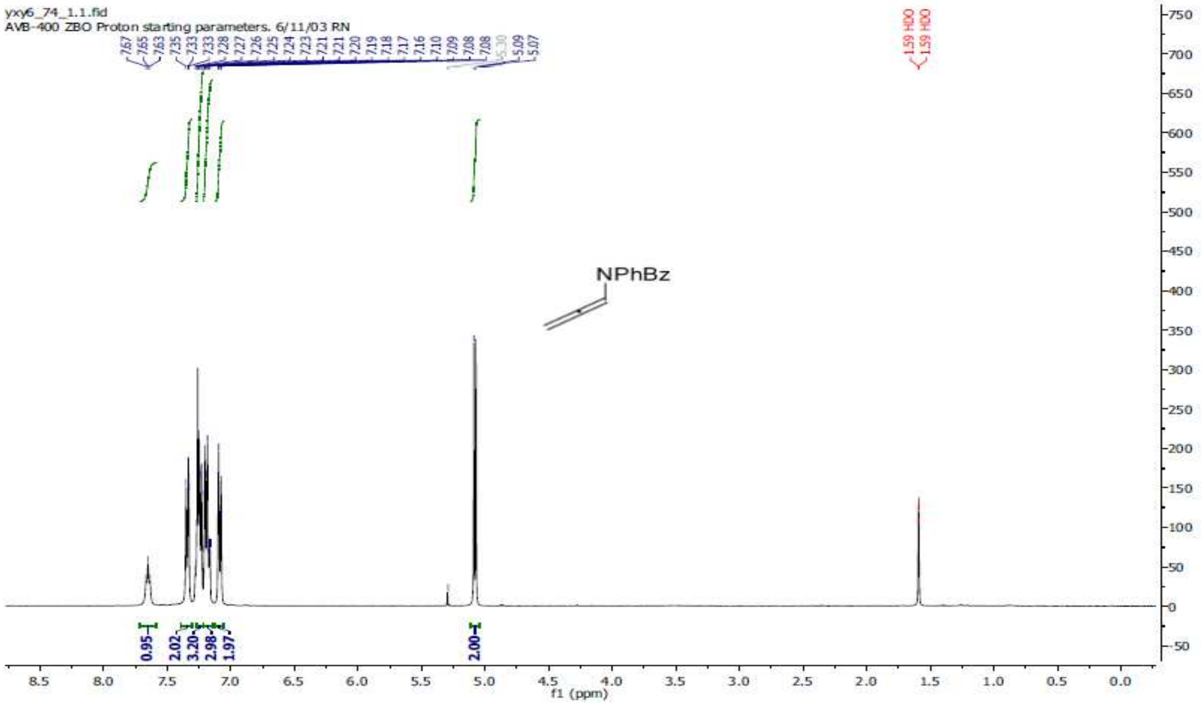

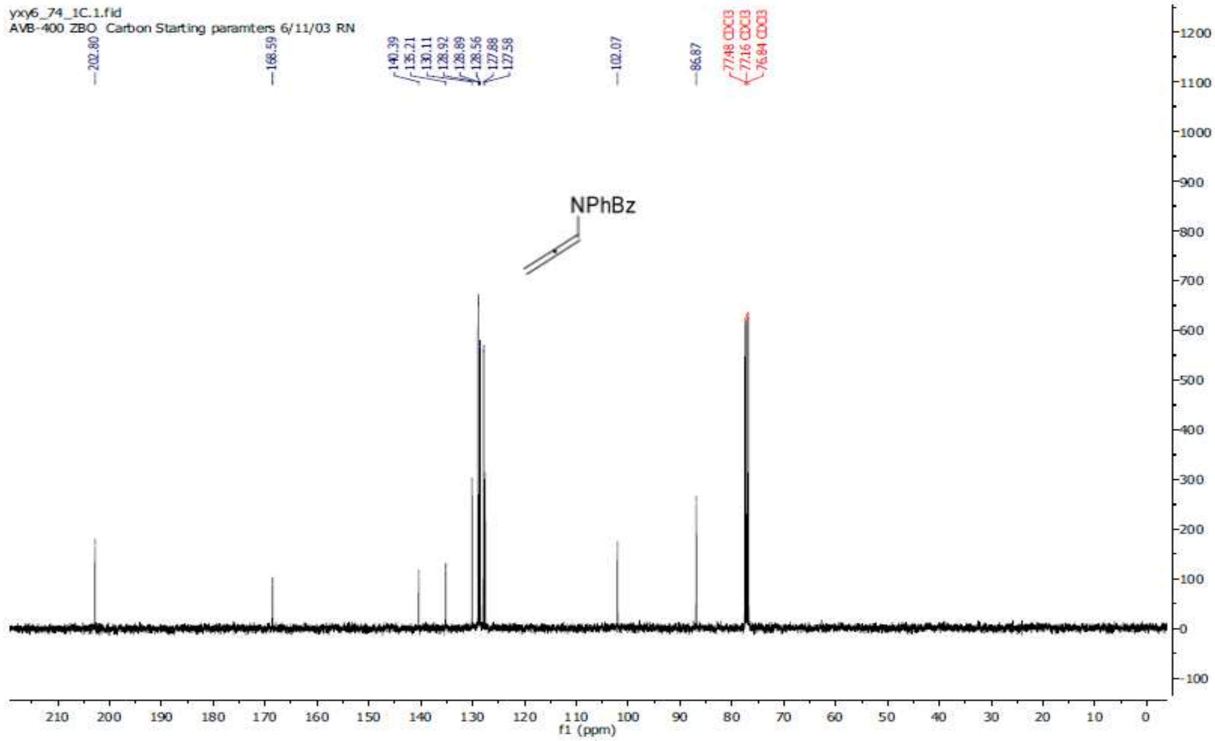

tert-butyl phenyl(propa-1,2-dien-1-yl)carbamate

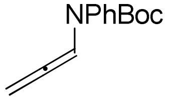

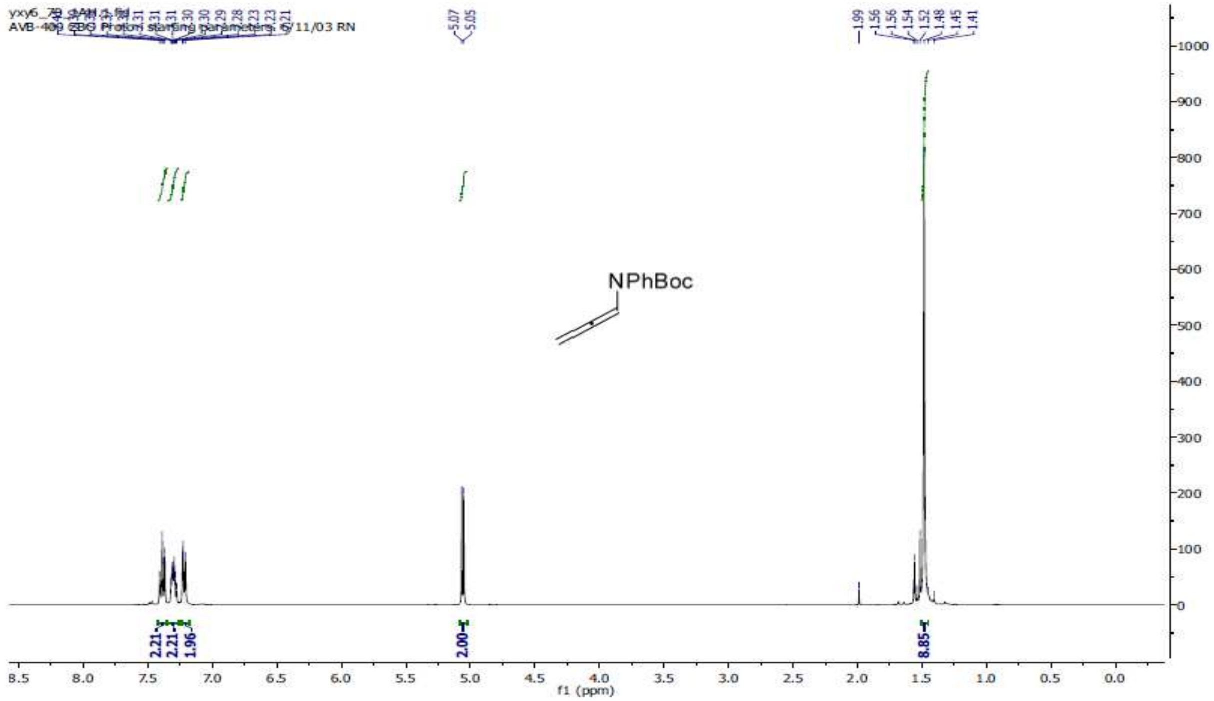

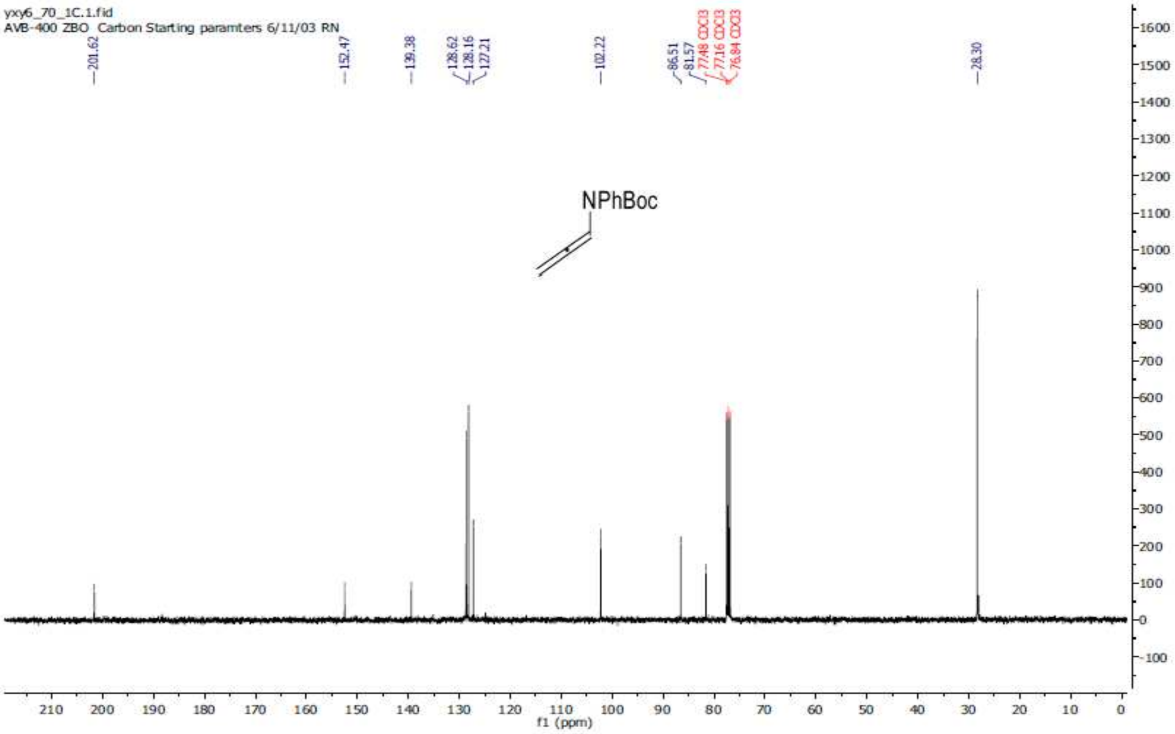

N-benzyl-*N*-(propa-1,2-dien-1-yl)benzamide

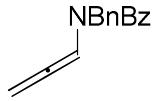

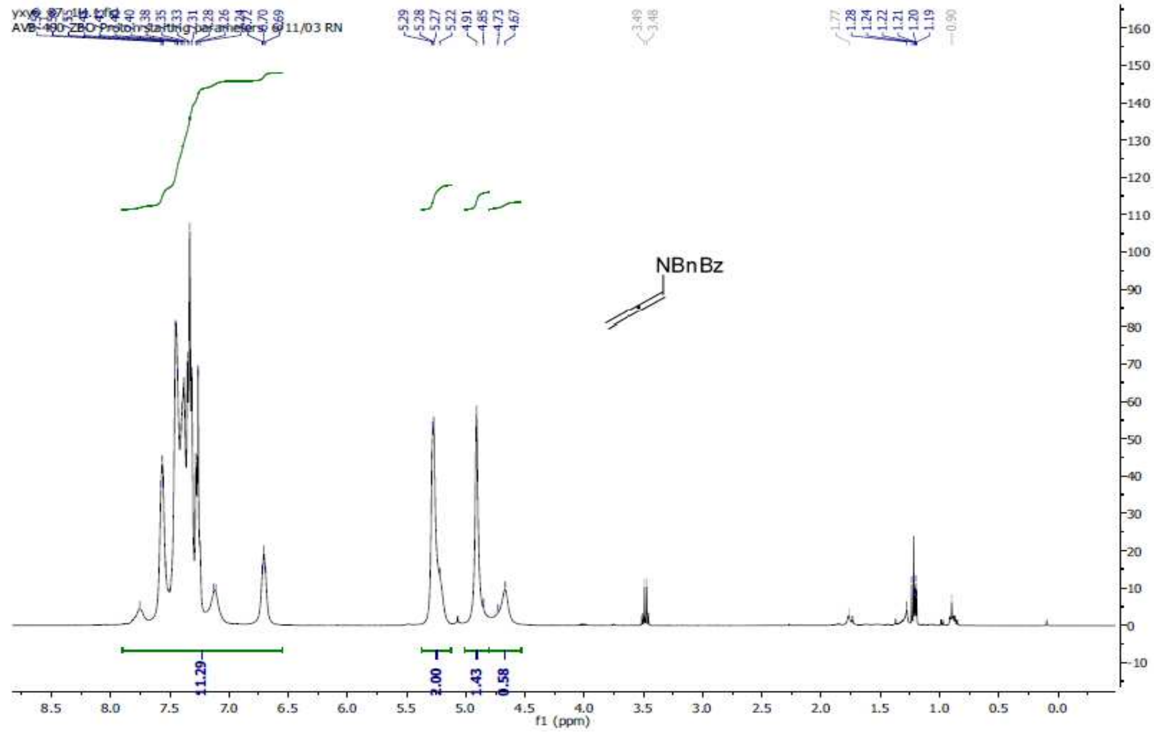

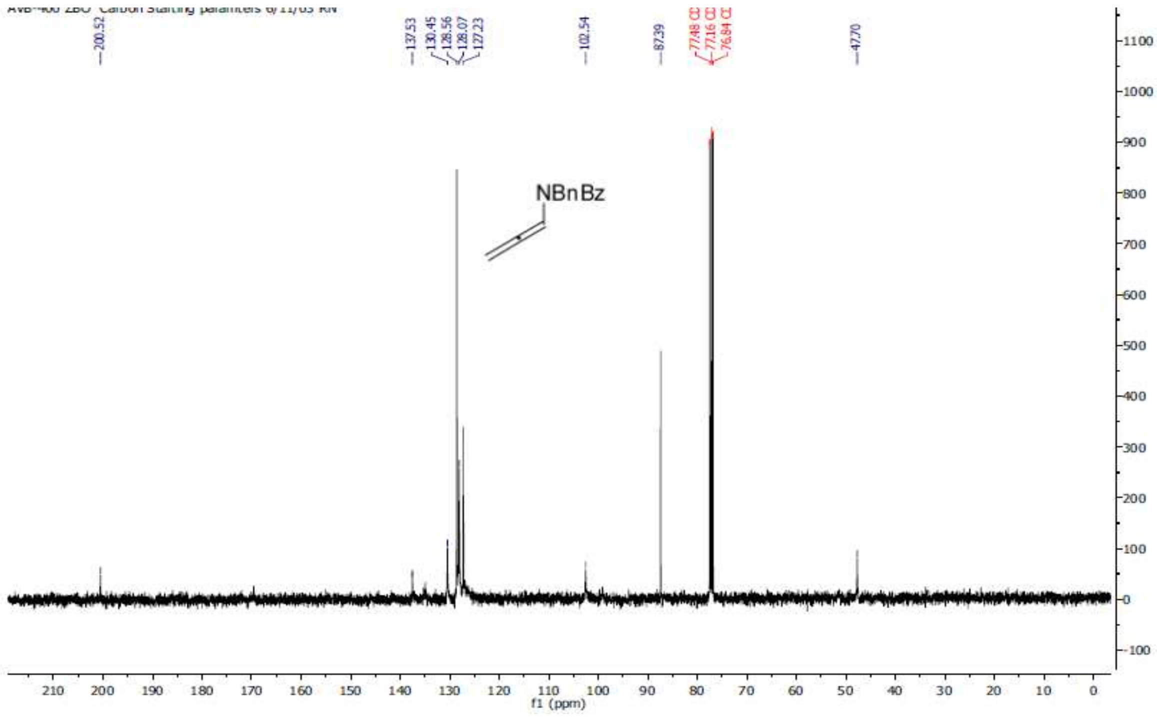

N-phenyl-*N*-(propa-1,2-dien-1-yl)isobutyramide

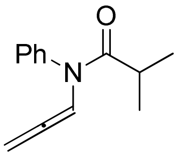

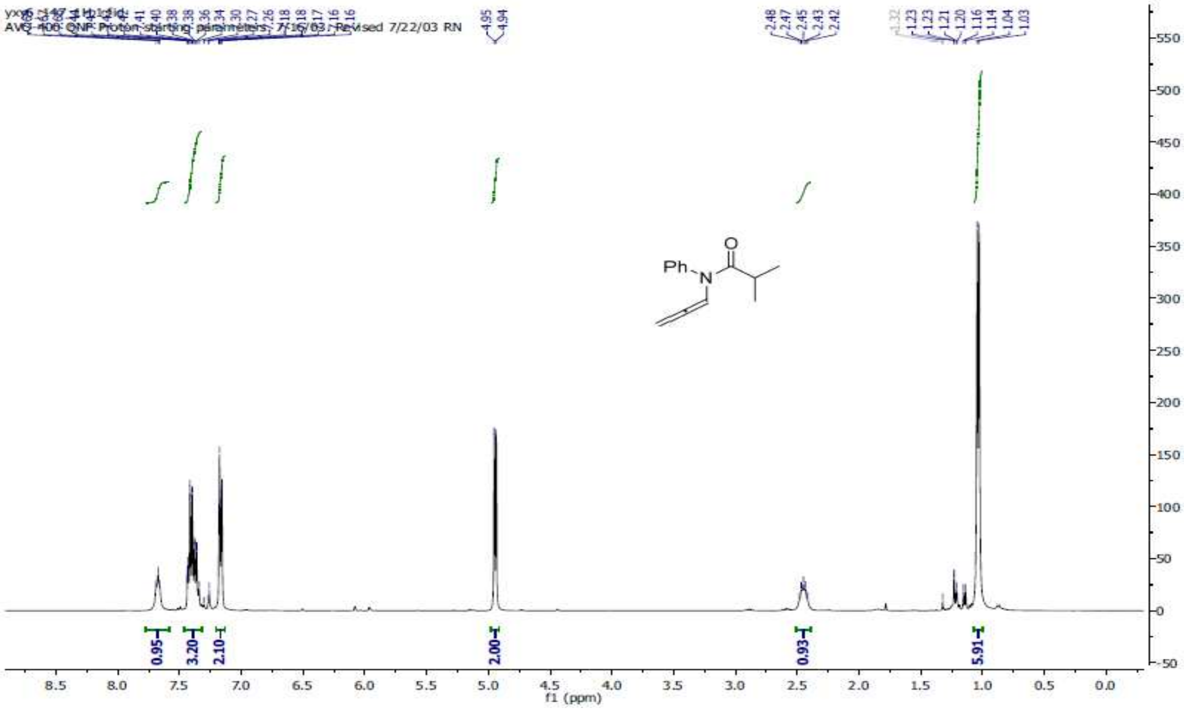

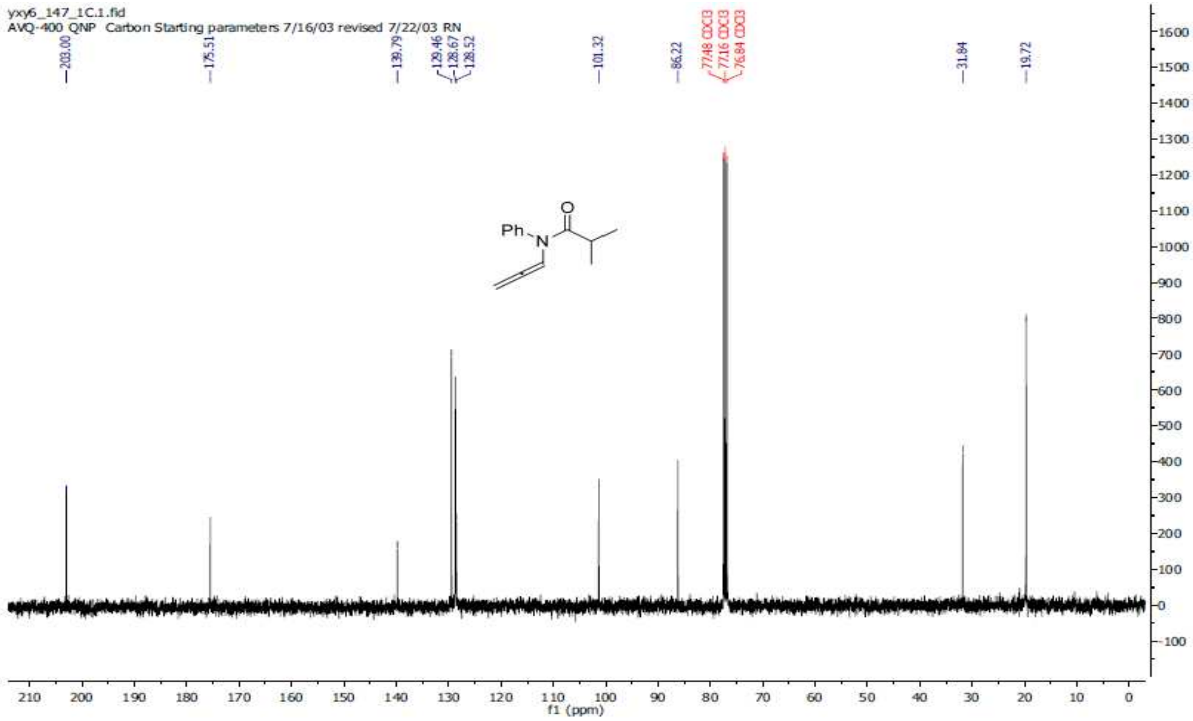

N-phenyl-*N*-(propa-1,2-dien-1-yl)pivalamide

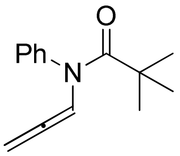

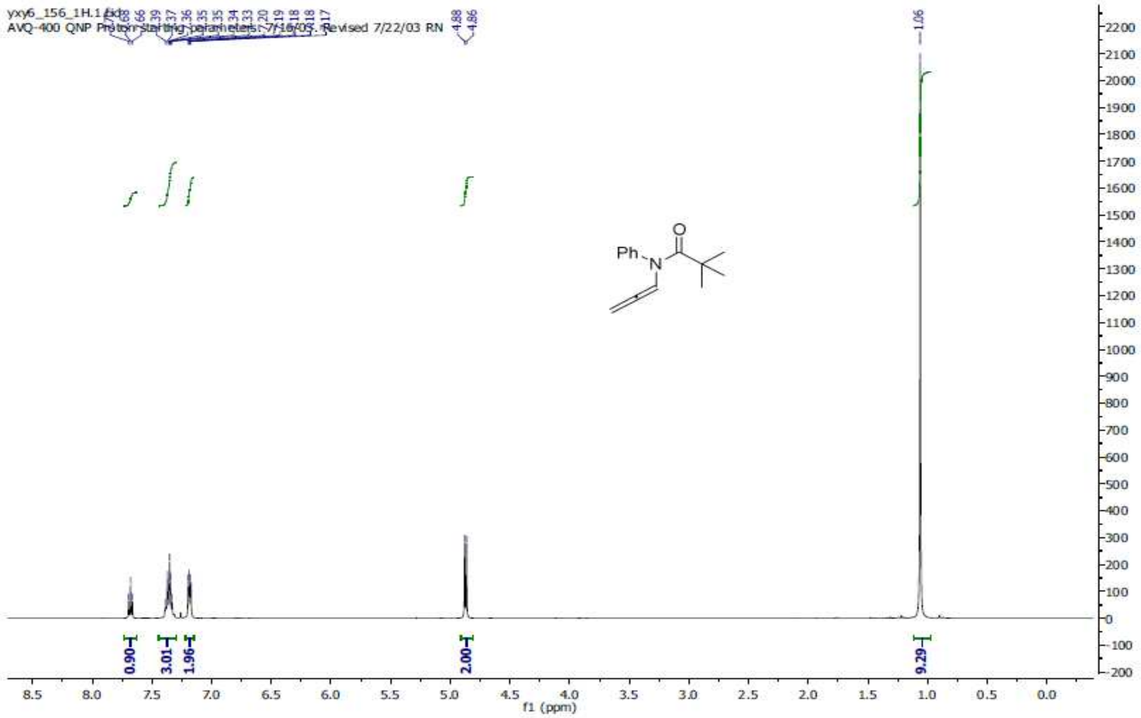

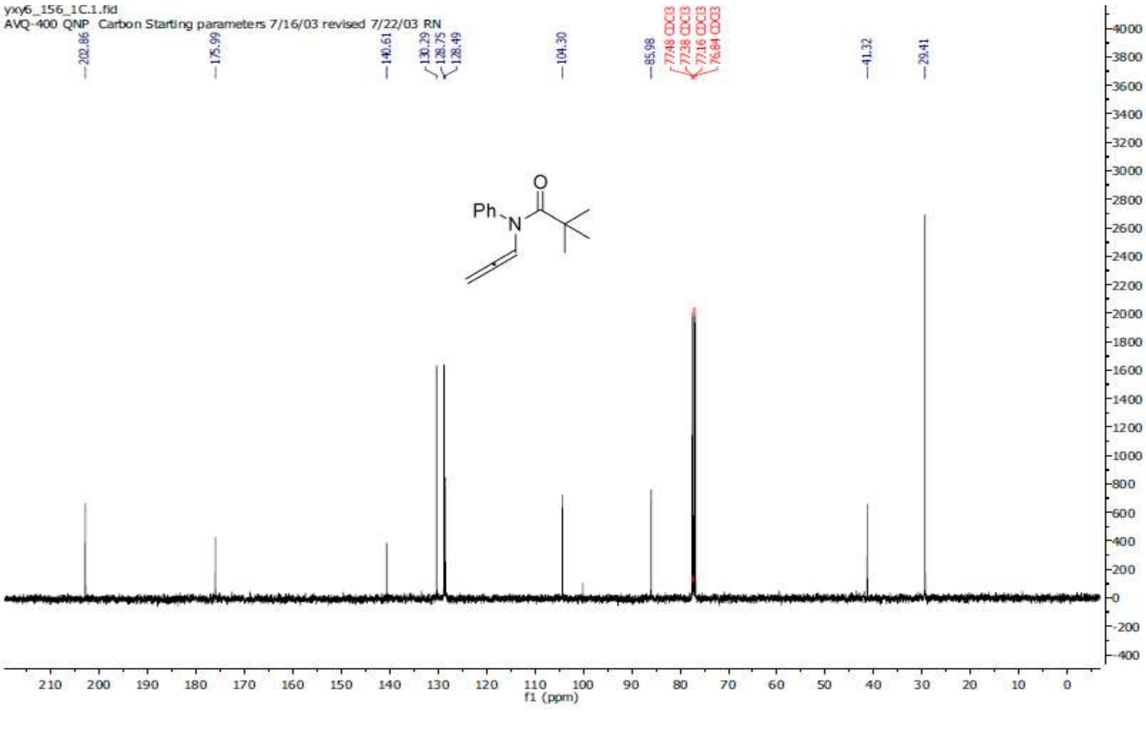

(*S,E*)-*N*-(3-(2-oxo-1-phenylcyclohexyl)prop-1-en-1-yl)-*N*-phenylbenzamide

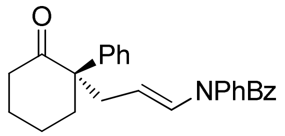

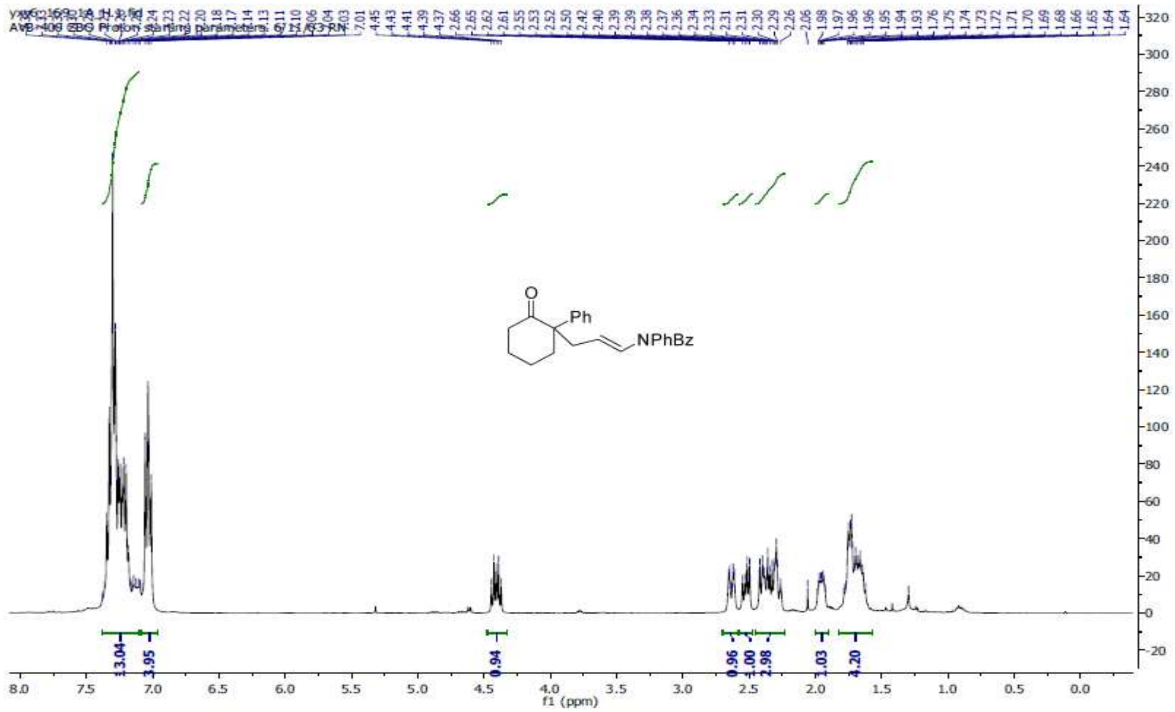

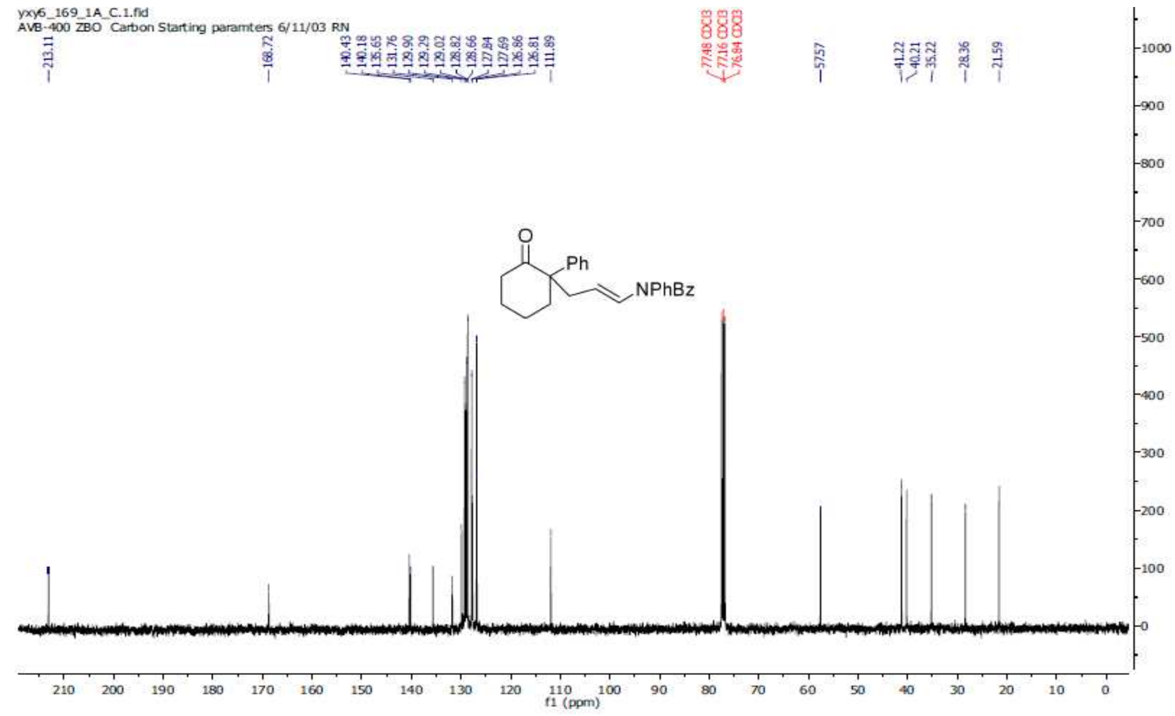

(*S*,*E*)-*N*-(3-(2-oxo-1-phenylcyclohexyl)prop-1-en-1-yl)-*N*-phenylacetamide

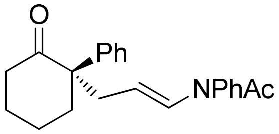

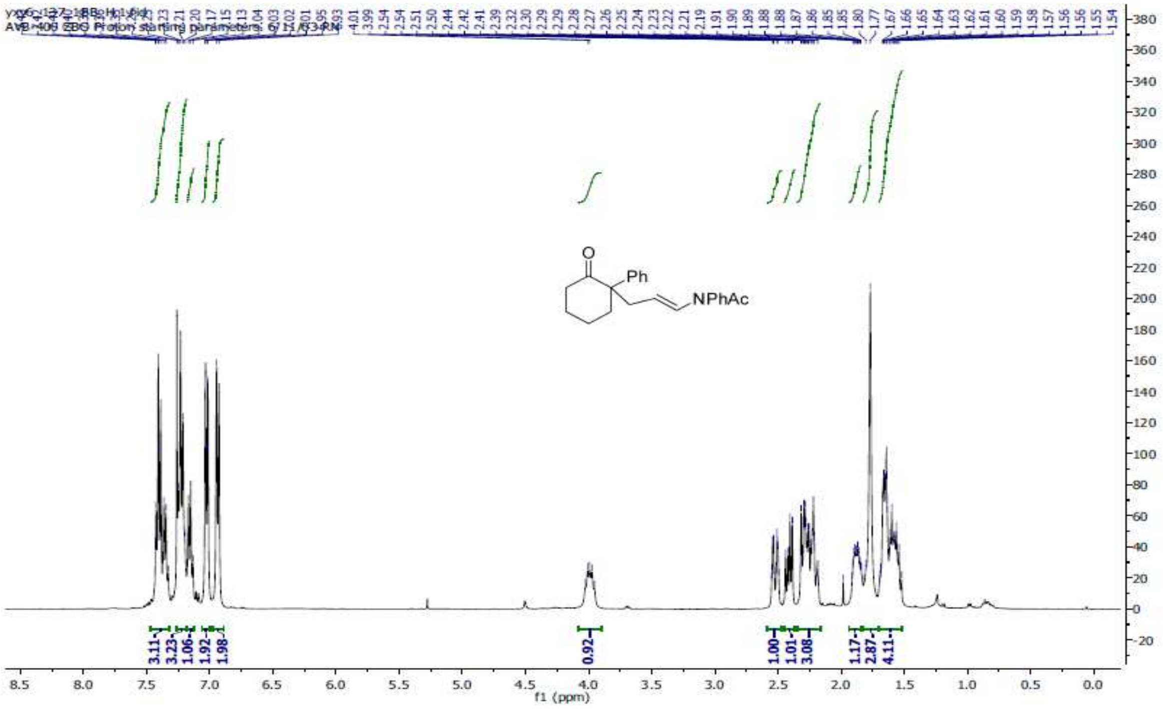

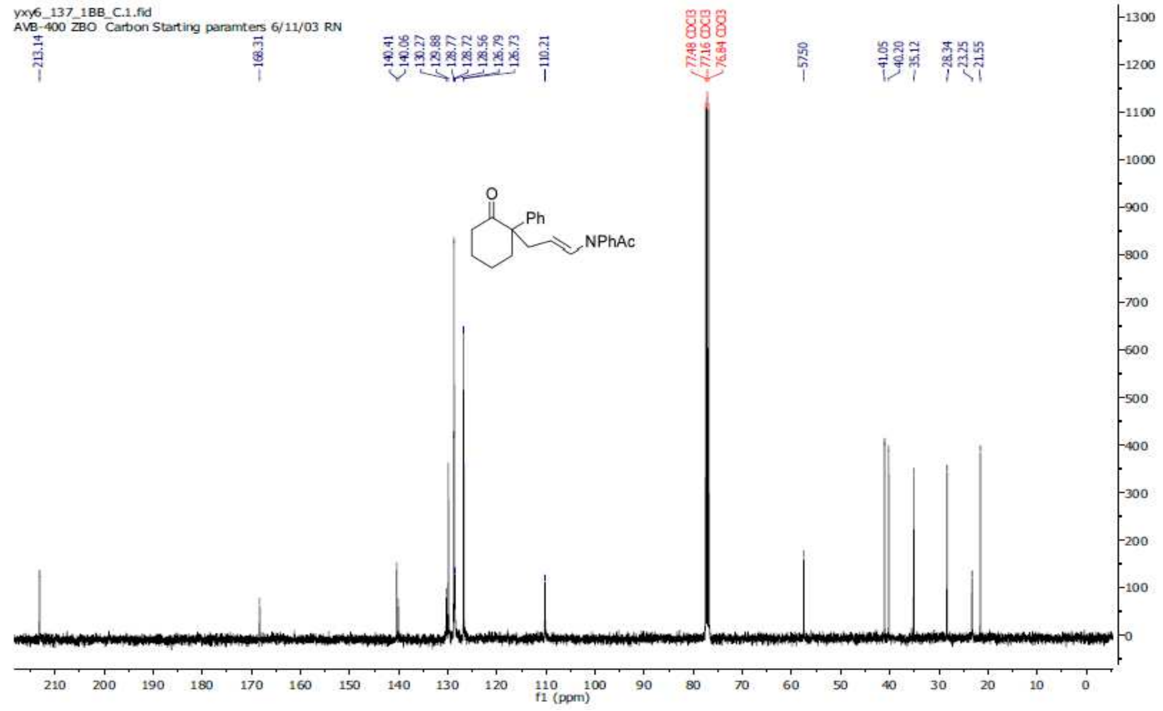

(*S*,*E*)-*N*-(3-(2-oxo-1-phenylcyclohexyl)prop-1-en-1-yl)-*N*-phenylisobutyramide

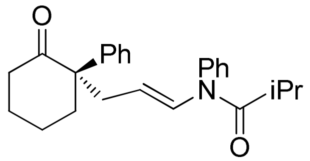

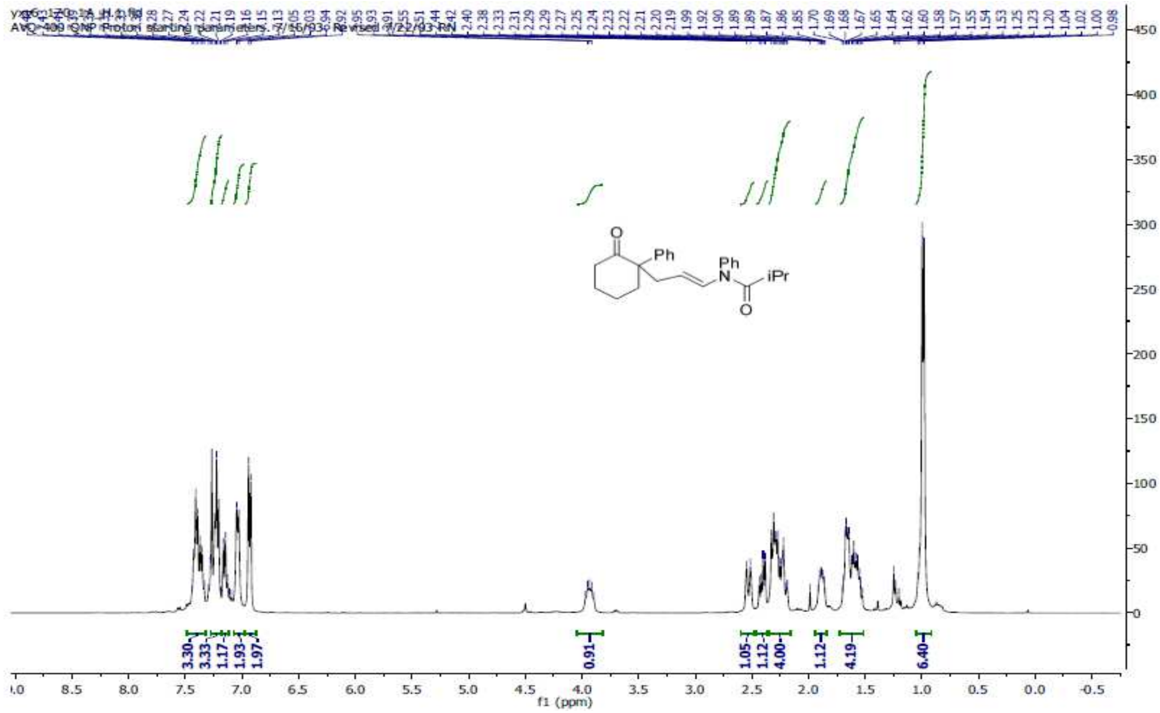

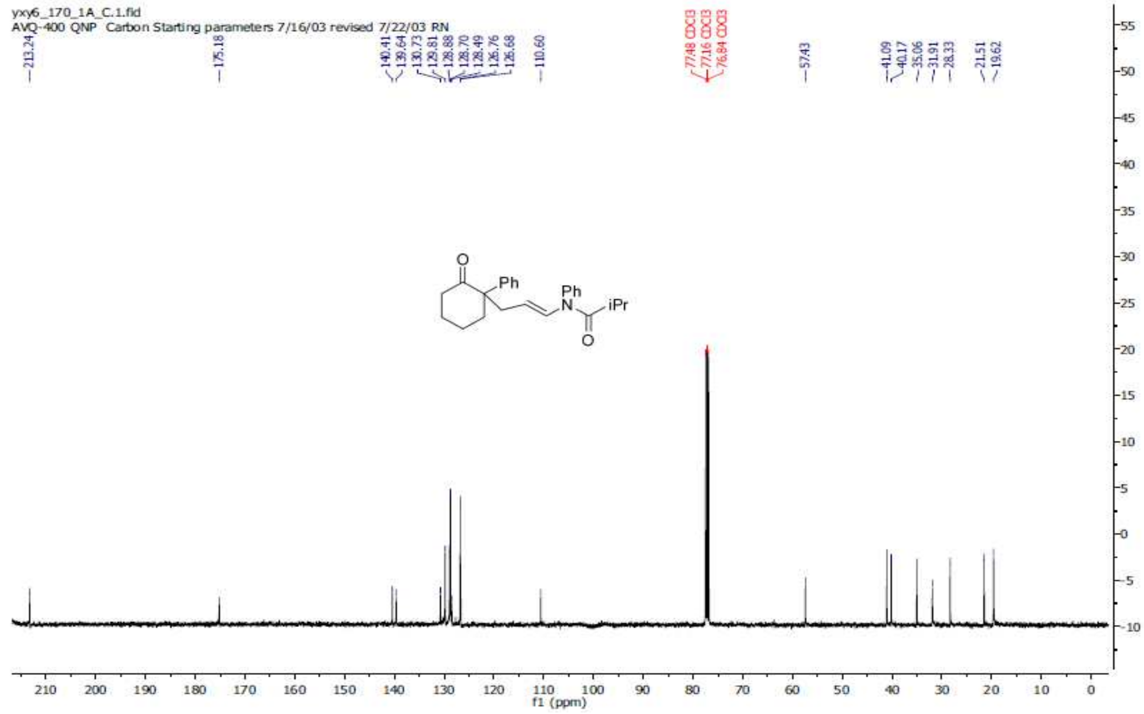

(S,E)-N-(3-(2-oxo-1-phenylcyclohexyl)prop-1-en-1-yl)-N-phenylpivalamide (**3a**)

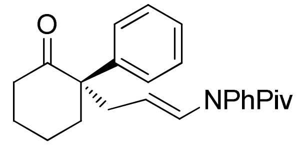

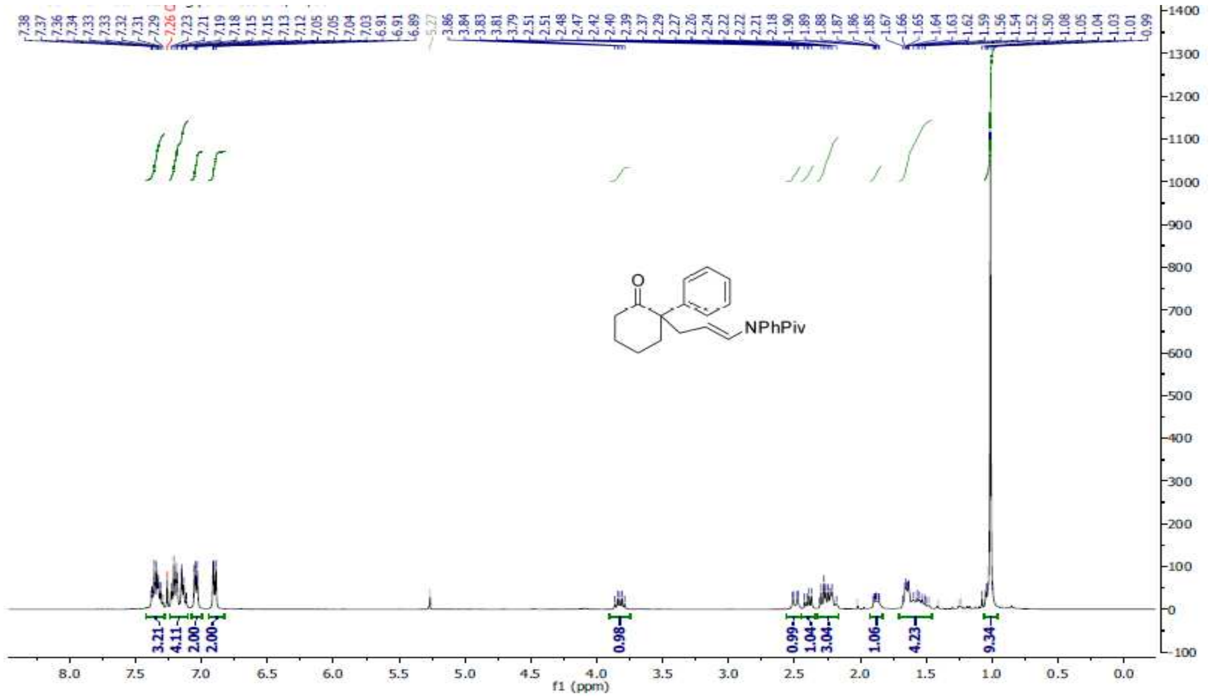

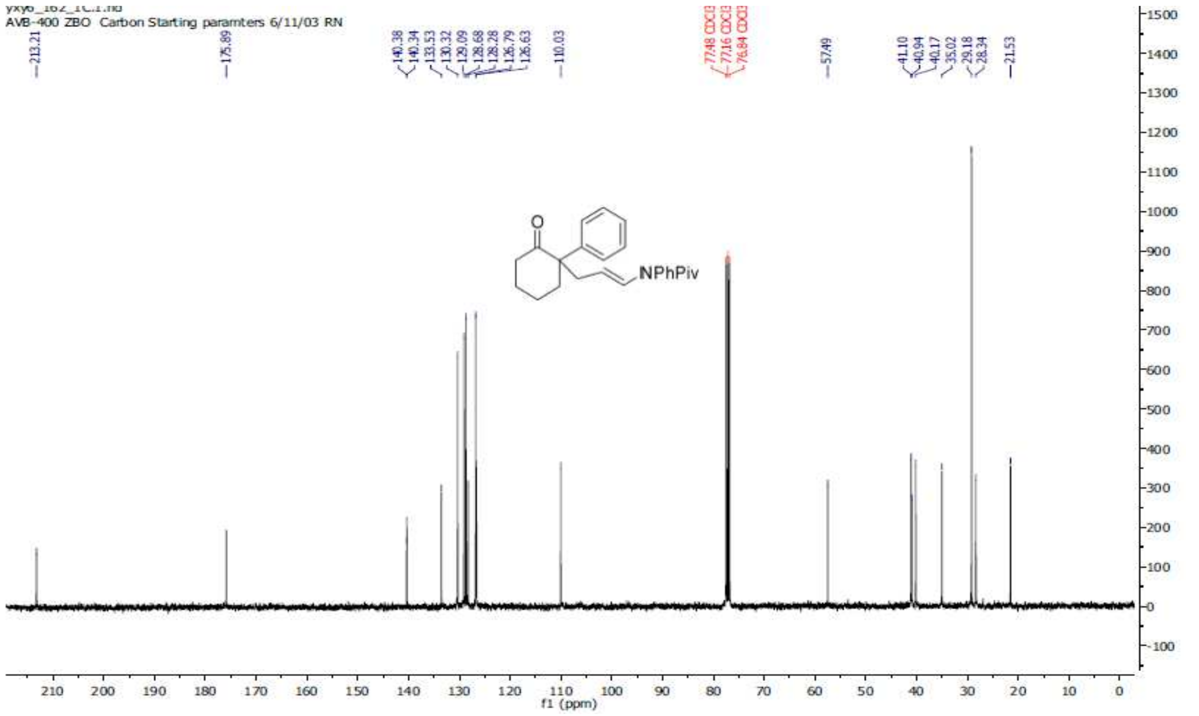

(*S*,*E*)-*N*-(3-(2-oxo-1-(p-tolyl)cyclohexyl)prop-1-en-1-yl)-*N*-phenylpivalamide (**3b**)

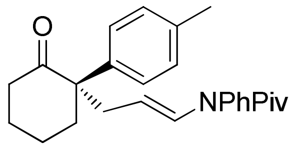

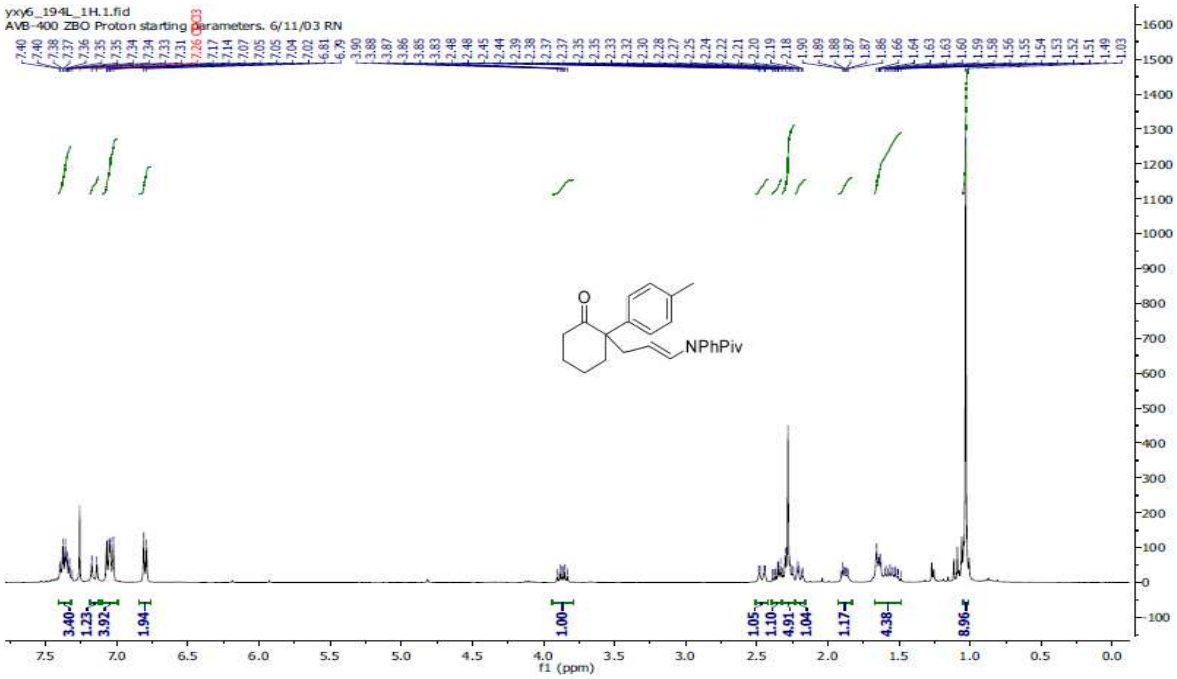

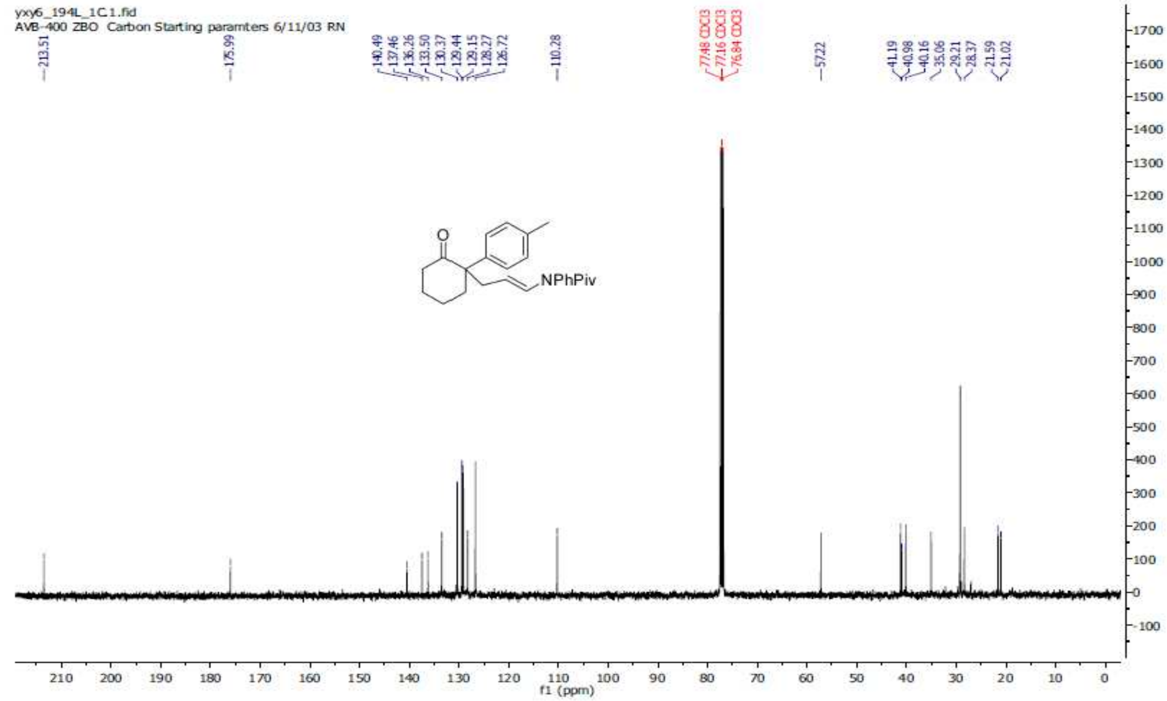

(*S,E*)-*N*-(3-(1-(naphthalen-2-yl)-2-oxocyclohexyl)prop-1-en-1-yl)-*N*-phenylpivalamide (**3c**)

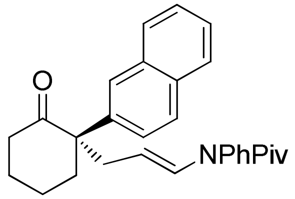

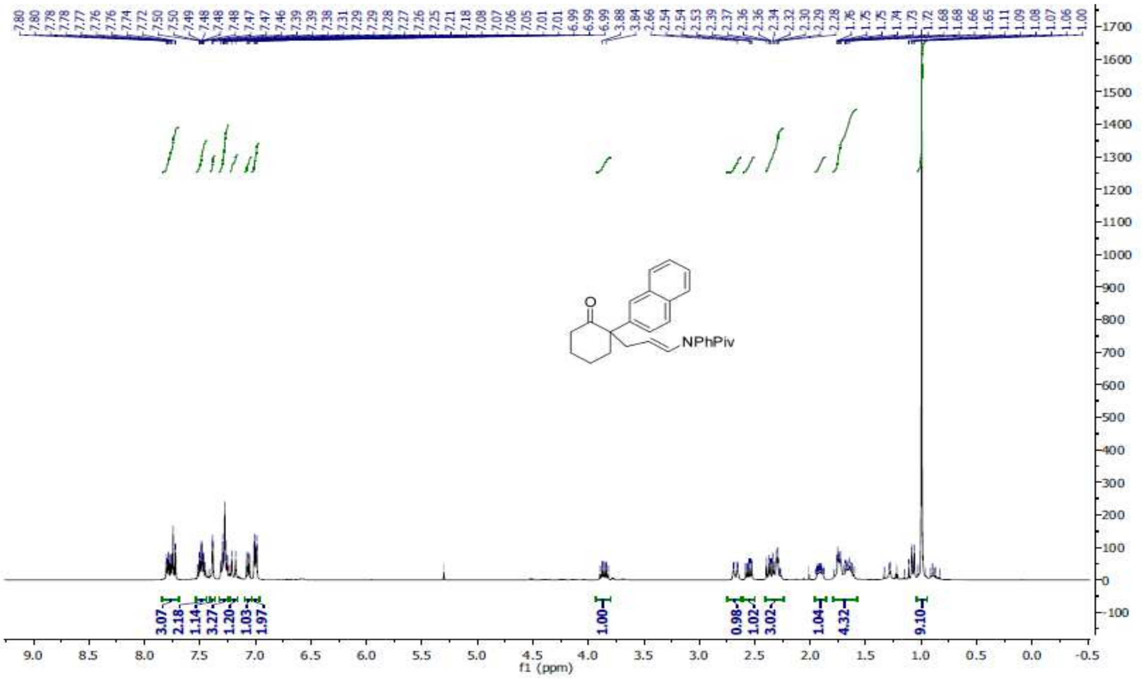

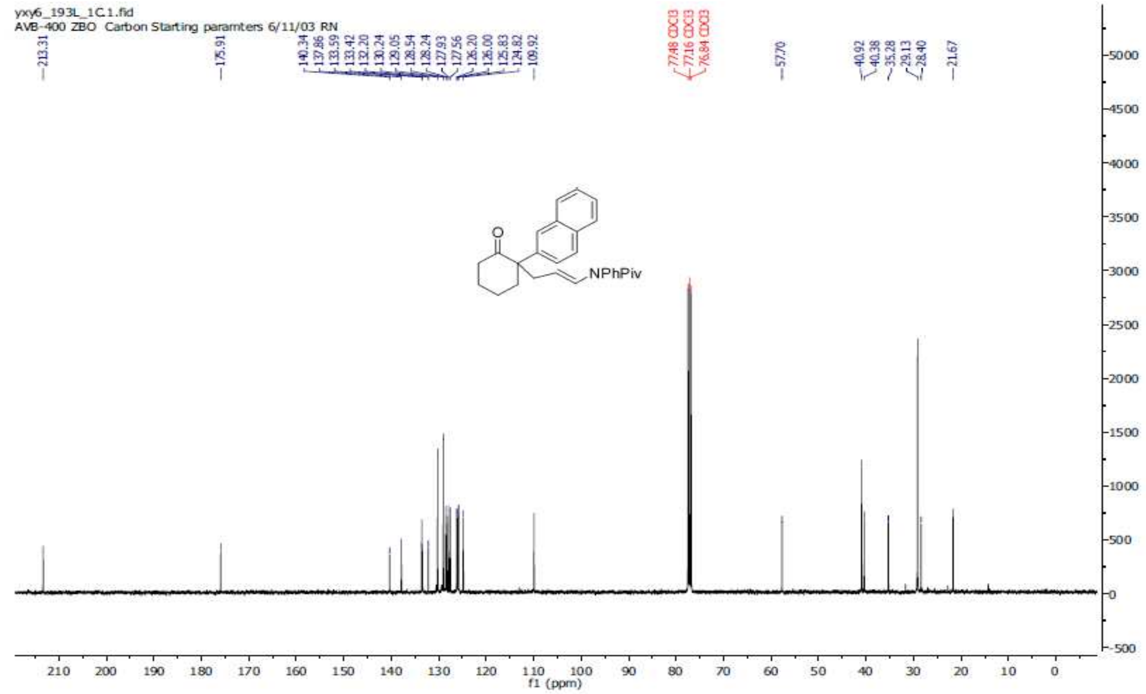

(*S*,*E*)-*N*-(3-(1-(3-bromophenyl)-2-oxocyclohexyl)prop-1-en-1-yl)-*N*-phenylpivalamide (**3d**)

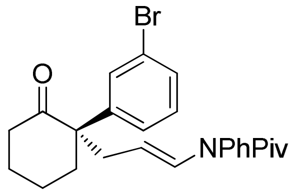

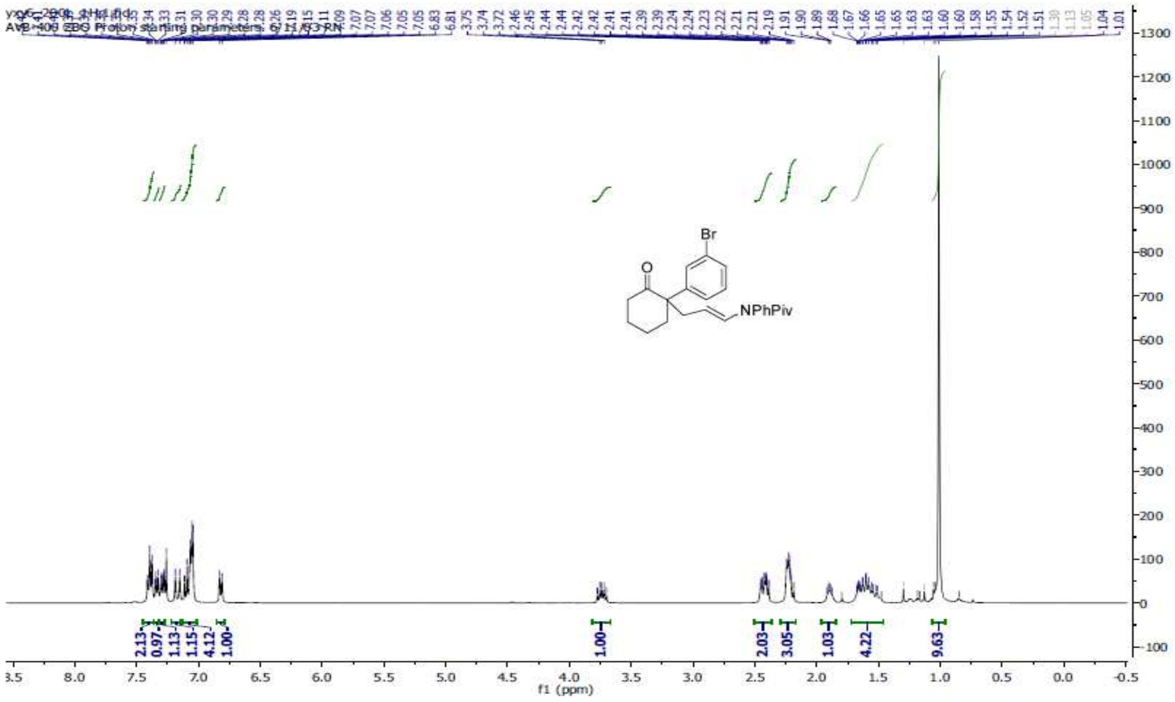

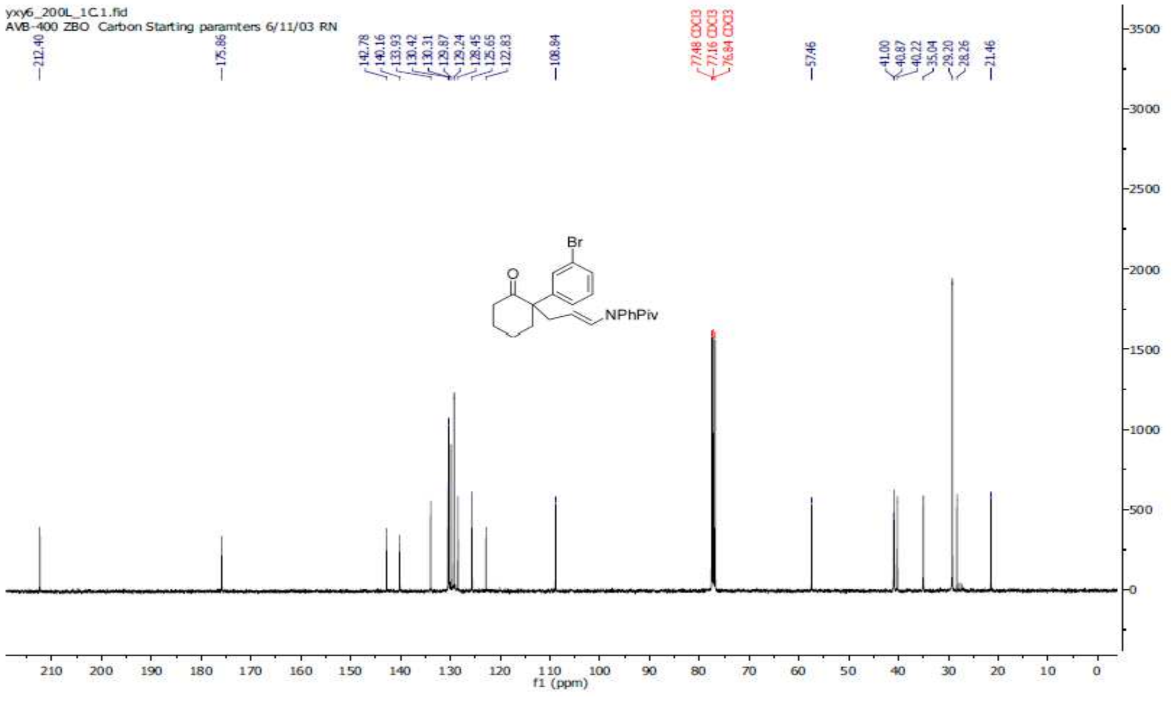

(*S*,*E*)-*N*-(3-(2-oxo-1-(4-(trifluoromethyl)phenyl)cyclohexyl)prop-1-en-1-yl)-*N*-phenylpivalamide (**3e**)

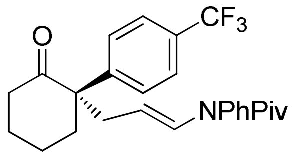

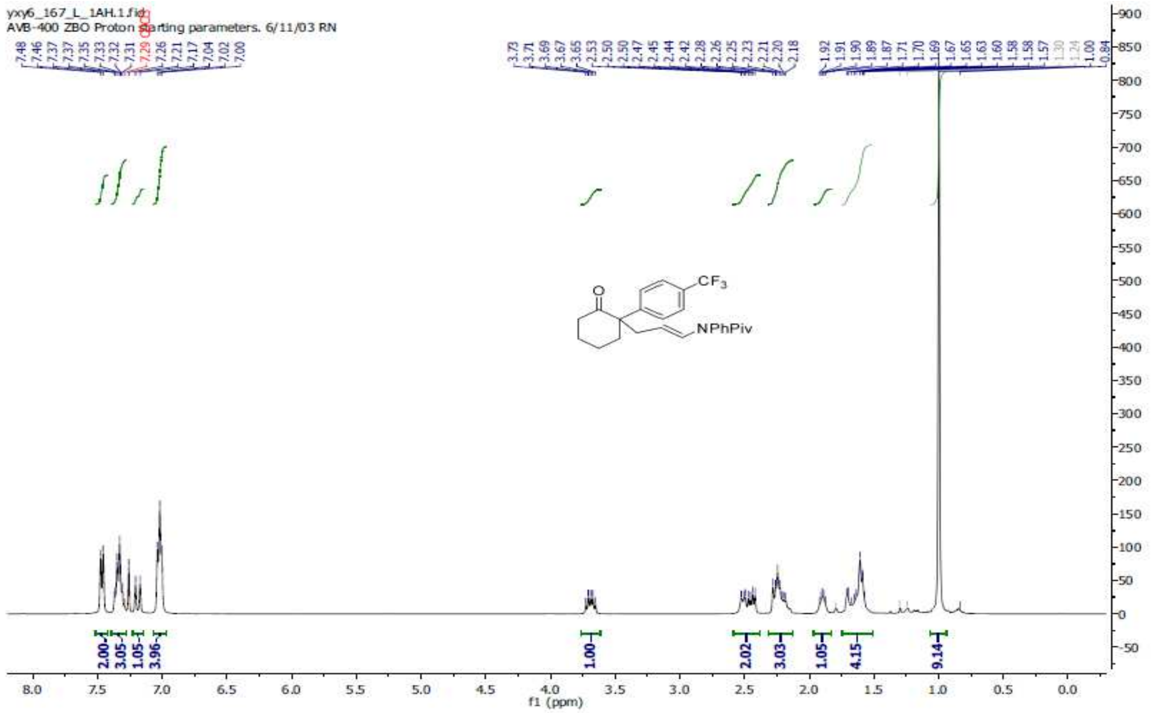

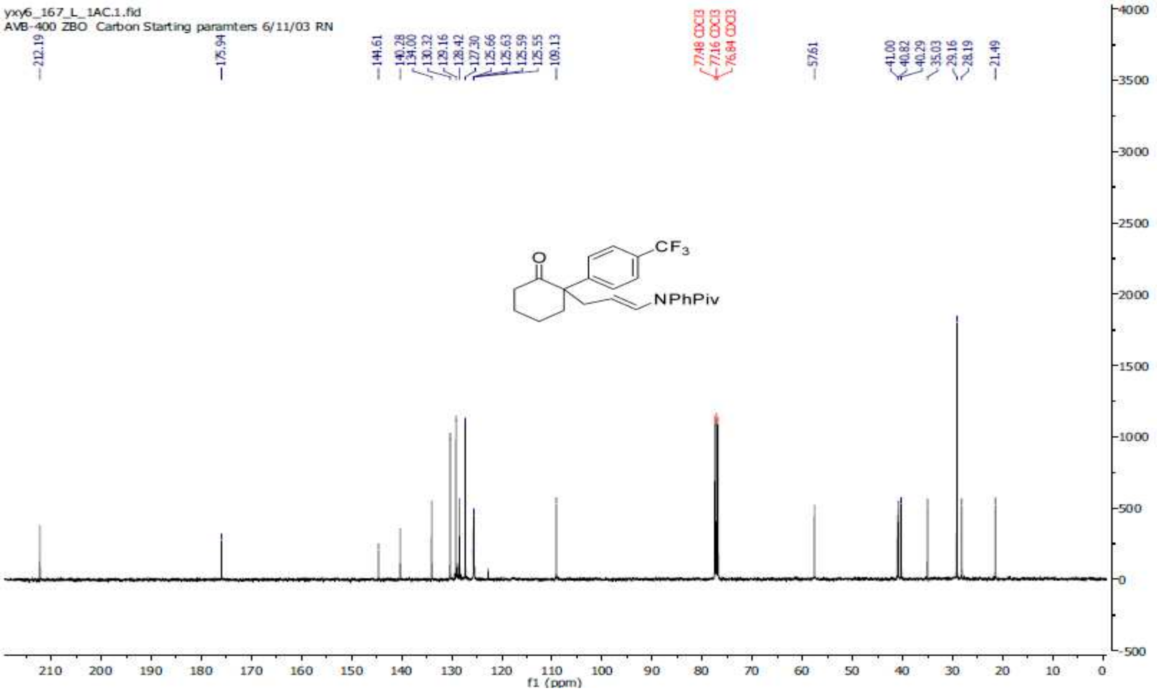

(*S,E*)-tert-butyl 4-(2-oxo-1-(3-(*N*-phenylpivalamido)allyl)cyclohexyl)benzoate (**3f**)

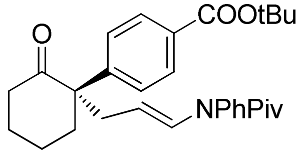

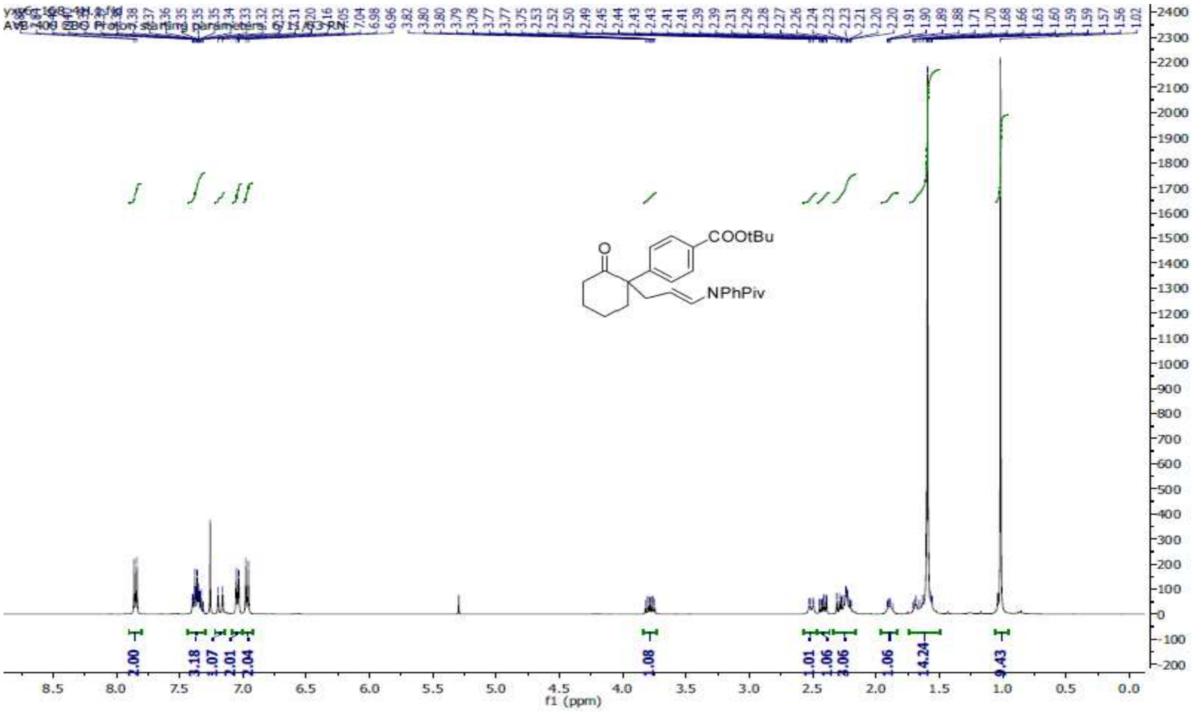

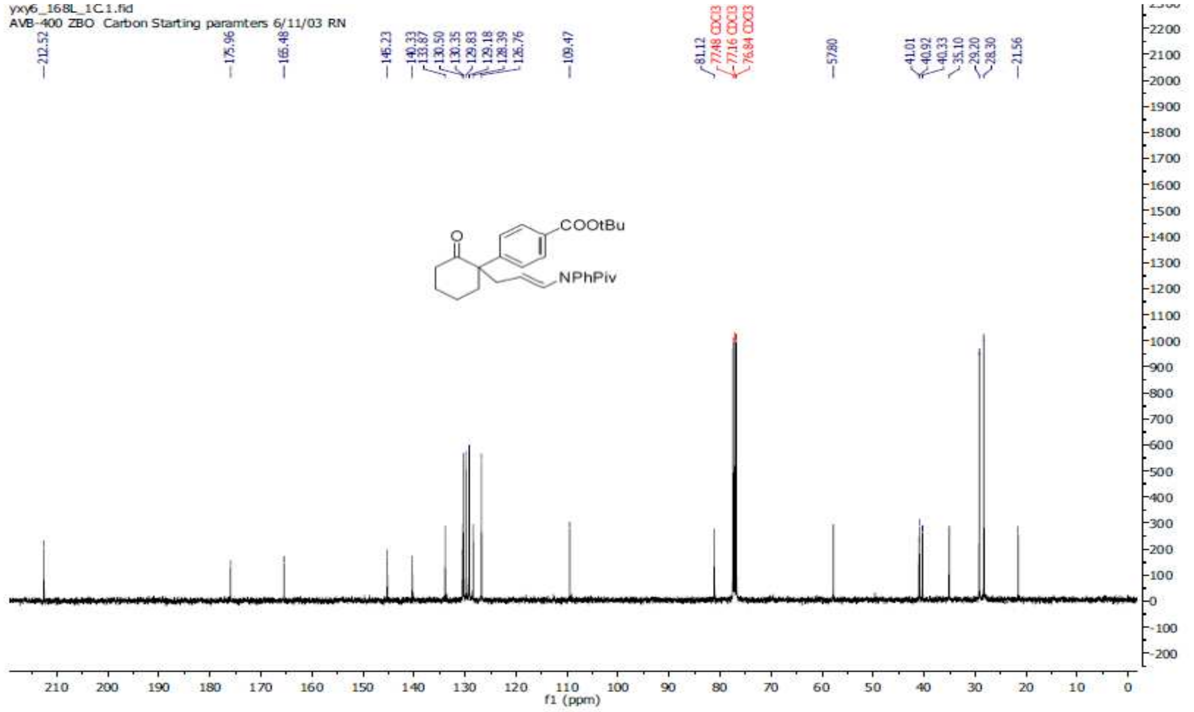

(*S*,*E*)-*N*-(3-(1-(benzo[d][1,3]dioxol-5-yl)-2-oxocyclohexyl)prop-1-en-1-yl)-*N*-phenylpivalamide (**3g**)

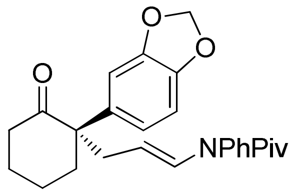

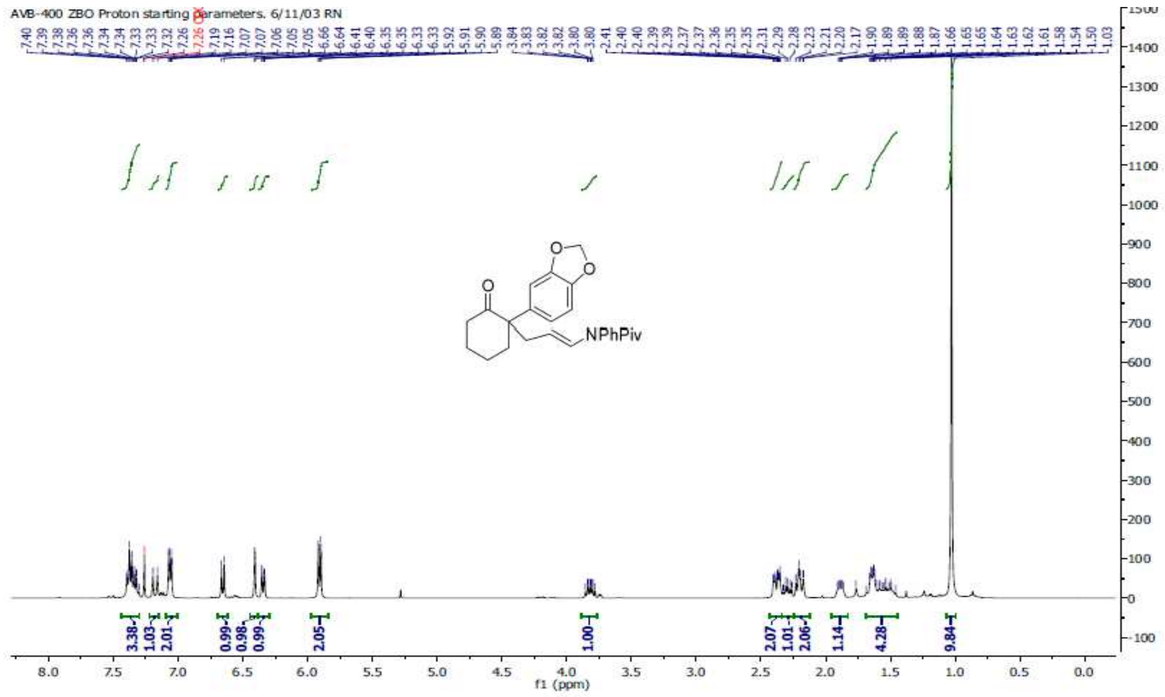

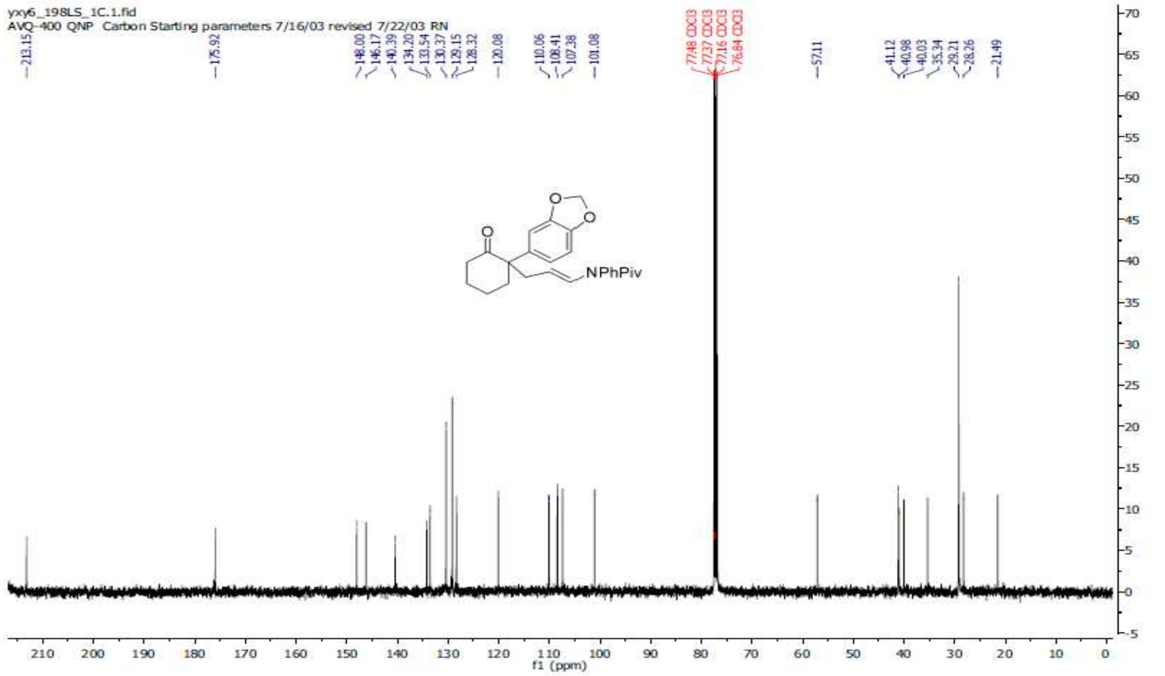

(*S,E*)-*N*-(3-(1-(3-methoxyphenyl)-2-oxocyclohexyl)prop-1-en-1-yl)-*N*-phenylpivalamide (**3h**)

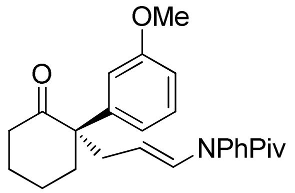

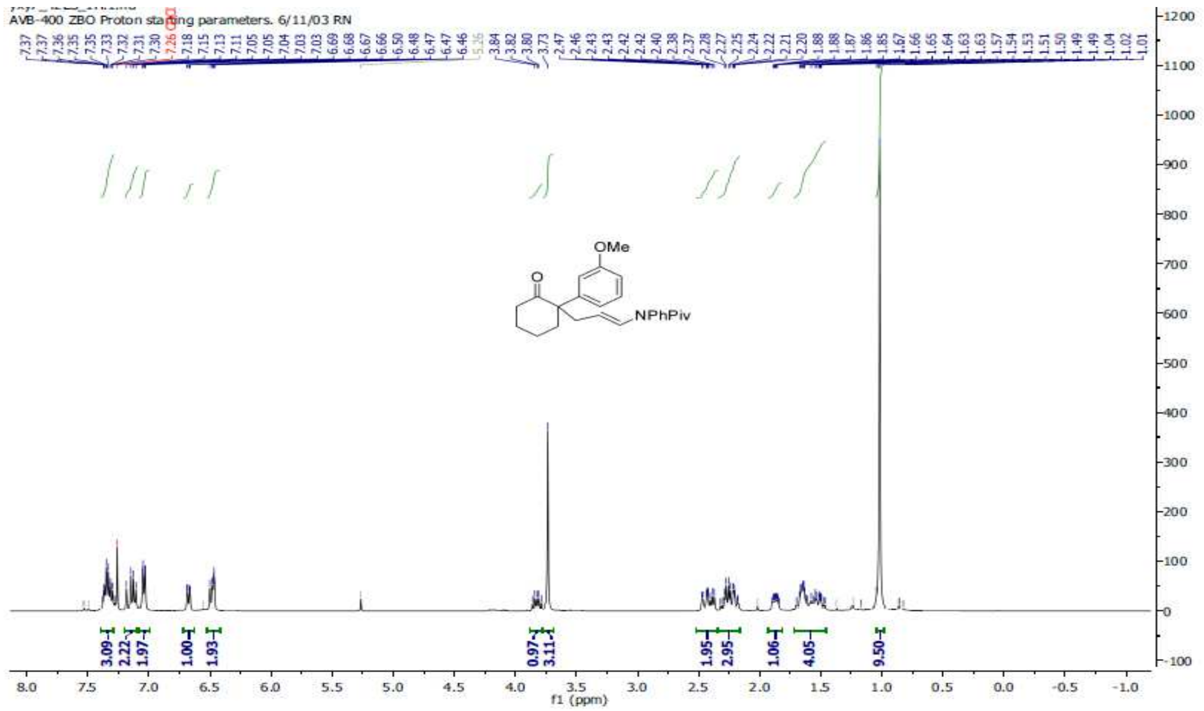

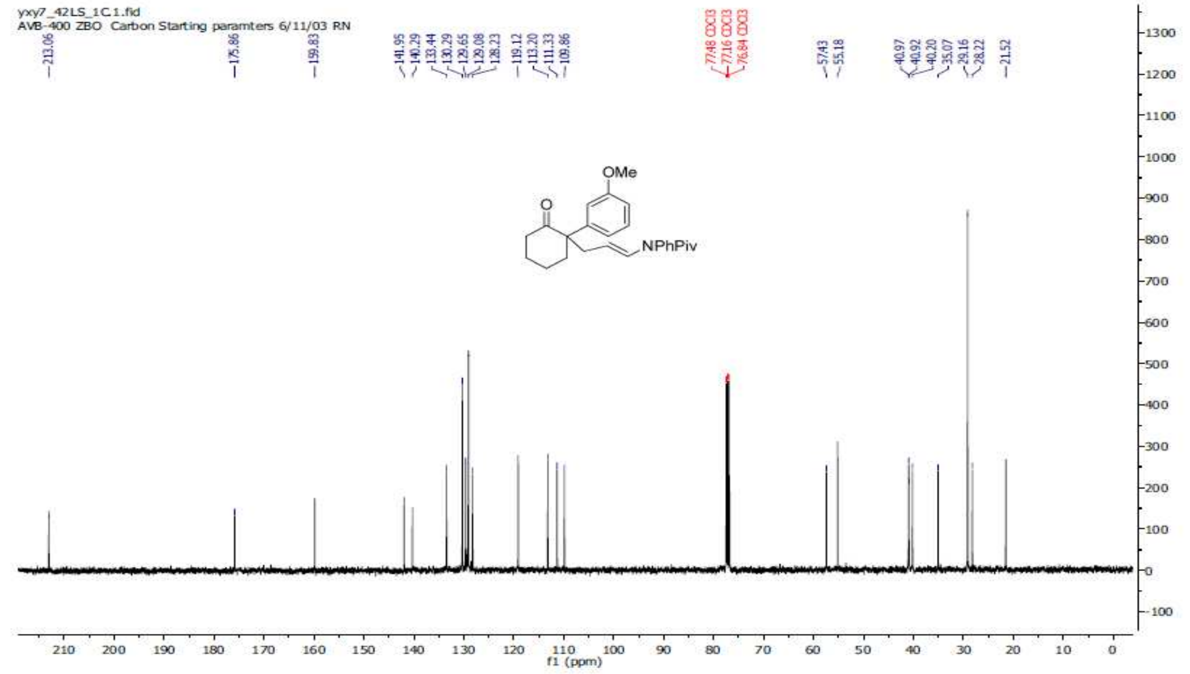

(*S,E*)-*N*-(3-(2-oxo-1-(o-tolyl)cyclohexyl)prop-1-en-1-yl)-*N*-phenylpivalamide (**3i**)

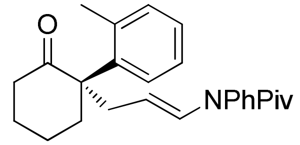

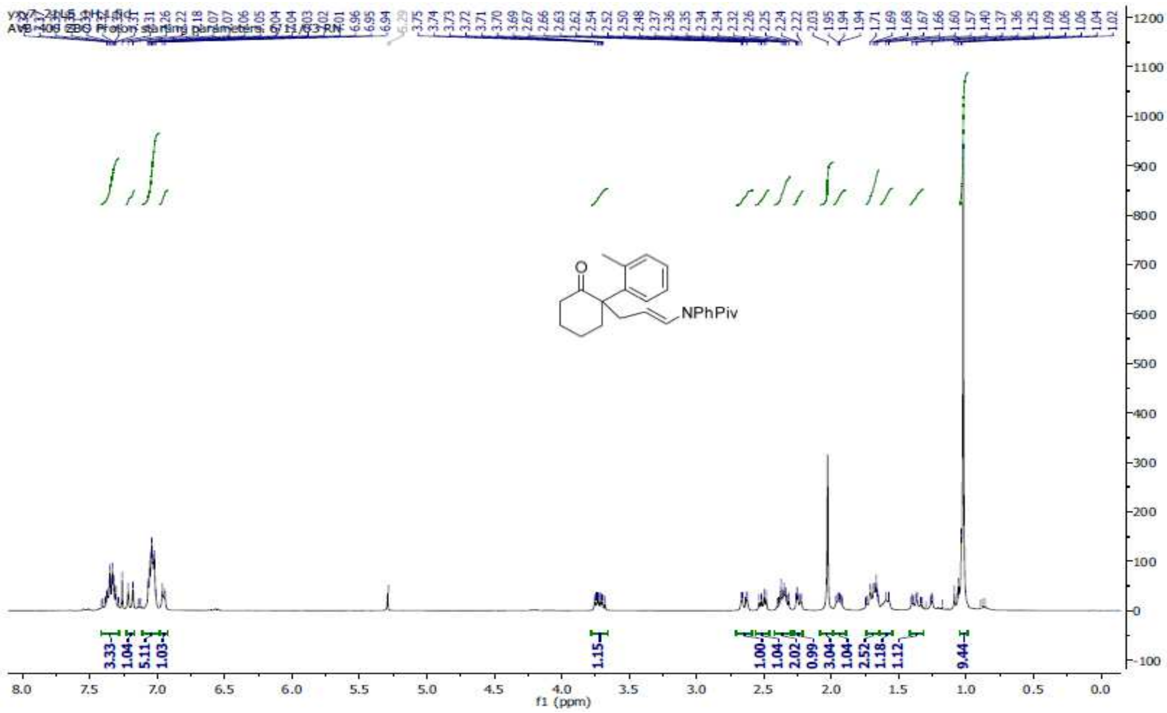

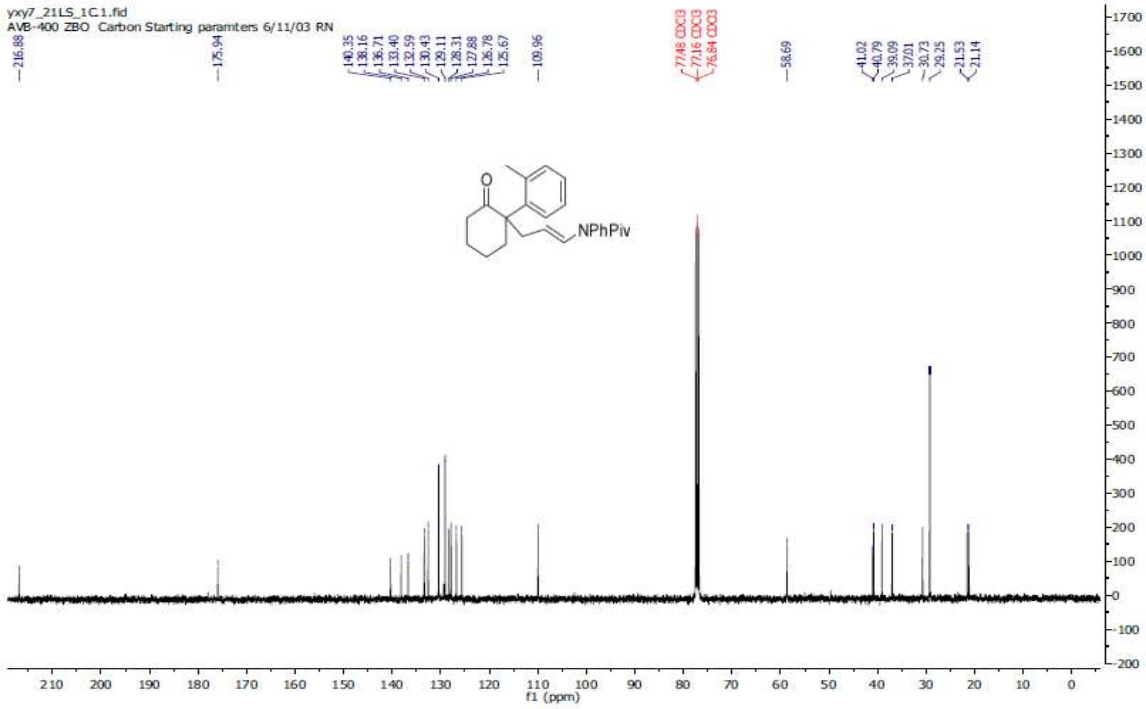

(*R,E*)-*N*-(3-(2-oxo-1-(thiophen-2-yl)cyclohexyl)prop-1-en-1-yl)-*N*-phenylpivalamide (**3j**)

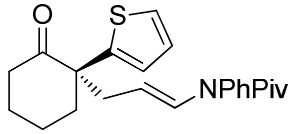

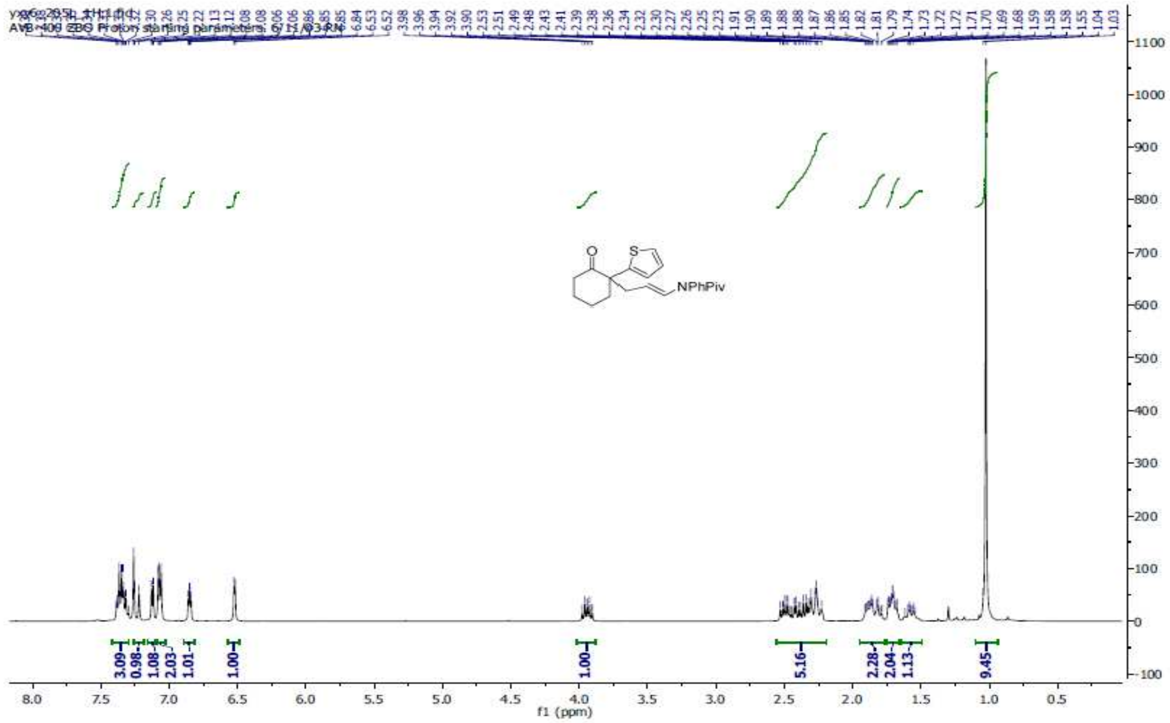

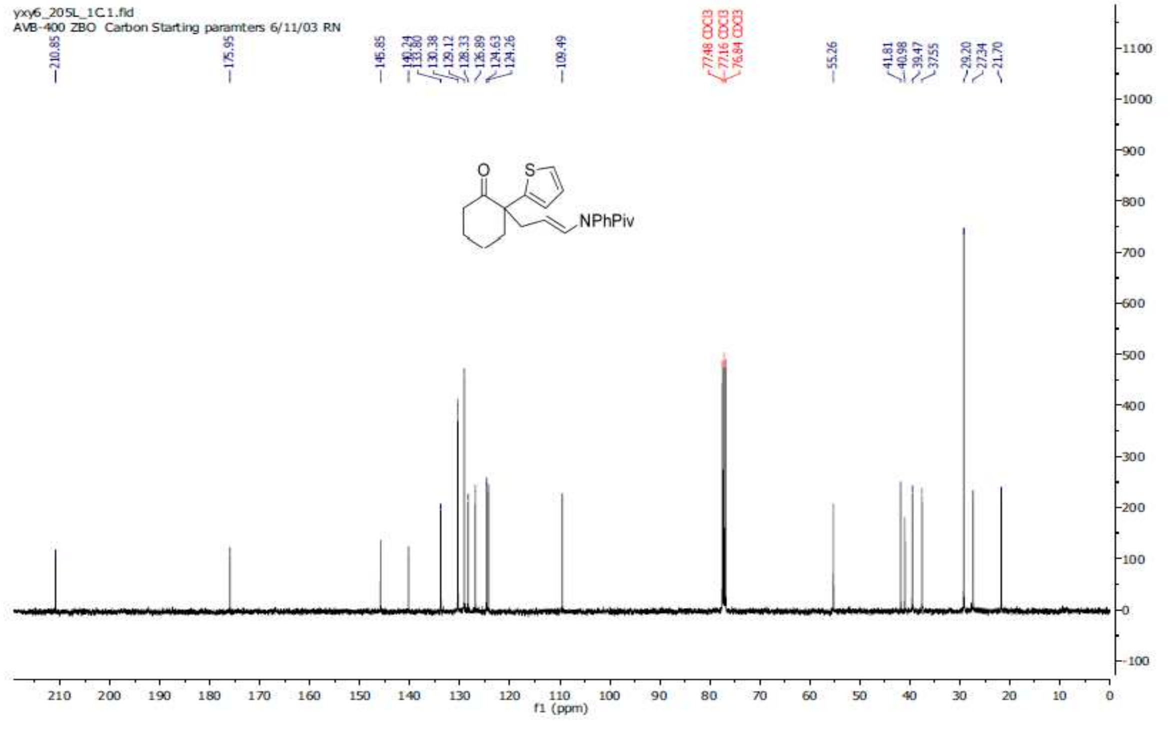

(*S,E*)-*N*-(3-(2-oxo-1-phenylcyclopentyl)prop-1-en-1-yl)-N-phenylpivalamide (**3k**)

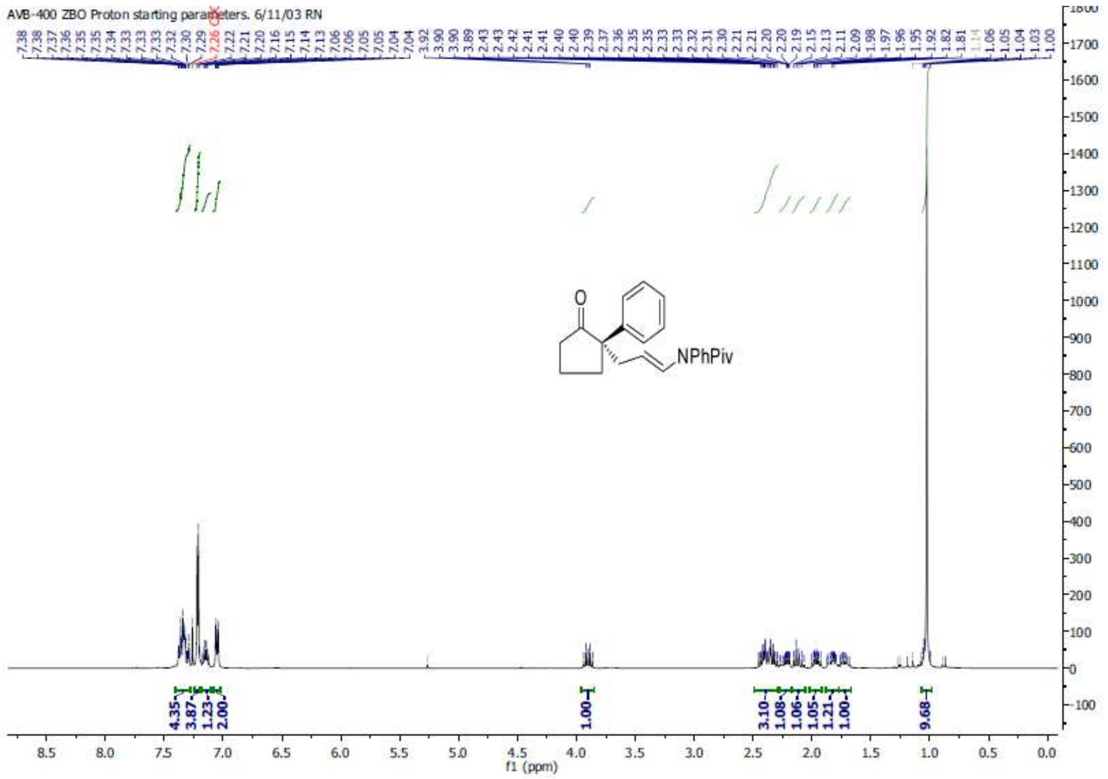

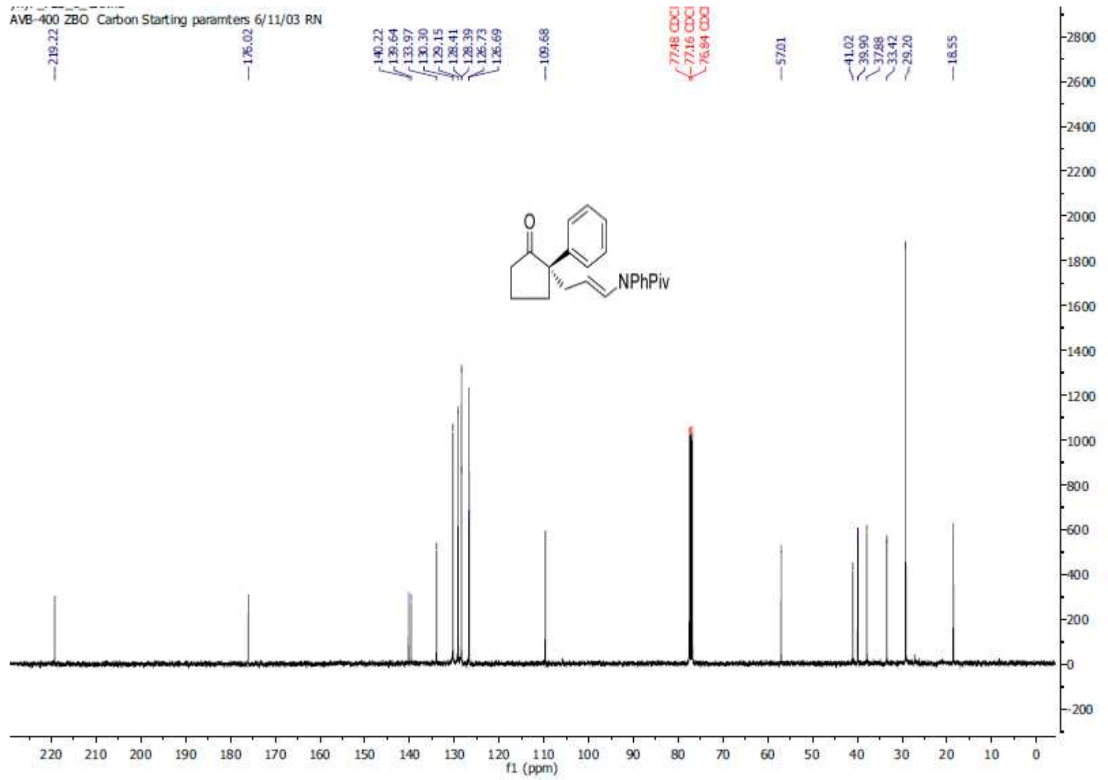

(*S,E*)-*N*-(3-(4-oxo-3-phenyltetrahydro-2H-pyran-3-yl)prop-1-en-1-yl)-*N*-phenylpivalamide (**3l**)

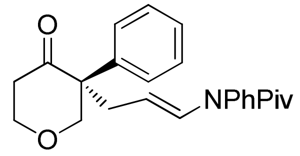

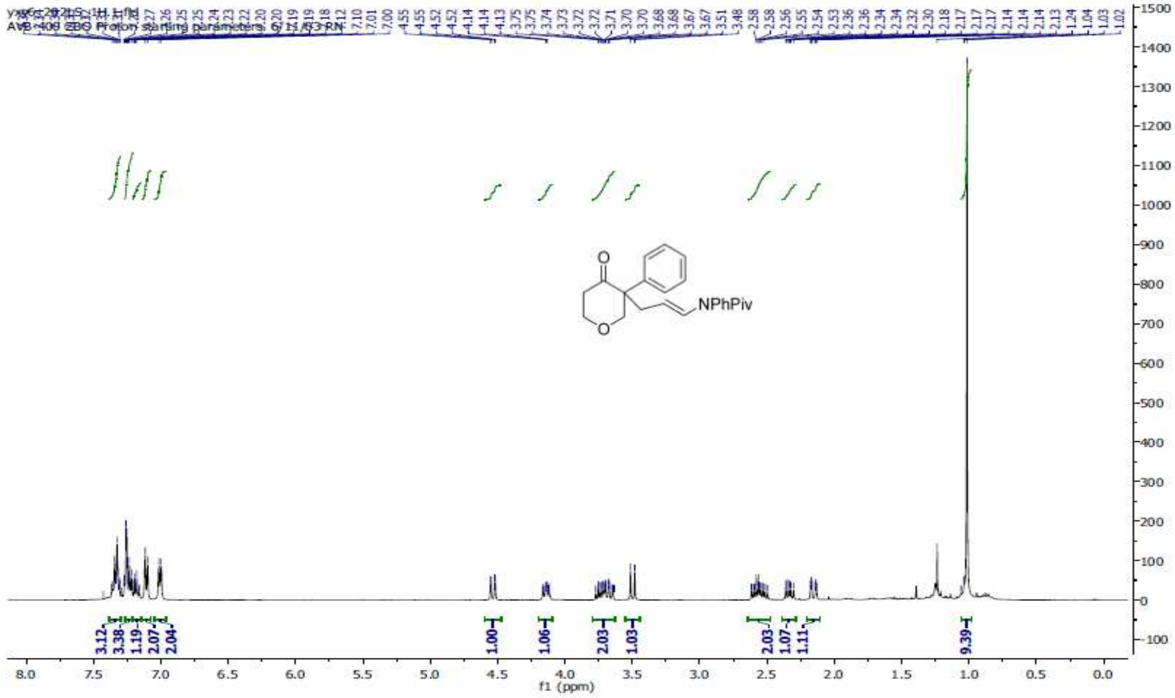

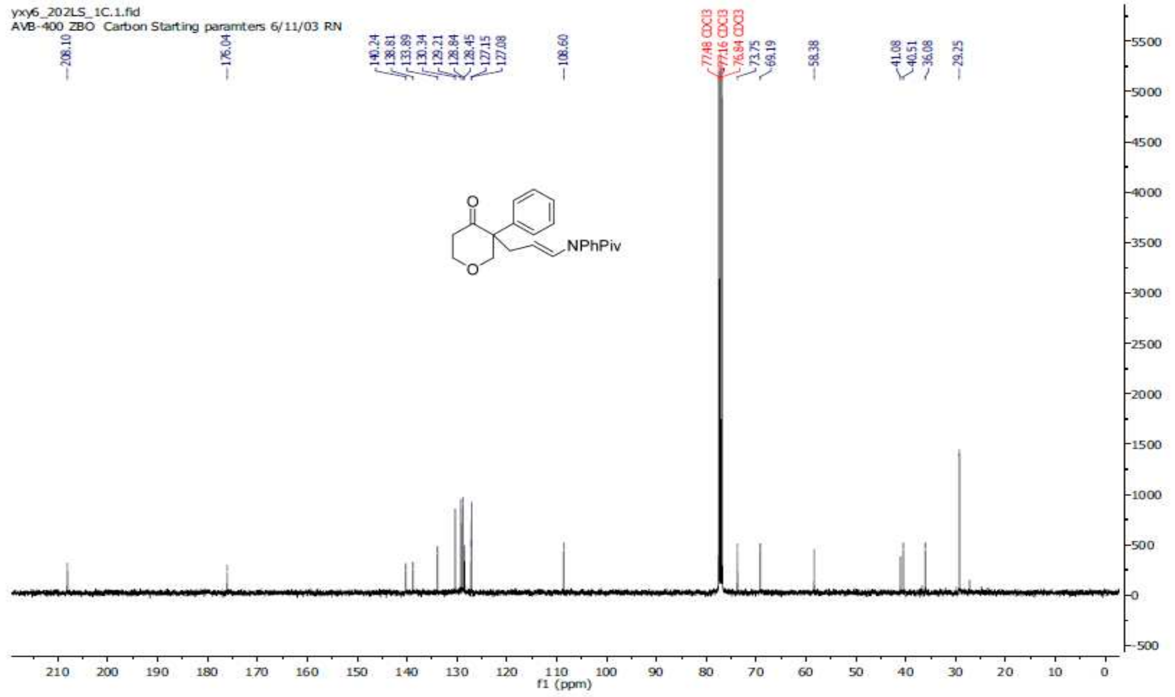

N-((*E*)-3-((*S*)-2-oxo-1-((*E*)-styryl)cyclohexyl)prop-1-en-1-yl)-*N*-phenylpivalamide (**3m**)

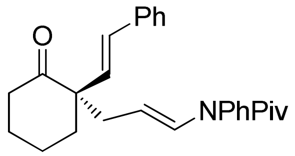

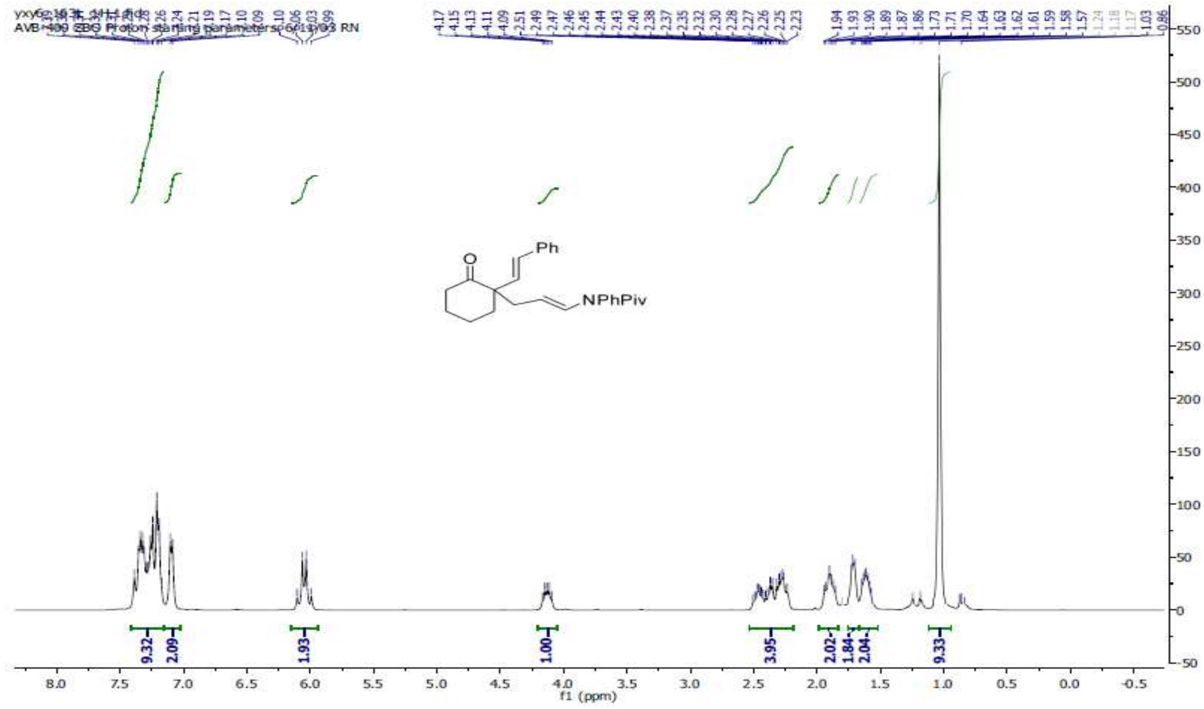

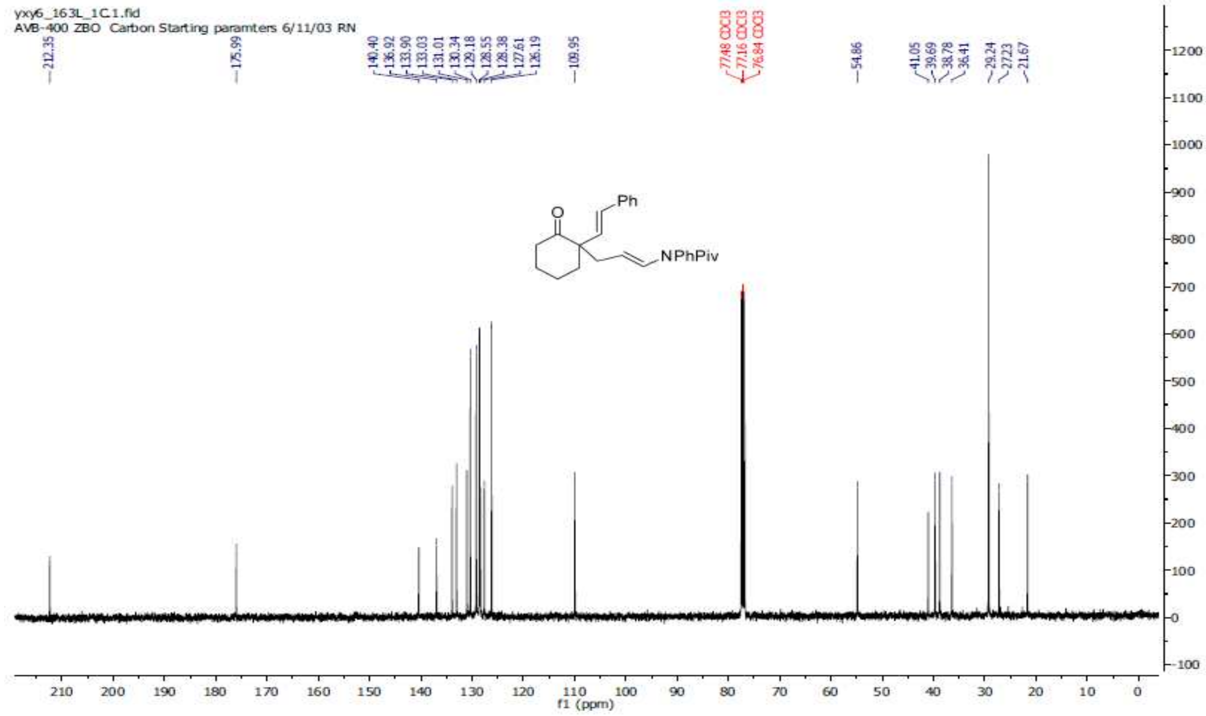

N-((*E*)-3-((*S*)-2-oxo-1-((*Z*)-styryl)cyclohexyl)prop-1-en-1-yl)-*N*-phenylpivalamide (**3n**)

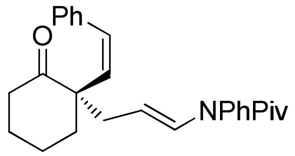

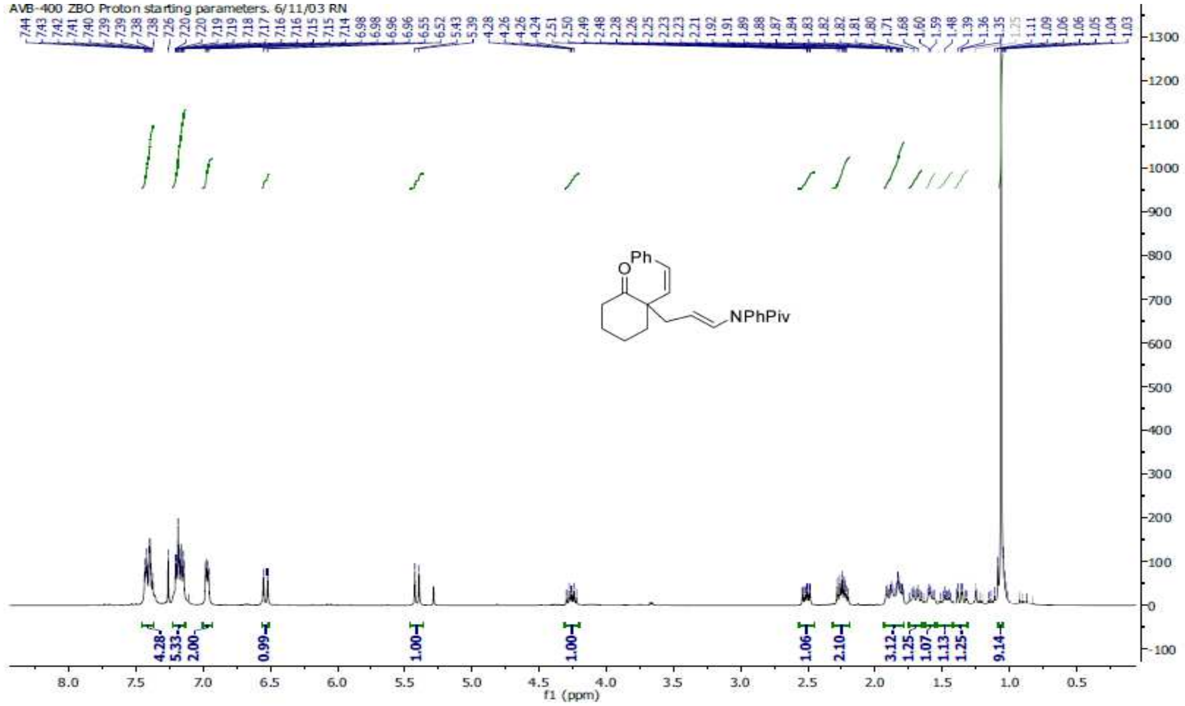

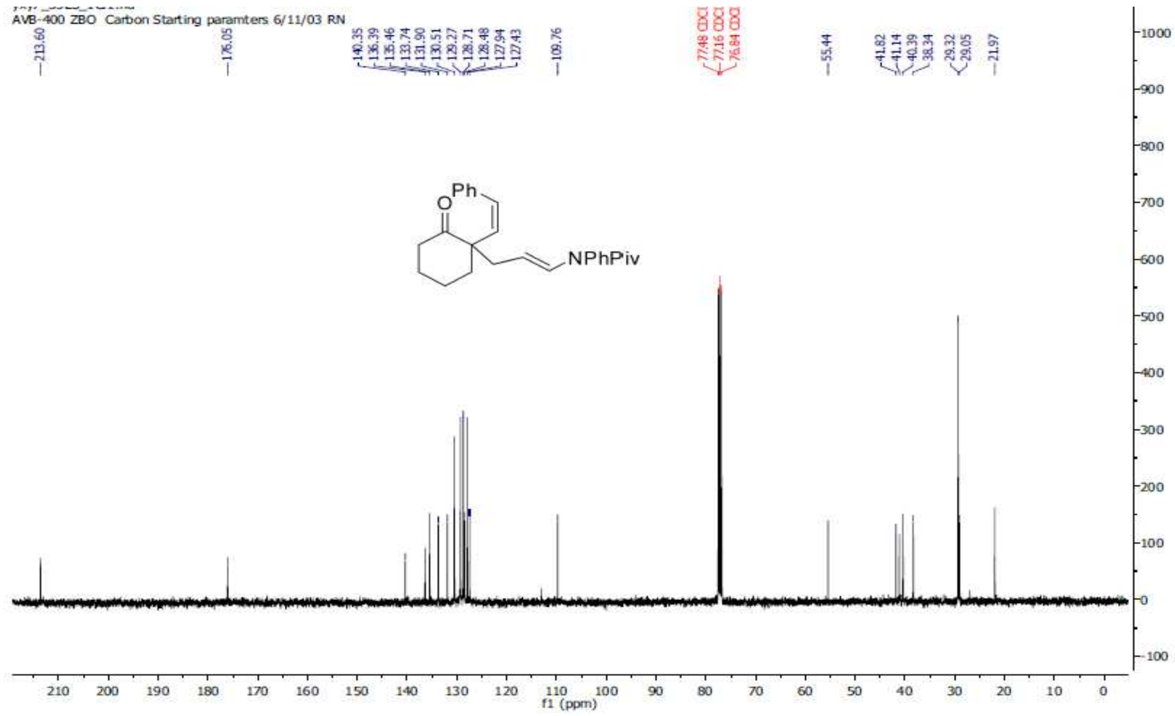

(*S,E*)-*N*-(3-(2-oxo-1-vinylcyclohexyl)prop-1-en-1-yl)-*N*-phenylpivalamide (**3o**)

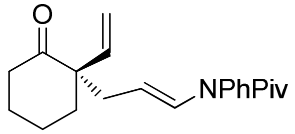

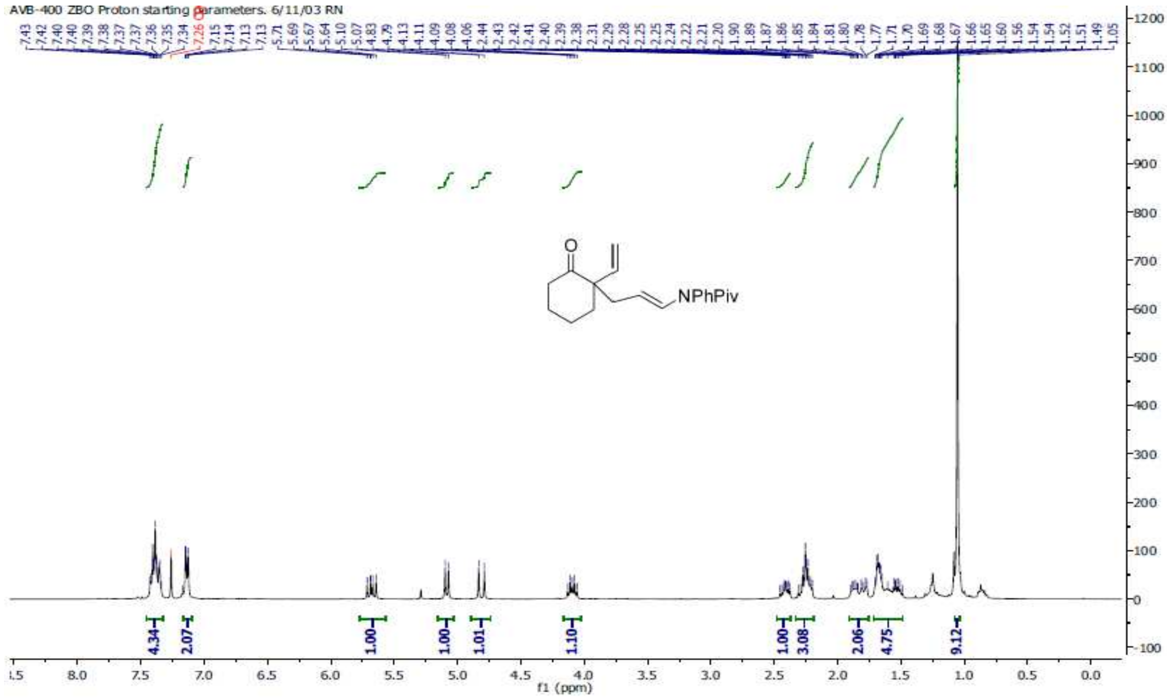

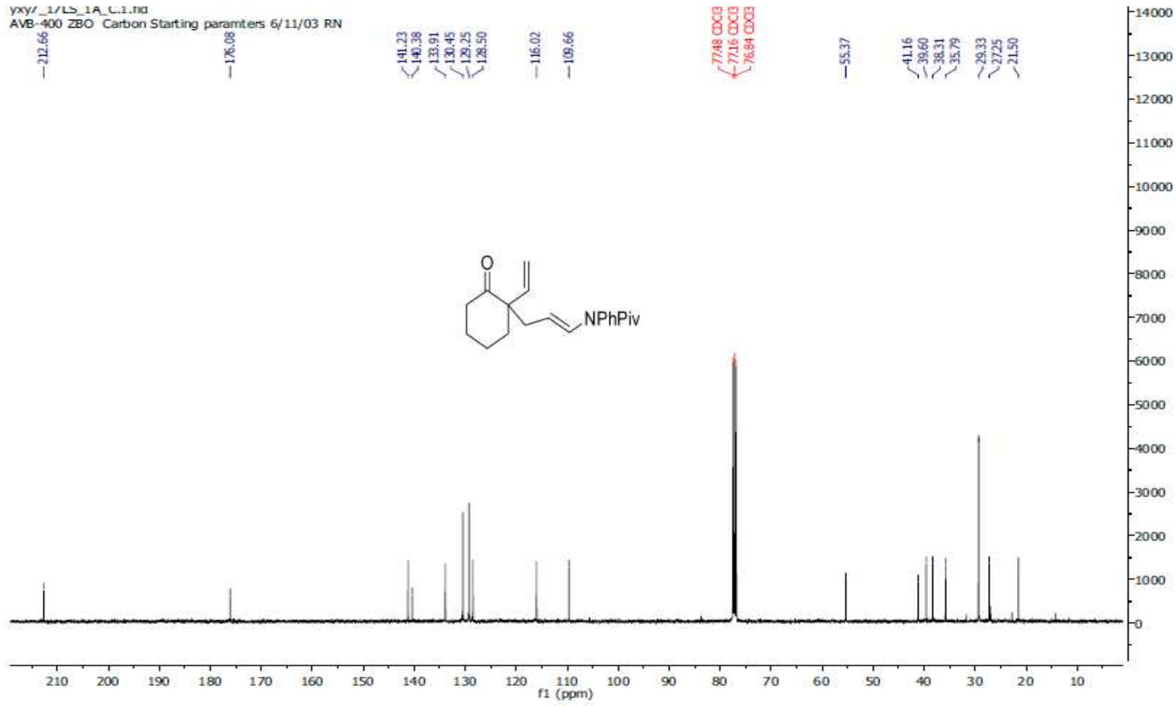

(*S*,*E*)-*N*-(3-(1-(2-methylprop-1-en-1-yl)-2-oxocyclohexyl)prop-1-en-1-yl)-*N*-phenylpivalamide (**3p**)

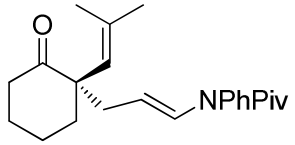

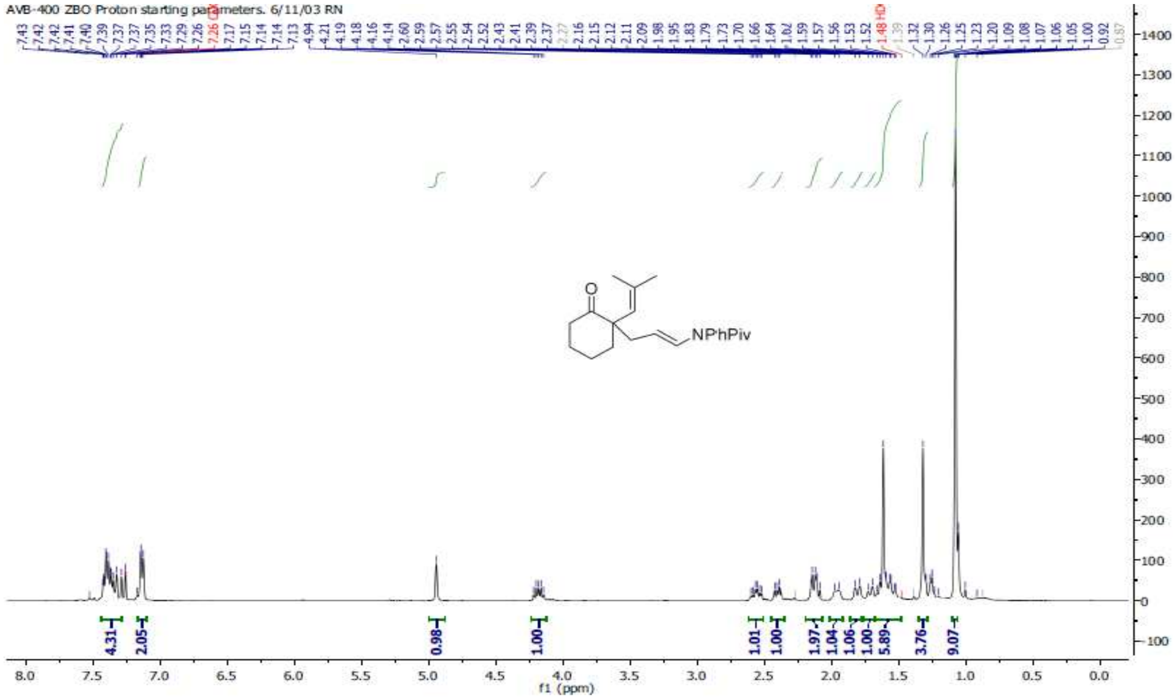

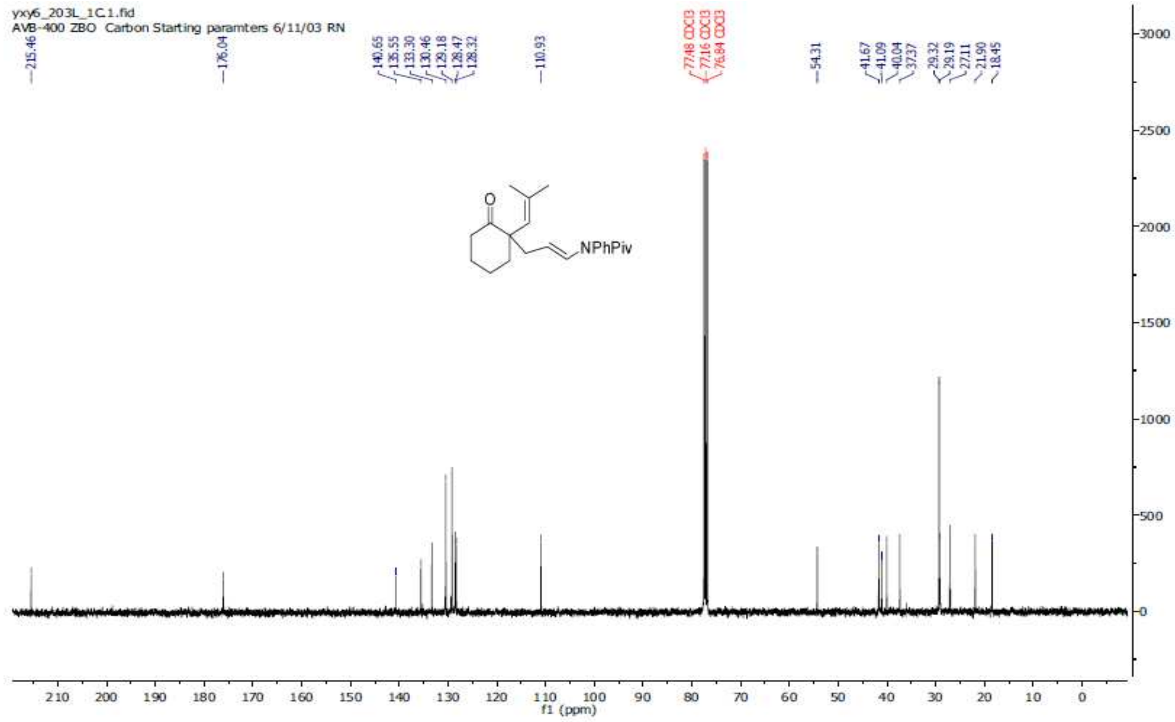

(*S*,*E*)-*N*-(3-(2-oxo-[1,1'-bi(cyclohexan)]-1'-en-1-yl)prop-1-en-1-yl)-*N*-phenylpivalamide (**3q**)

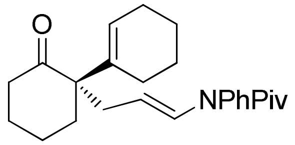

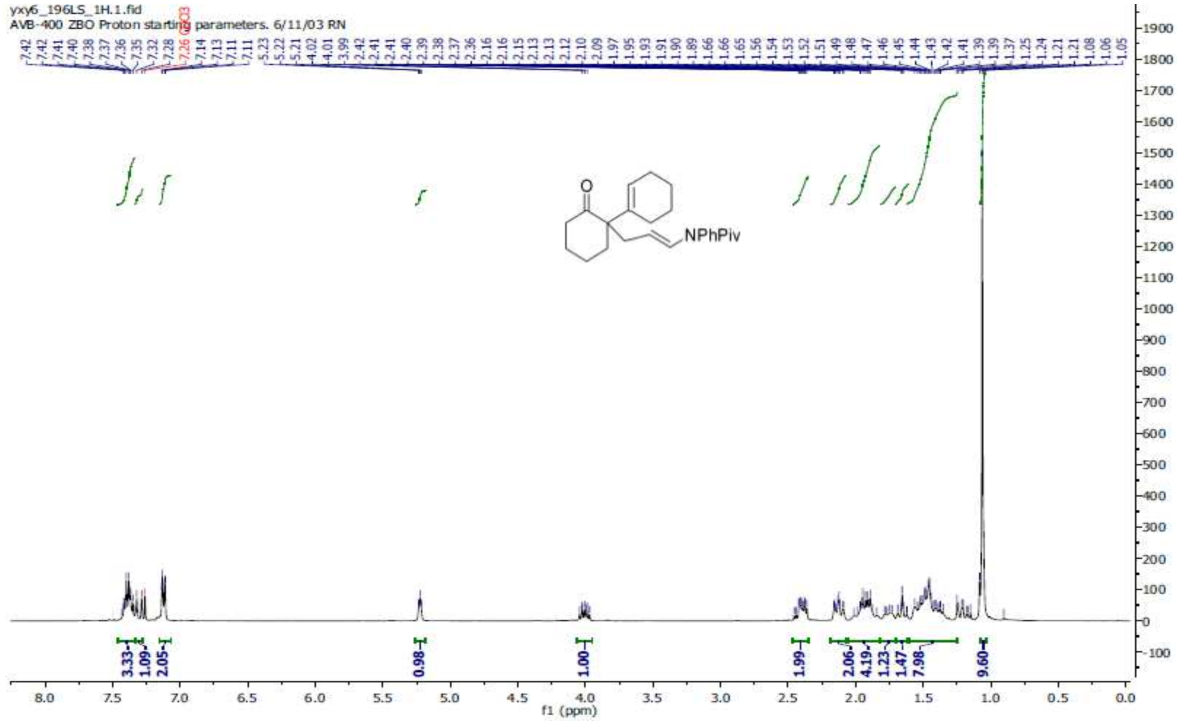

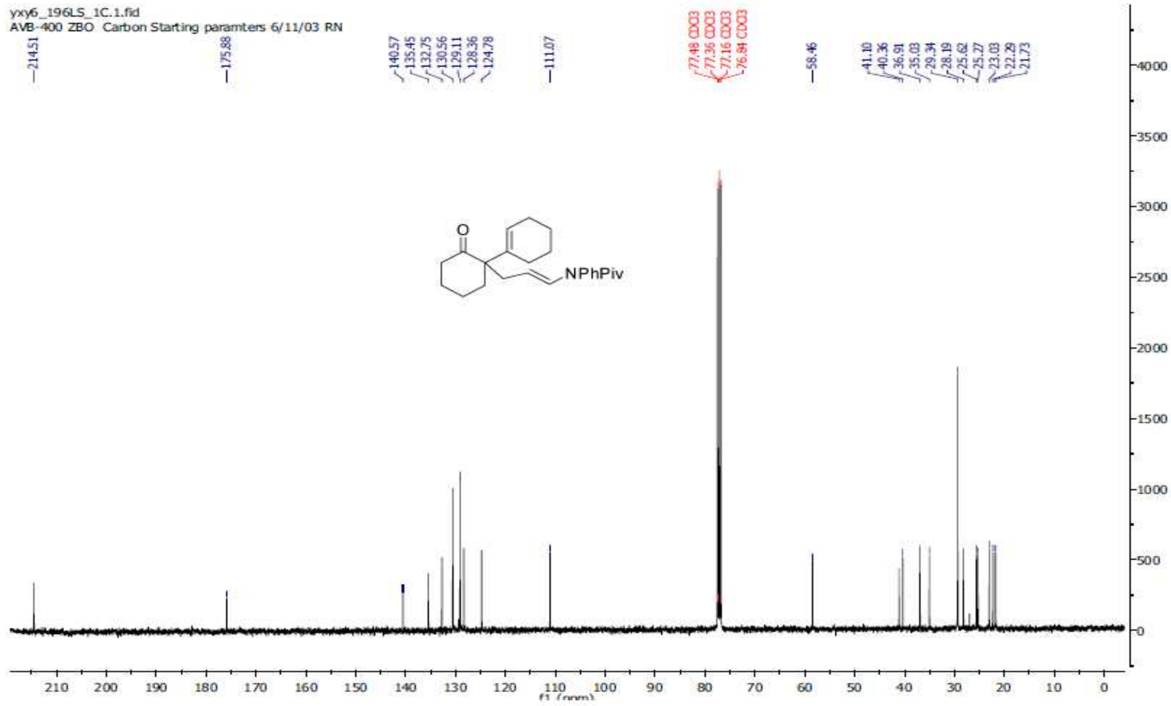

(*S*,*E*)-*N*-(3-(2-oxo-1-(phenylethynyl)cyclohexyl)prop-1-en-1-yl)-*N*-phenylpivalamide (**3r**)

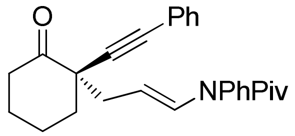

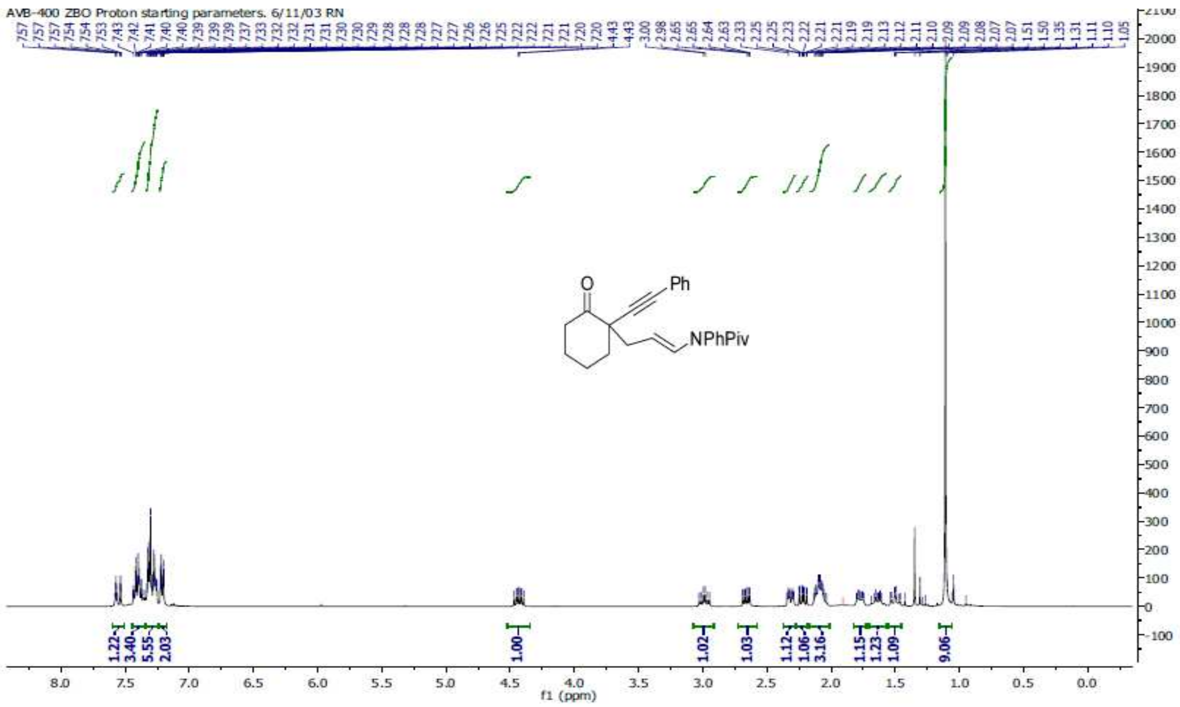

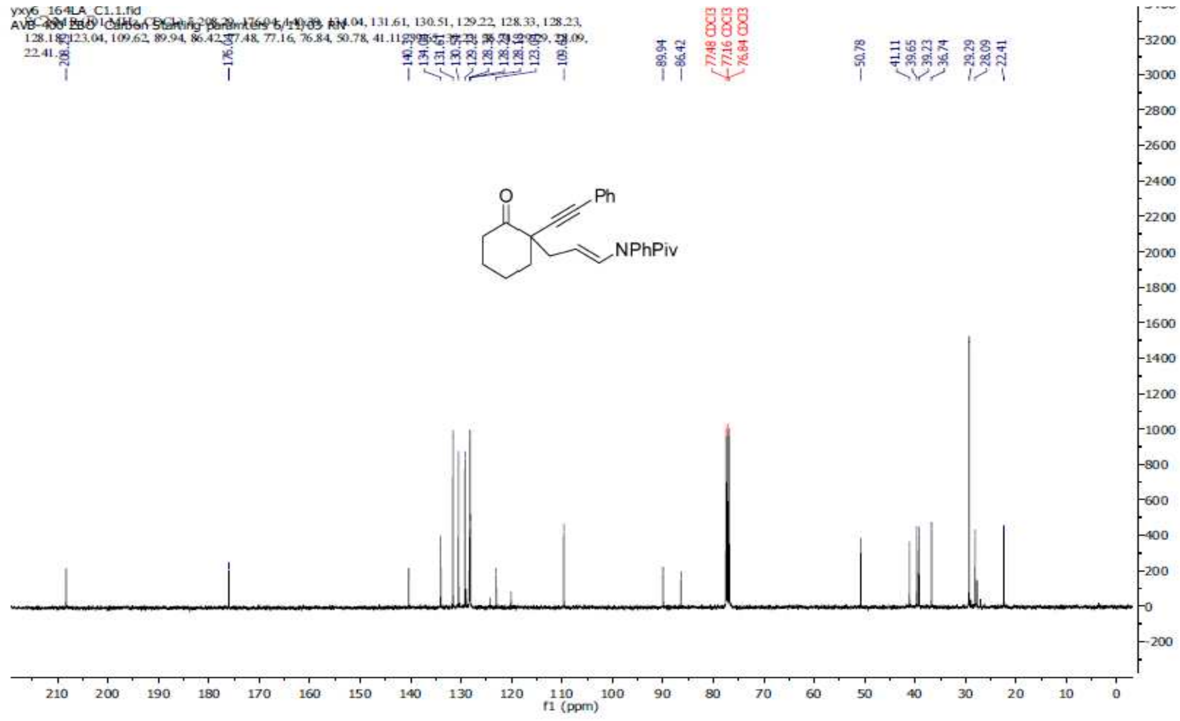

(*S*,*E*)-*N*-(3-(1-(hex-1-yn-1-yl)-2-oxocyclohexyl)prop-1-en-1-yl)-*N*-phenylpivalamide (**3s**)

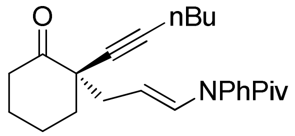

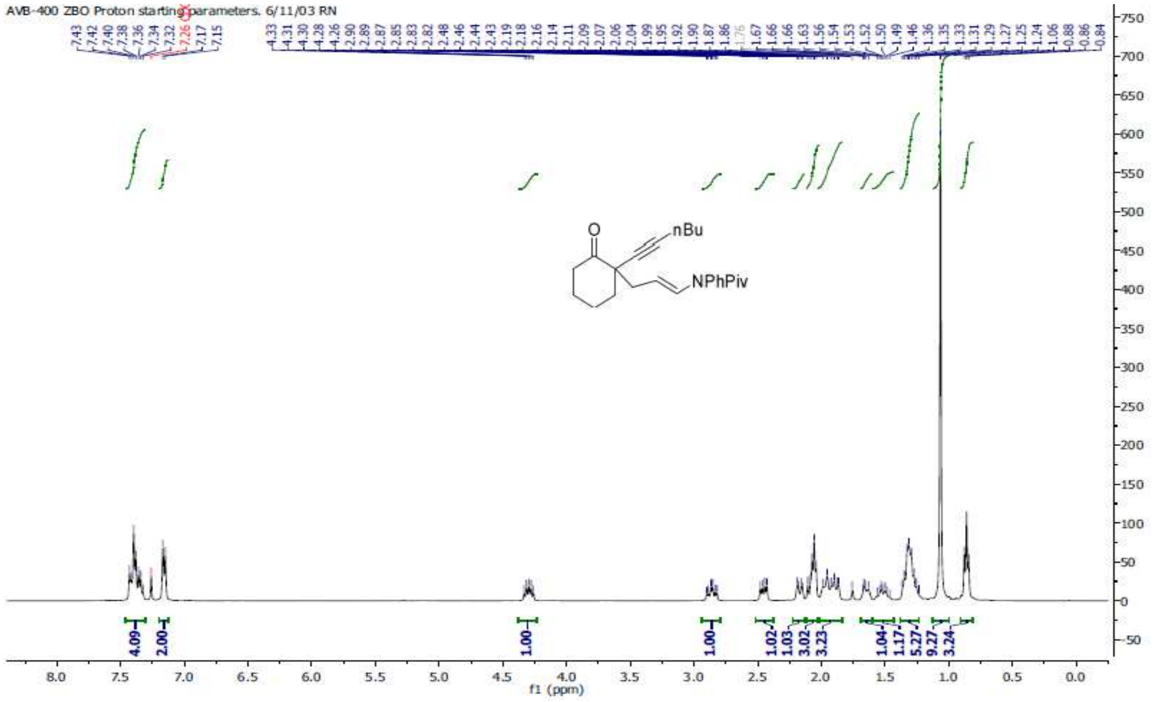

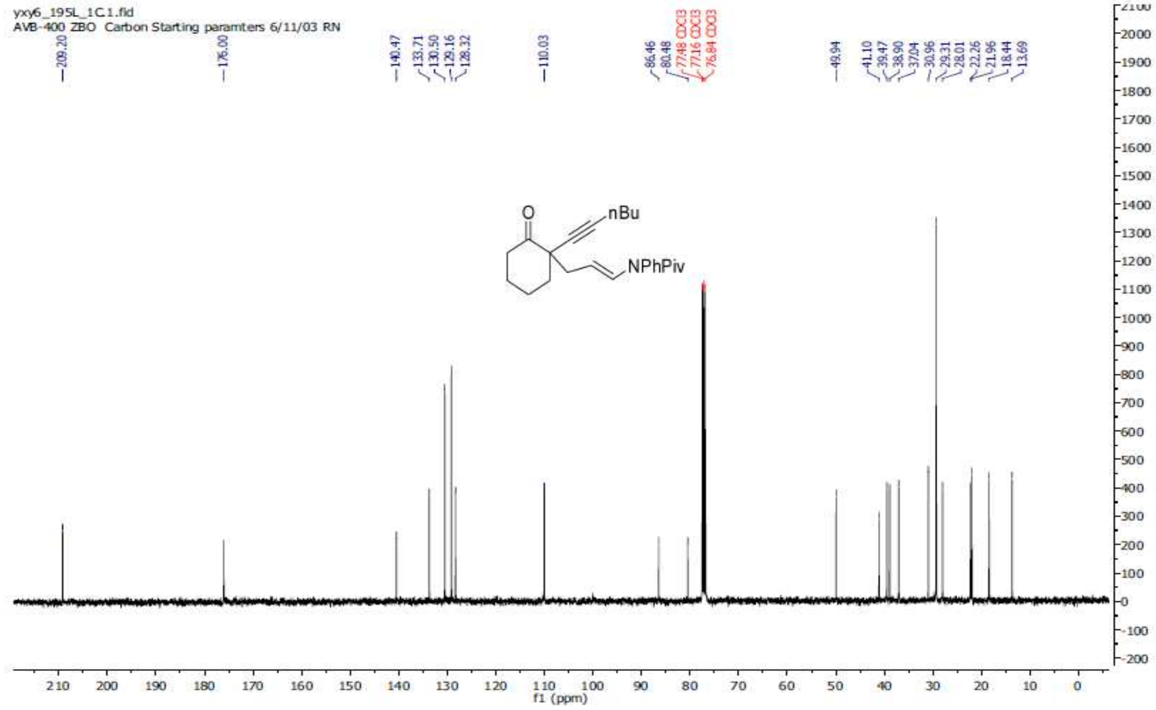

(*S*,*E*)-*N*-(3-(1-methyl-2-oxocyclohexyl)prop-1-en-1-yl)-*N*-phenylpivalamide (**3t**)

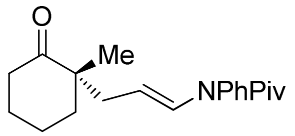

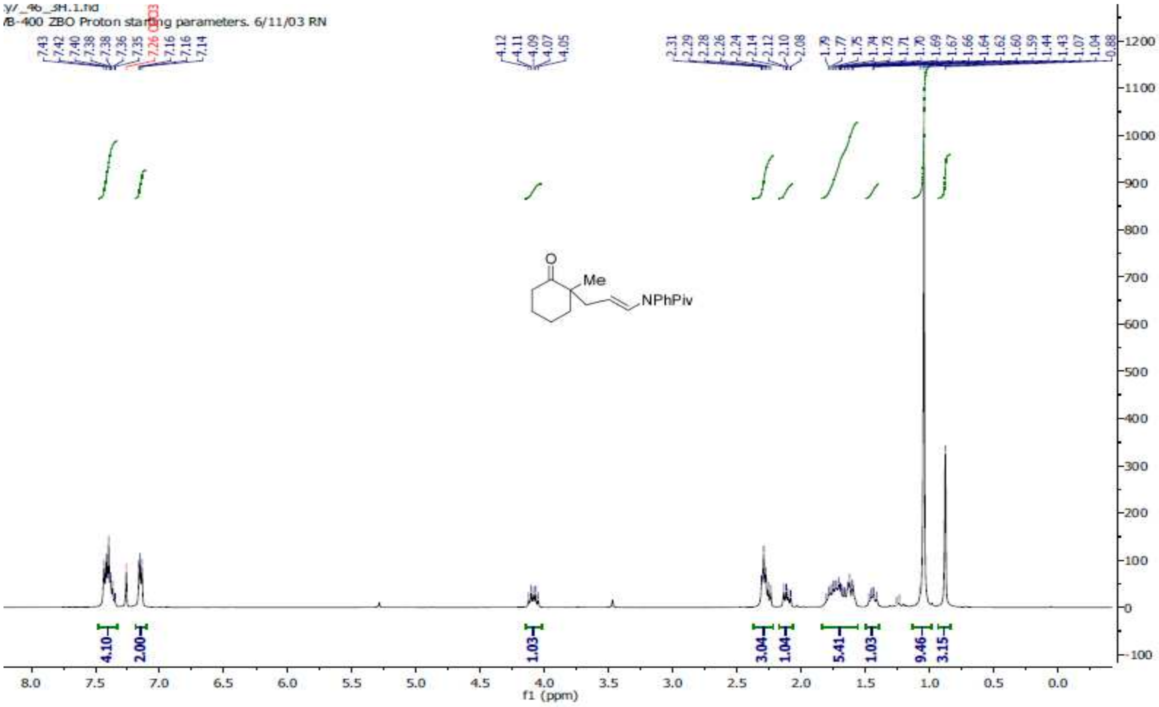

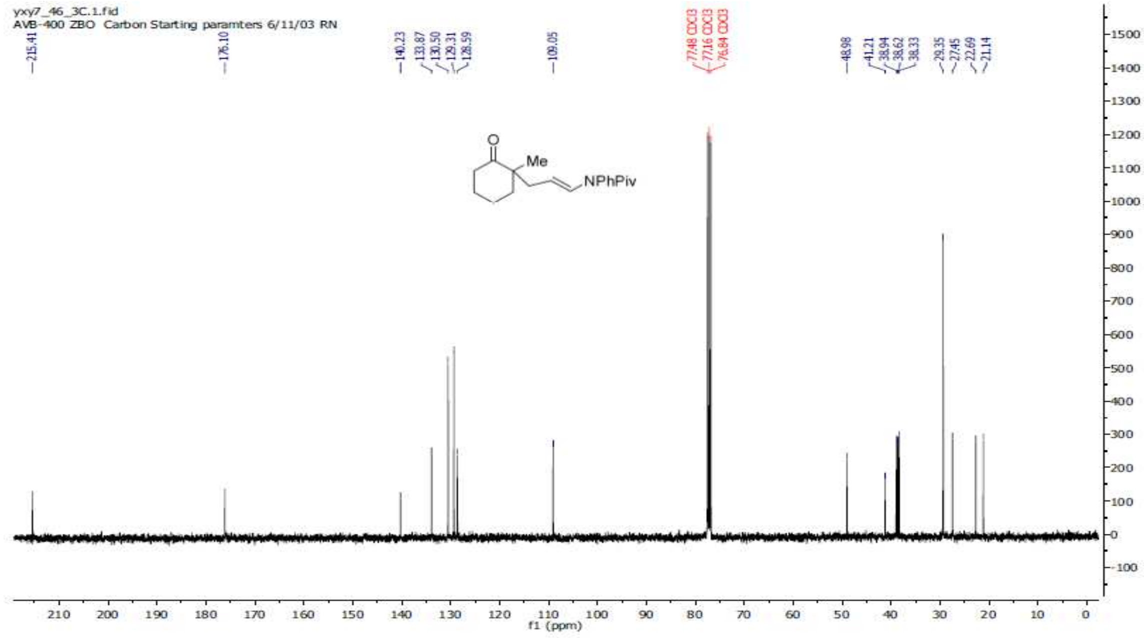

(*S*,*E*)-*N*-(3-(1-butyl-2-oxocyclohexyl)prop-1-en-1-yl)-*N*-phenylpivalamide (**3u**)

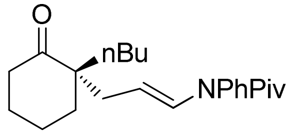

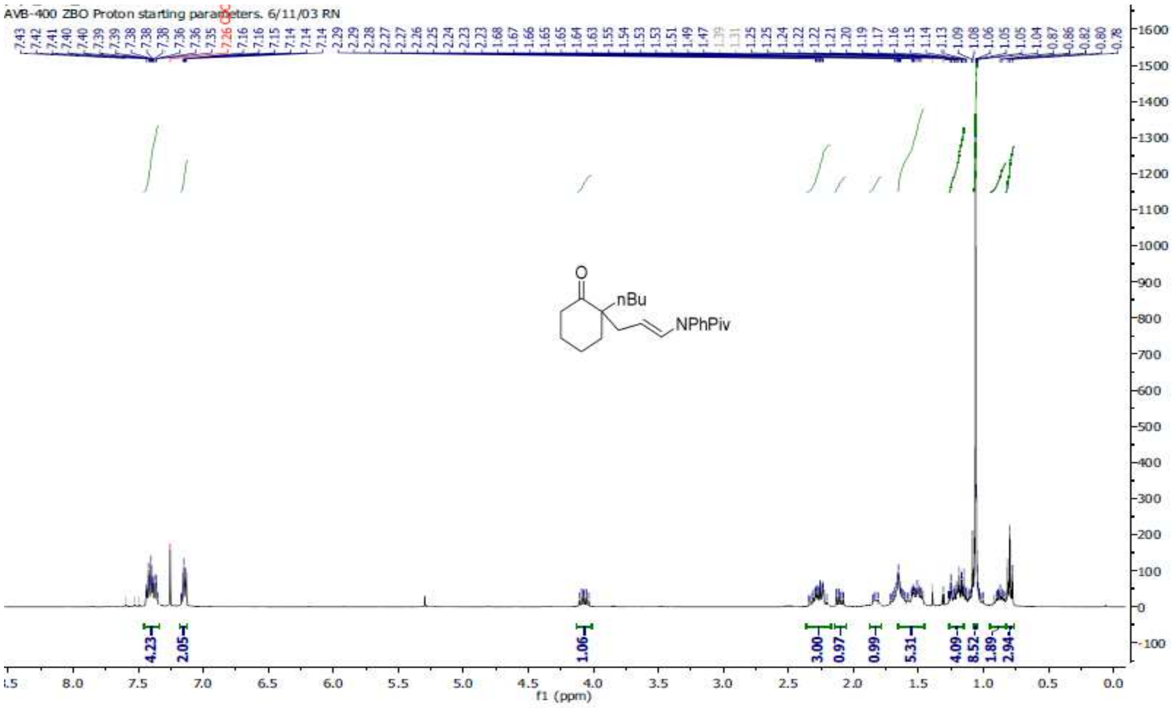

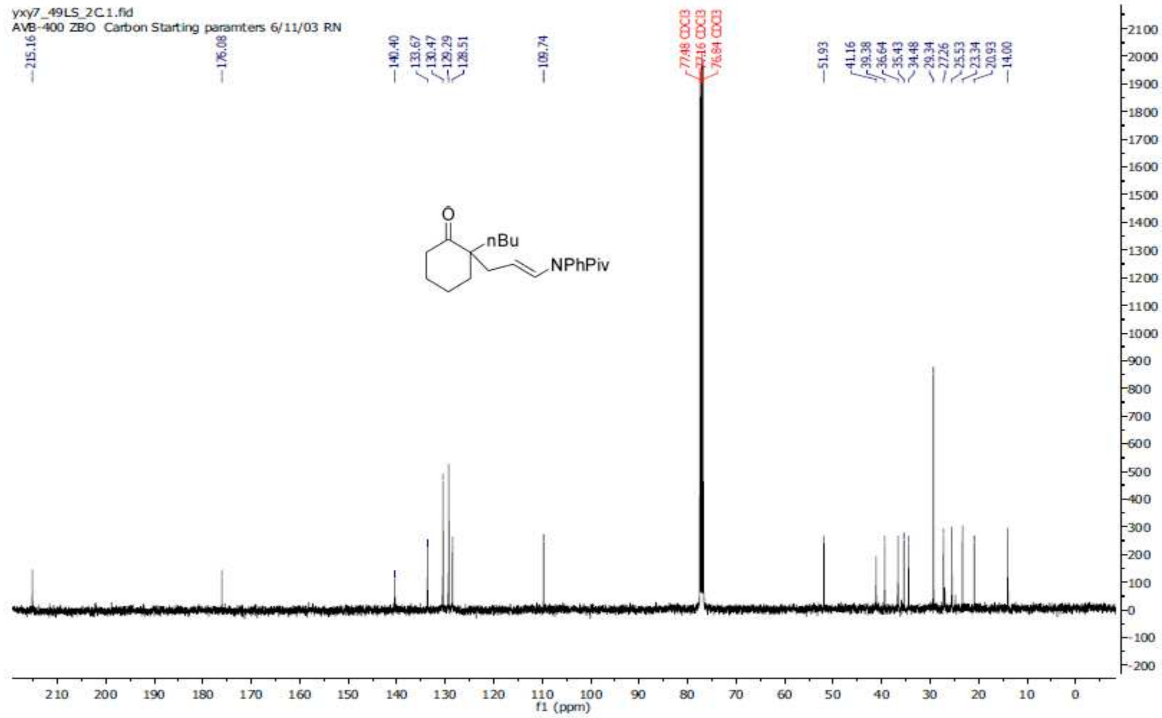

(*S*)-3-(2-oxo-1-phenylcyclohexyl)propanal (**4a**)

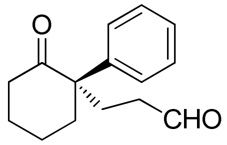

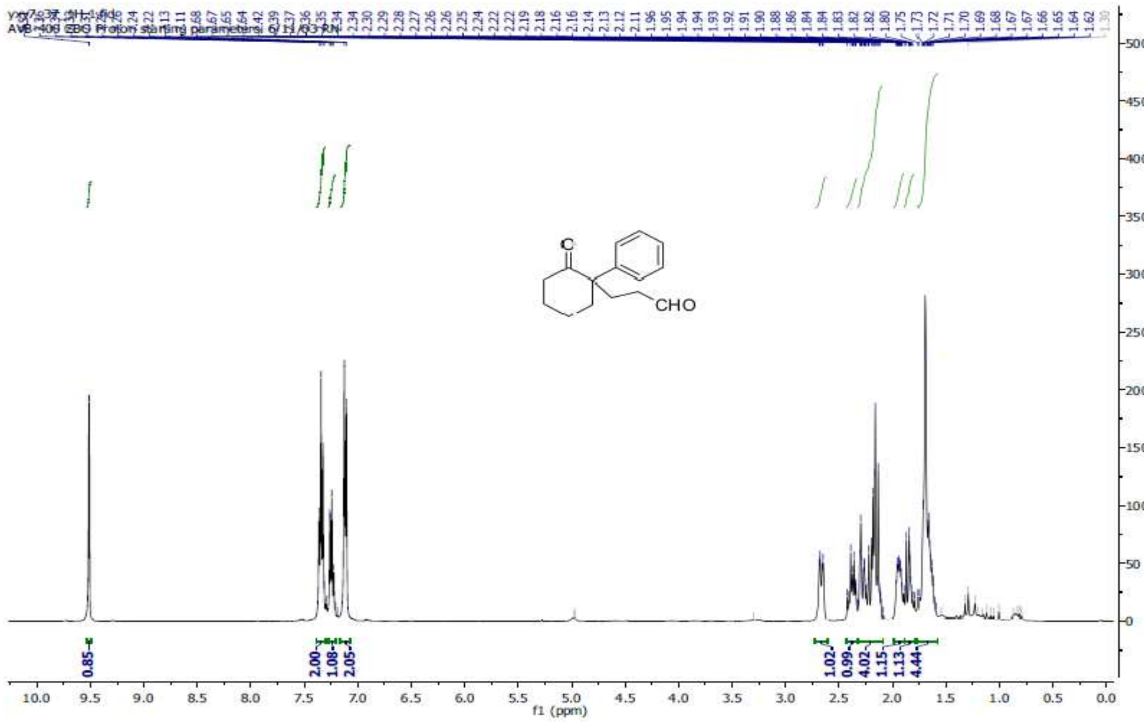

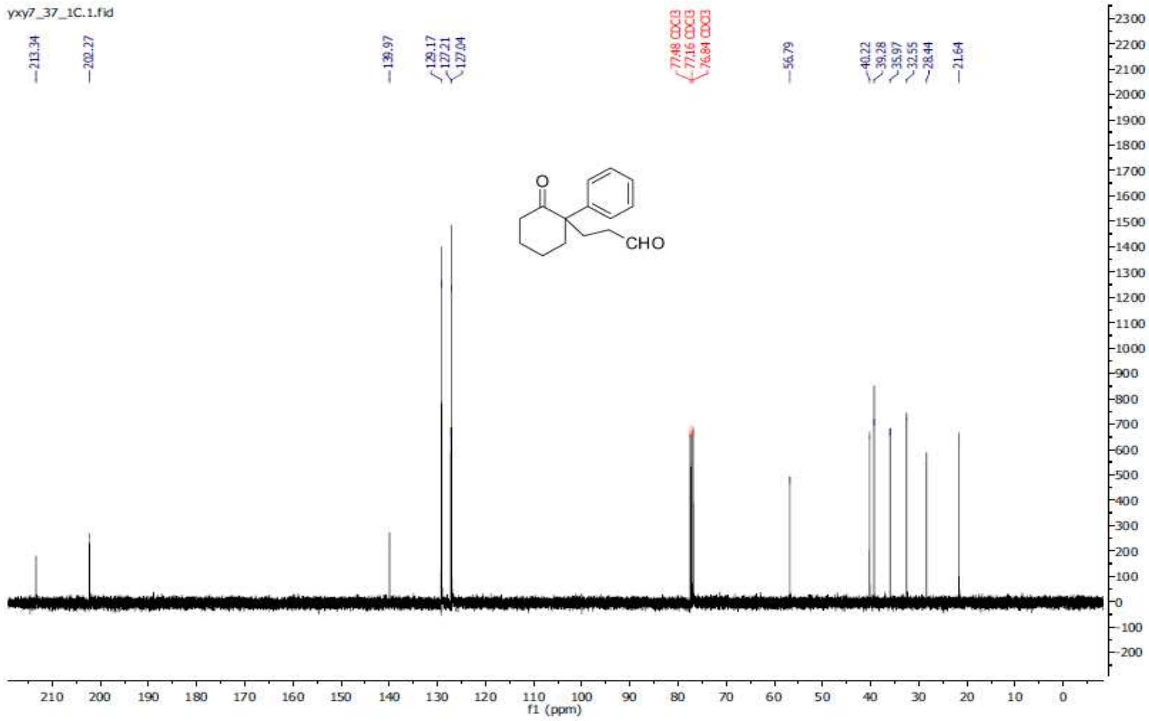

(*1R,5S*)-5-phenylbicyclo[3.3.1]nonane-2,9-dione (**5a**)

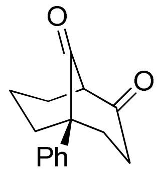

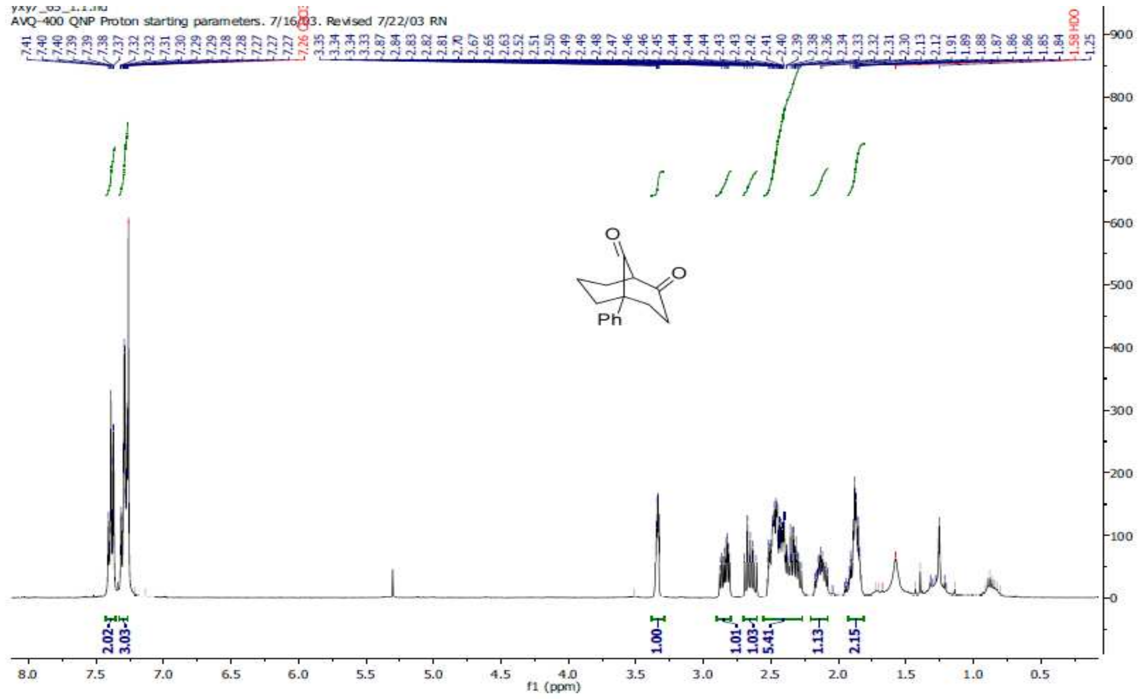

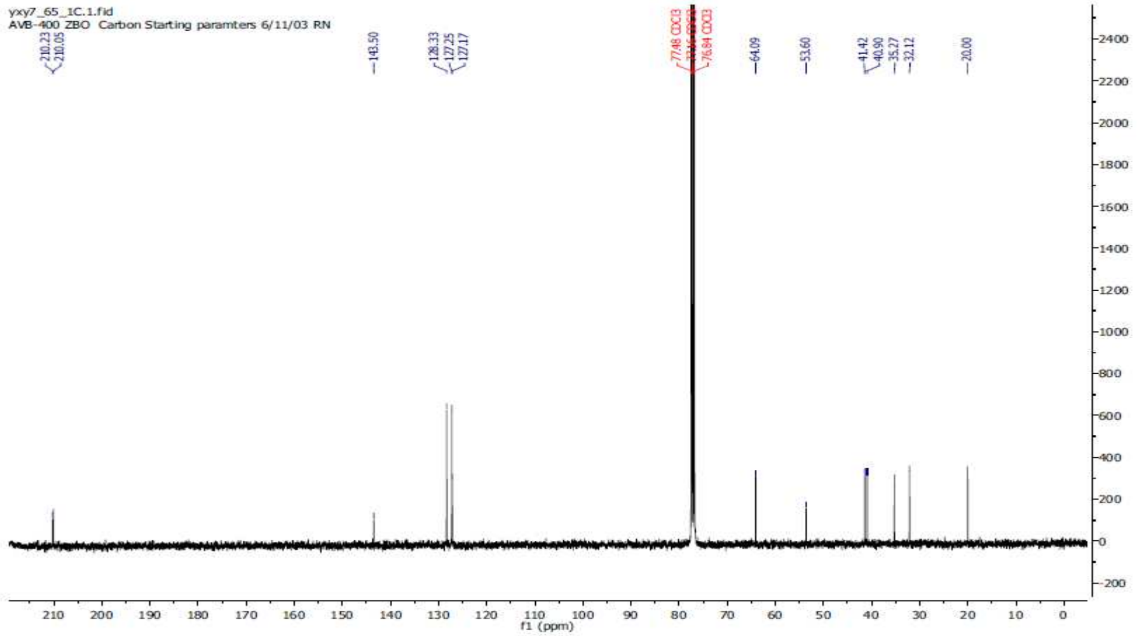

(4a*S*,8aS)-4a-phenyloctahydro-2H-chromen-2-one (**6a**)

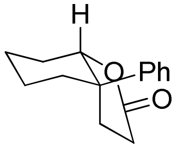

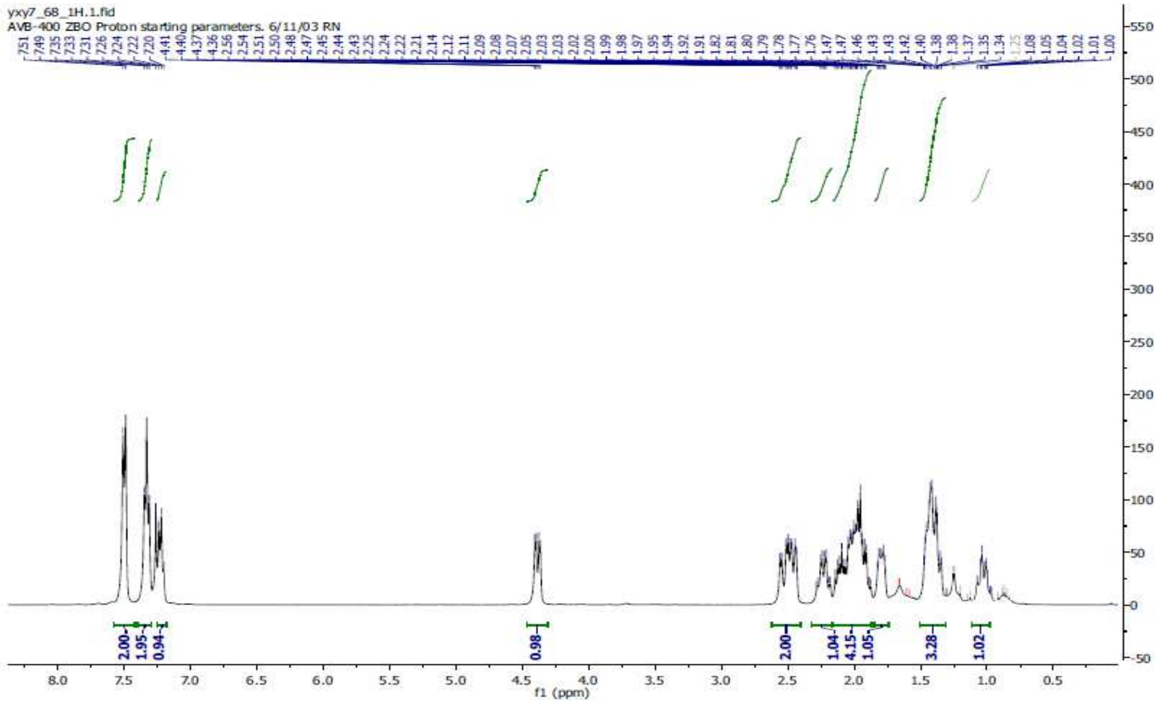

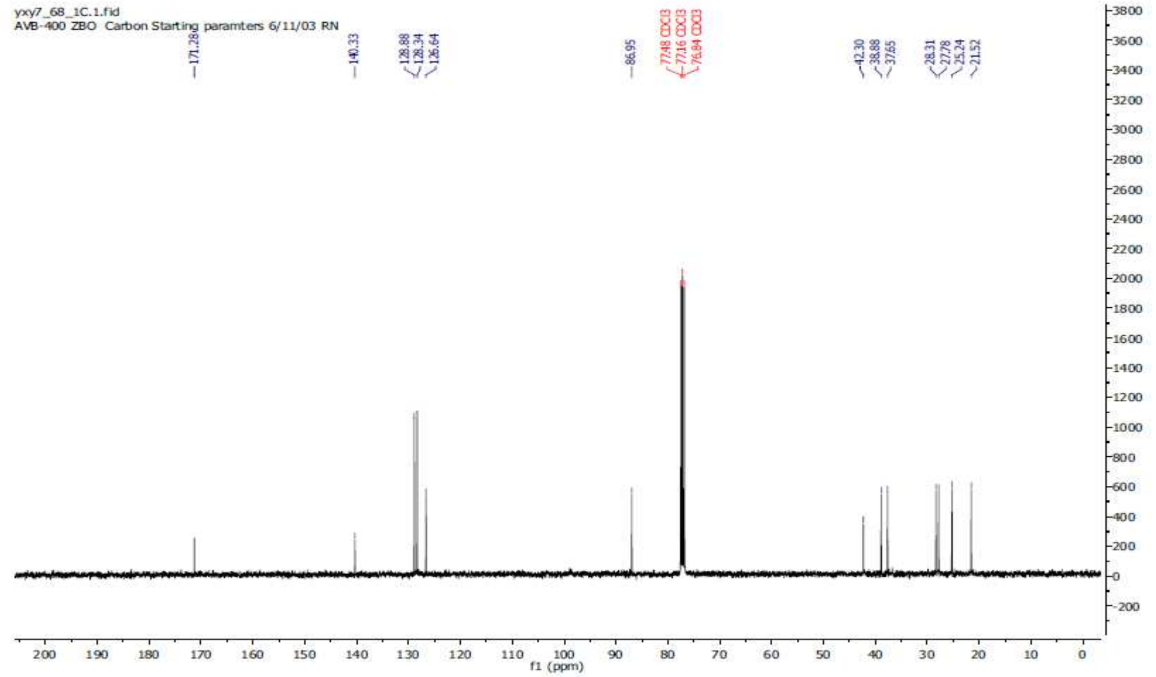

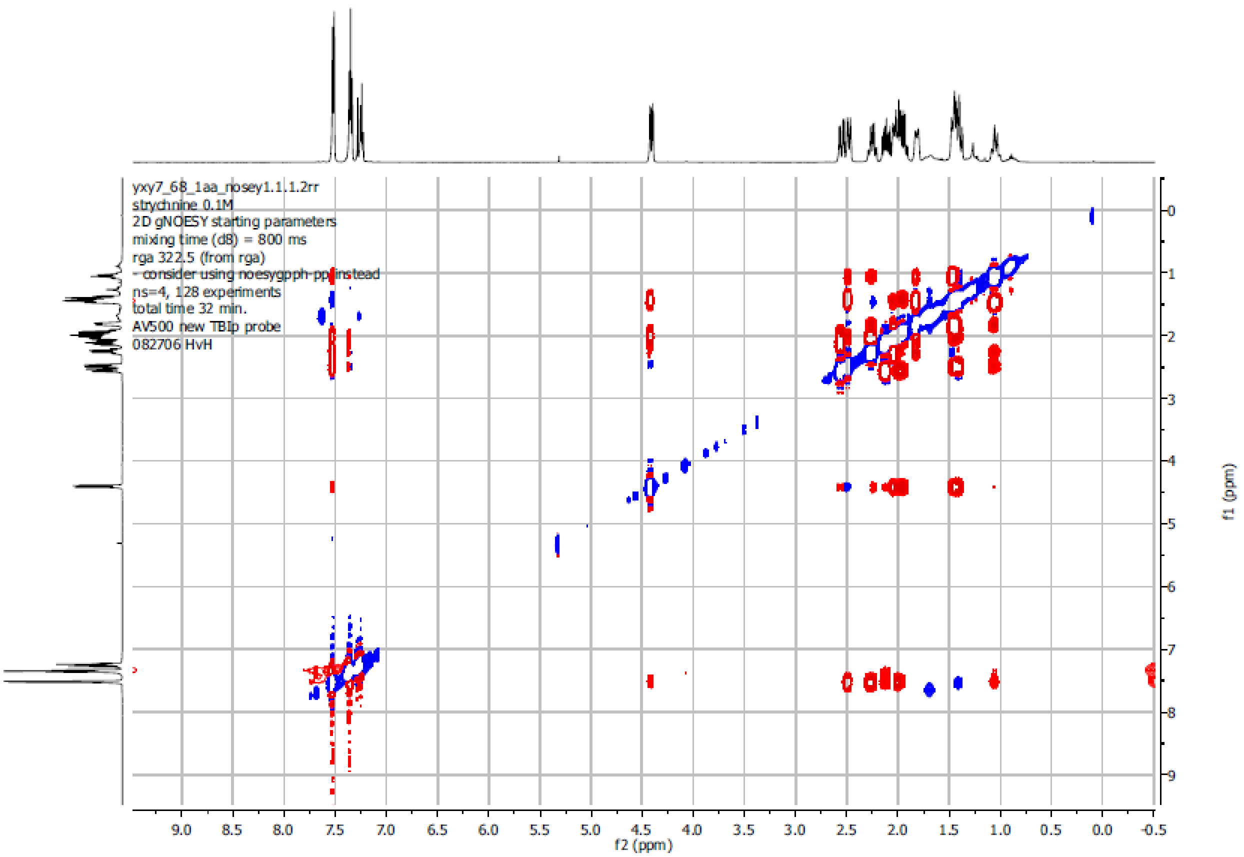
yxy7_68_1aa_nosey1.1.1.2rr
strychnine 0.1M
2D gNOESY starting parameters
mixing time (d8) = 800 ms
rga 322.5 (from rga)
- consider using noesygpph-pp instead
ns=4, 128 experiments
total time 32 min.
AV500 new TBIp probe
082706 HvH
f2 (ppm)
f1 (ppm)

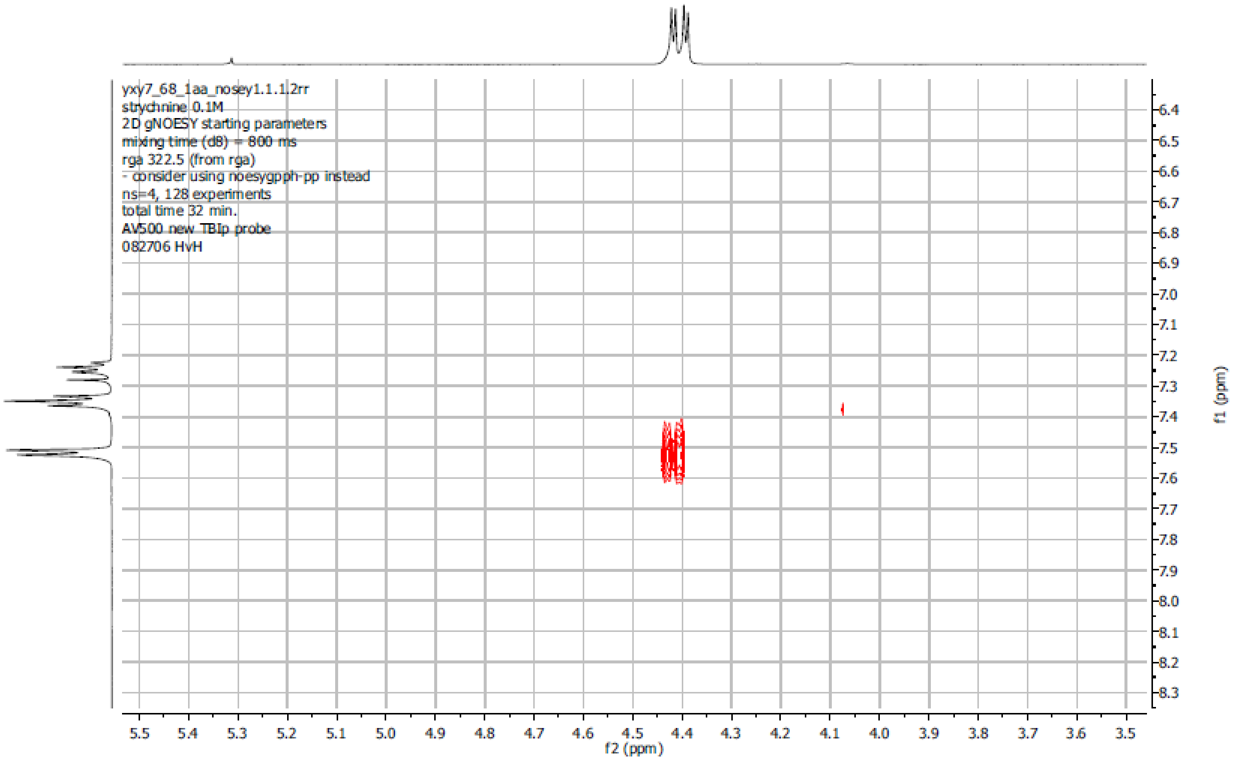
yxy7_68_1aa_nosey1.1.1.2rr
strychnine 0.1M
2D gNOESY starting parameters
mixing time (d8) = 800 ms
rga 322.5 (from rga)
- consider using noesygpph-pp instead
ns=4, 128 experiments
total time 32 min.
AV500 new TBIp probe
082706 HvH
f2 (ppm)
f1 (ppm)

(3a*S*,7a*S*)-3a-phenyloctahydro-1H-indole (**8a**)

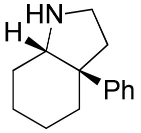

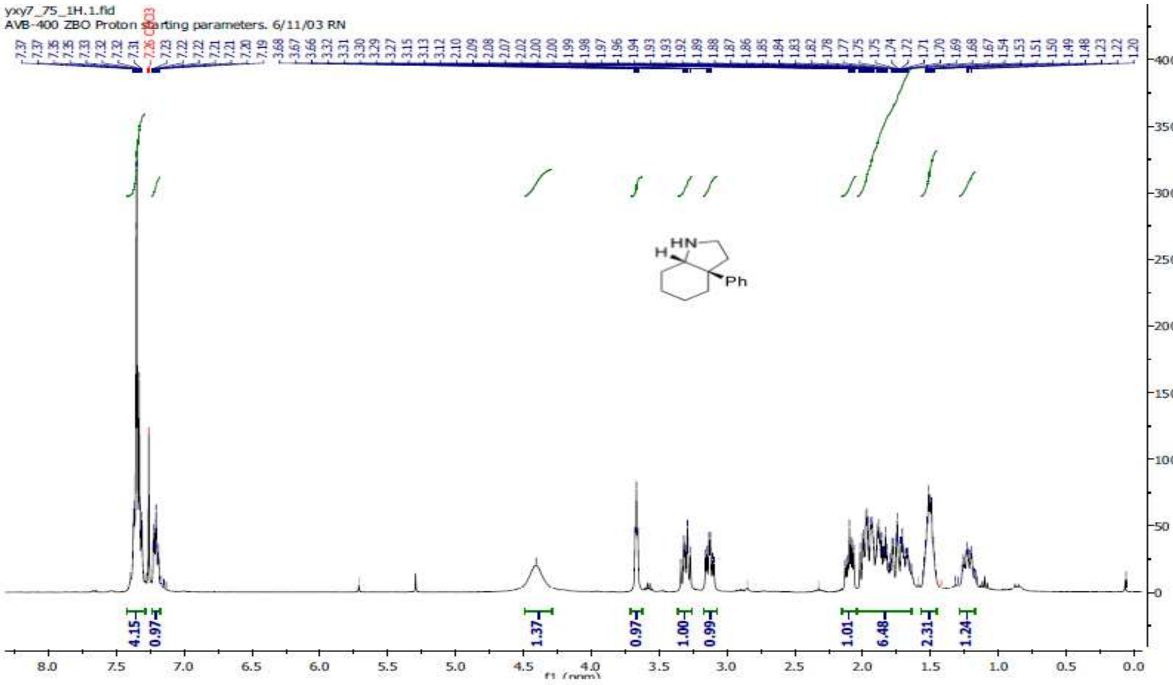

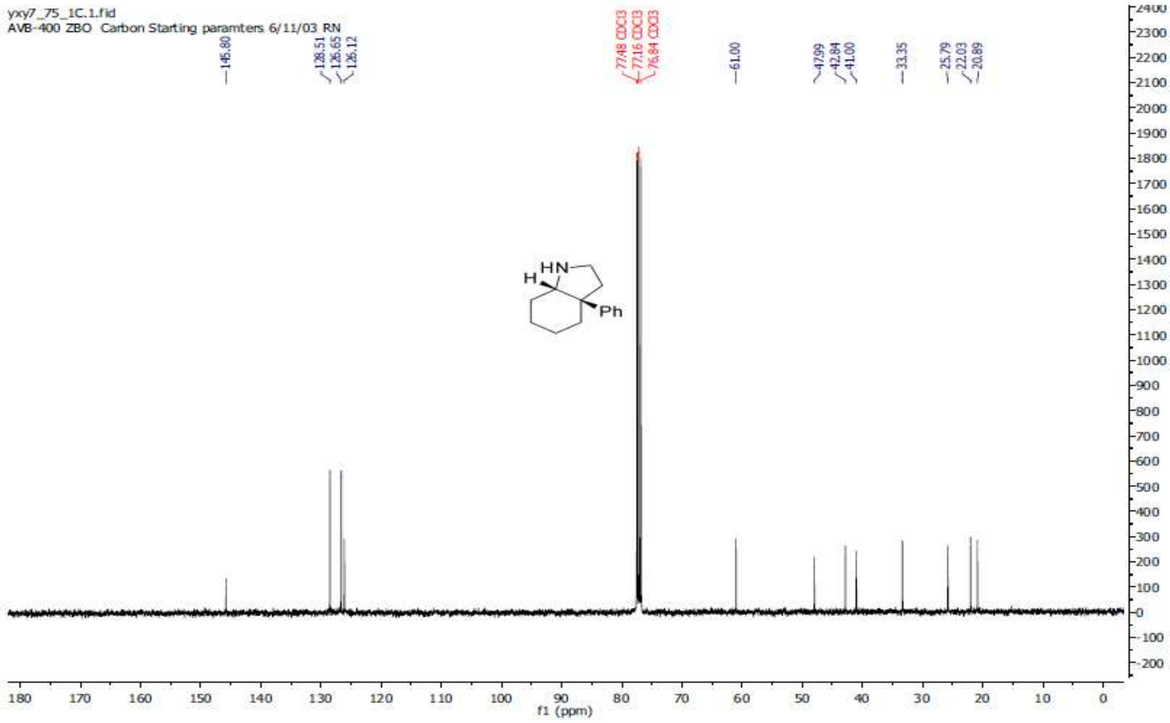

Supplement: Supplementary file 1 [file SC-007-C5SC04202J-s001.pdf]
